# Development and characterization of fluorescent cholesteryl probes with enhanced solvatochromic and pH-sensitive properties for live-cell imaging

**DOI:** 10.1038/s41598-024-80958-2

**Published:** 2024-12-28

**Authors:** Vicente Rubio, Nicholas McInchak, Genesis Fernandez, Dana Benavides, Diana Herrera, Catherine Jimenez, Haylee Mesa, Jonathan Meade, Qi Zhang, Maciej J. Stawikowski

**Affiliations:** 1 Department of Chemistry and Biochemistry, Charles E. Schmidt College of Science, Florida Atlantic University, 777 Glades Rd, Boca Raton, FL 33431 USA; 2 Stiles-Nicholson Brain Institute, Florida Atlantic University, 5353 Parkside Dr, Jupiter, FL 33458 USA

**Keywords:** Cholesterol, Fluorescent reporter, pH-sensitivity, Fluorescence microscopy, Membrane, Lipid rafts, Lipid droplets, Lysosomes., Biochemistry, Sterols, Sterols, Fluorescent dyes

## Abstract

We present novel fluorescent cholesteryl probes (CNDs) with a modular design based on the solvatochromic 1,8-phthalimide scaffold. We have explored how different modules—linkers and head groups—affect the ability of these probes to integrate into lipid membranes and how they distribute intracellularly in mouse astrocytes and fibroblasts targeting lysosomes and lipid droplets. Each compound was assessed for its solvatochromic behavior in organic solvents and model membranes. Molecular dynamics simulations and lipid partitioning using giant unilamellar vesicles showed how these analogs behave in model membranes compared to cholesterol. Live-cell imaging demonstrated distinct staining patterns and cellular uptake behaviors, further validating the utility of these probes in biological systems. We compared the empirical results with those of BODIPY-cholesterol, a well-regarded fluorescent cholesterol analog. The internalization efficiency of fluorescent CND probes varies in different cell types and is affected mainly by the head groups. Our results demonstrate that the modular design significantly simplifies the creation of fluorescent cholesteryl probes bearing distinct spectral, biophysical, and cellular targeting features. It is a valuable toolkit for imaging in live cells, measuring cellular membrane dynamics, and studying cholesterol-related processes.

**Supplementary Information:**

The online version contains supplementary material available at 10.1038/s41598-024-80958-2.

## Introduction

Cholesterol is an indispensable lipid for all animal eukaryotic cells. It is a major membrane component, behaves as a cofactor for signaling molecules, and acts as a precursor for steroid hormones^1,2^. Structurally, cholesterol influences membrane rigidity, permeability, and curvature. For membrane microdomains (a.k.a., lipid rafts), cholesterol is decisive to their biophysical property, which facilitates the localization and function of transmembrane signaling^3^. The intracellular cholesterol trafficking between organelles is mediated by both vesicular and non-vesicular lipid transport mechanisms^2,4,5^. Due to its involvement in numerous signaling pathways and cellular processes, cholesterol uptake, trafficking, and distribution are tightly regulated by complex mechanisms.

Fluorescence imaging of cellular cholesterol has been achieved using cholesterol sensors, intrinsically fluorescent cholesterol analogs, or fluorescently labeled cholesterol derivatives^6^. The commonly used fluorescent cholesterol sensors are filipin (intrinsically fluorescent polyene antibiotic that binds to non-esterified cholesterol)^7^ and fluorescently tagged perfringolysin O - particularly its domain 4 (D4)^8^. The application of filipin has a few drawbacks, including nonspecific binding to other lipids and permeabilization of cell membranes, which makes it unsuitable for live-cell applications^7^. Additionally, D4-like sensors suffer from their bulky size and potentially interfere with cholesterol behavior^9^. Recently, a novel cholesterol sensor named GRAM-W was developed by Koh et al.^10^. This biosensor contains the GRAM domain from GRAMD1b. However, it recognizes not only cholesterol but also anionic lipids, such as phosphatidylserine^11,12^.

Existing fluorescent cholesterol analogs can be divided into intrinsically fluorescent sterols or fluorophore-tagged sterols. Due to its complex interaction with various biomolecules, tagged fluorophores likely affect cholesterol’s functionality^6^. Consequently, different fluorescent cholesterol analogs are tailored for specific studies. Natural fluorescent sterols have three conjugated double bonds in the steroid ring system (e.g., dehydroergosterol, DHE, and cholestatrienol, CTL). Their disadvantage is lackluster fluorescence, including cyto-damaging UV, excitation, and low quantum yield^13^. DHE also has very limited environmental sensitivity. Although CTL is the closest fluorescent derivative of cholesterol, its fluorescence properties and photostability are similar to, if not worse than those of DHE^14^. Fluorescently labeled sterol probes have fluorophores attached to either the steroid backbone or are in the form of fluorescent cholesteryl conjugates (esters). The two most commonly used ones are nitrobenzoxadiazole (NBD) and boron dipyrromethene (BODIPY) core containing fluorophores^15–17^. NBD is smaller than the bulky BODIPY group, and unlike BODIPY, NBD is environment-sensitive^16^. On the other hand, BODIPY is a much brighter fluorophore. Modifying cholesterol’s hydrophobic tail with NBD resulted in the creation of 25-NBD-Chol and 22-NBD-Chol conjugates, which were used in many cellular studies^16,18–22^. The BODIPY-cholesterol (Bchol) was developed to better mimic cholesterol since BODIPY is electrically neutral (has no permanent dipole moment) and much brighter than NBD. It has been widely recognized and used in many cellular assays^23,24^. However, recent studies have shown that its intracellular trafficking significantly differs from that of cholesterol^24^.

The biological importance of cholesterol and the need of environment-sensitive analogs for cholesterol trafficking across cell membranes motivated us to develop new cholesterol probes. Here, we report a modular design for making fluorescent cholesterol analogs, a new class of cholesterol probes with spectral properties favorable for imaging and environmental sensitivity. Some of them are pH-sensitive and useful for tracking cholesterol trafficking among different organelles bearing different pH. By live-cell imaging, we showed that some of the new probes are better than Bchol in terms of photo properties and similarity to cholesterol.

## Materials and methods

### Reagents, solvents, and glassware

 All reagents and solvents (including anhydrous ones) were obtained from commercial sources (Fisher Scientific) and used without purification unless otherwise noted. Synthetic, purification, and analytical steps were performed at room temperature (20 °C) unless otherwise noted. All utilized glassware was oven-dried prior to. Nile red was purchased from ThermoFisher Scientific, LysoView 650 was purchased from Biotium, and BODIPY-cholesterol was purchased from Avanti Polar Lipids.

### Thin layer and column chromatography

 Thin-layer chromatography (TLC) was performed on aluminum backed plates coated with silica gel (60 Å pore diameter, 200 μm layer thickness, SiliCycle or EMD Millipore). All TLC plates contained a fluorescent indicator and were visualized by UV light (λ_Ex_ = 254–366 nm) or by staining with 10% H_2_SO_4_ in EtOH solution and subsequent burning using heat gun. All column chromatography purification procedures were performed on columns packed with silica (60 Å pore diameter, 40–60 μm particle size, SiliCycle).

### NMR and mass spectrometry

 NMR spectra were recorded on 400 MHz spectrometers at 22 °C in *d*-chloroform or *d*_4_-methanol. The signals in^1^H and^13^C NMR spectra were referenced to tetramethylsilane (TMS). The multiplicities of signals are reported as singlet (s), doublet (d), triplet (t), quartet (q), pentet (p), multiplet (m), broad (br) or a combination of these. High-resolution mass spectra (HRMS) were recorded on an Agilent 6230 Time-of-Flight (TOF) Spectrometer using Agilent 1200 series LC system (mobile phase - methanol with 0.1% formic acid) and ionized by ESI. Analysis was performed by University of Florida - Mass Spectrometry Research and Education Center through funding obtained from NIH S10 OD021758-01A1 and S10 OD030250-01A1 grants.

### Synthesis of CND probes

The synthesis scheme and methods for the synthesis of CND probes are provided in the supplementary material.

### Determination of absorbance spectra in organic solvents

 Stock solutions of the CND probes were diluted to 10 µM in chloroform and evaporated. The residual solids were re-dissolved in their respective solvents to obtain 10 µM solutions. The solvents used were acetone, chloroform, dichloromethane, acetonitrile, ethyl acetate, dimethyl sulfoxide, ethanol, hexanes, and methanol. The absorbance was scanned from 350 to 700 nm using the ThermoScientific Evolution 201 UV − VIS spectrophotometer.

### Determination of emission spectra in organic solvents

 Stock solutions of the CND probes were diluted to 1 µM in chloroform and evaporated. The residual solids were re-dissolved in their respective solvents to obtain 1µM solutions. The solvents used were acetone, chloroform, dichloromethane, acetonitrile, ethyl acetate, dimethyl sulfoxide, ethanol, hexanes, and methanol. The emission spectra were scanned with a fixed excitation wavelength of 405 nm using the PerkinElmer LS 55 fluorescence spectrometer. The emission spectra were collected from 420 to 700 nm.

### Determination of molar extinction coefficient in DMSO and CHCl_3_

 Stock solutions of the CND probes in respective solvents were diluted to 100 µM. For each data point, the solutions were diluted by a factor of 0.75 and the dilutions were performed in triplicates. The fixed absorbance values were obtained at the maxima wavelength of absorbance for each solvent. The absorbance values were obtained using the Thermo Scientific Evolution 201 UV − VIS spectrophotometer.

### Fluorescence dependence on pH in 1% OG solution

 1% of octyl-β-glucoside solutions (OG) were made at pH’s ranging from 5 to 10 using Sorenson’s phosphate buffer. The solution was gently shaken and allowed to settle for 30 min. The stock solution of the CND probes was diluted to 20 µM in DMSO. For each individual pH, 95 µL of OG solution was added to a 100µL well plate. All fluorescence measurements were recorded on a 96-well plate using a Spectramax Gemini EM plate reader (Molecular Devices). To each pH OG solution, 5 µL of dye was added to each well and aspirated 3 times. The plate was then incubated in darkness for 15 min. With a fixed excitation wavelength of 405 nm, the fluorescence was scanned in the visible region from the top of the plate with an excitation cut off at 455 nm. The fluorescence values for each dye were obtained at the maximum wavelength of emission and plotted against pH.

### Preparation and analysis of lipid phase partitioning of CND series in giant unilamellar vesicles

 Lipid stock solutions of DOPC (10 mM), brain sphingomyelin (10 mM), cholesterol (10 mM), DiD (100 µM), and CND dye (40 µM) were prepared in 9:1 CHCl_3_:MeOH solutions. To prepare the lipid mix (39.5% DOPC, 39.5% SM, 20.9% cholesterol, 0.1% DID, and 0.1% CND Dye), 34 µL of DOPC, 34 µL of SM, 18 µL of cholesterol, 10 µL of DiD, 22 µL of CND dye, were added to a single vial with a Hamilton syringe. An O-ring was placed at the center of the conductive side of the ITO glass and sealed with vacuum grease. 10 µL of the lipid mix was then added to the middle of the O-ring and air-dried. The ITO glass with the dried lipid mix was placed in a desiccator under vacuum for 1 h. For all aqueous solutions, Milli-Q grade water was filtered with a 0.22 μm pore size filter prior to use. After 1 h, a 230 µL solution of 50 mM sucrose was placed through a 0.22 μm pore-size filter onto the vacuum-dried lipid mix. The ITO glass was then placed onto the Vesicle Prep Pro (VPP) instrument with the conductive sides facing the sucrose/lipid mix solution. The automated VPP protocol was initiated, which began the initiation period of heating to 60° C for 10 min with the frequency set to 10 Hz and the voltage set to 0 V. After the initial 10 min, the voltage was increased to 3 V over a period of 5 min. This was followed by a 3-hour period of maintaining 10 Hz and 3 V for the electroformation of the vesicles to occur. After the 3-hour electroformation period a 20-minute period of decreasing the voltage to 0 V and the frequency to 5 Hz was performed. Finally, a 40-minute cooling period of the GUV solution to 23° C was performed to obtain the GUV solution. For imaging, the GUV solution was diluted in a 1:2 ratio (v: v) of GUV solution: sucrose. The imaging chamber (well) was prepared using a plastic (polypropylene) cap from HPLC injection vial that was greased and applied to a #1 glass coverslip. To that imaging chamber 20 µL of the diluted GUV solution was added. To make the vesicles suitable for imaging, 40 µL of Milli-Q filtered water was added to the well and incubated for 15 min. GUV images were obtained using a fluorescent laser confocal microscope Nikon A1R system using 20x objective. The excitation wavelength was set to 405 nm for CND detection with an emission detection range of 500 –550 nm. For DiD, the excitation was performed at 640 nm with an emission range from 663 to 738 nm. Individual vesicle images were cropped out one by one from the raw images. Vesicles that displayed clear phase separation as judged by the DiD channel were then processed using the GUV-AP plugin to quantify L_o_ partitioning^25^. The fraction of probes present in the L_o_ phase was calculated according to the equation:

$$\:\%{L}_{o}=\frac{F{L}_{o}}{F{L}_{o}+\:F{L}_{d}}\:x\:100$$

where ***FL***_*o*_ represents the average fluorescence intensity of the circular segment corresponding to the L_o_ phase, and ***FL***_*d*_ represents the average fluorescence intensity of the circular segment corresponding to the L_d_ phase.

The DiD channel was used for vesicle identification and stitching with a threshold coefficient of 0.12, a circle extension coefficient of 1.2, and an angle step of 3 degrees. A minimum of 10 vesicles were used for quantification of average L_o_ partitioning and statistical analysis.

### Molecular dynamics simulations

 All CND probe molecules were built in the CHARM-GUI server utilizing the input generator, ligand reader and modeler modules^26–28^. For the piperazine group, a positive charge was added to resemble the protonation state of piperazine at physiological pH. The CHARMM-compatible topology and parameter files of the ligands were created using CGenFF parametrization^29^. The membrane structures were built utilizing the membrane builder and the probe was positioned into the membrane to have the naphthalimide moiety at the membrane surface and cholesterol embedded in the hydrophobic portion of the membrane^30^. For 22NBD and 25NBD, the 3β-OH was placed at the membrane interface where the sterol and NBD fluorophore were embedded in the hydrophobic portion of the bilayer. The membrane lipids in each simulation consisted of a ratio of 1-palmitoyl-2-oleoyl-glycero-3-phosphocholine (POPC), cholesterol, or stearoyl sphingomyelin (SSM). Systems were constructed to have 0%, 5%, 25%, and 40%, moles of cholesterol with respect to the POPC lipids in the simulation. The sphingomyelin-containing system consisted of 25% sphingomyelin (SSM), 25% cholesterol, and 50% POPC. All lipids were placed symmetrically on both membrane leaflets. For all systems, physiological salt conditions were implemented with 0.15 M NaCl. For the systems that contained a positively charged piperazine, an additional chlorine ion was used in the simulation, and for systems containing CND10, an additional sodium ion was added to neutralize the system. All systems were simulated using CHARMM36m forcefield and GROMACS 2022.1 simulation package using GPU acceleration^31^. After the systems were built, the system underwent the steepest decent energy minimization with hydrogen bond constraints using the LINCS algorithm^32^. Following the energy minimization, the system underwent six periods of equilibration as recommended by the CHARMM-GUI protocol. For the equilibration periods, the temperature was coupled using the Berendsen thermostat at a temperature of 298.15 K for a time of 1 ps^33^. The pressure was coupled to 1 bar using the semi-isotropic Berendsen barostat for 5 ps and a compressibility of 4.5 × 10^− 5^ bar ^− 1^. Following equilibration, the production run was carried out for a total of 100 ns. The production run utilized the NPT ensemble using the Nose-Hoover thermostat. The electrostatics were defined by using the particle mesh Ewald method, with a cut-off of 1.2 nm.

For MD trajectory analysis, the last 80 ns were used. The trajectory files from the simulations were imported into the Visual Molecular Dynamics (VMD) software for analysis (version 1.9.4)^34^. For analysis of membrane properties such as tilt and thickness Membplugin was used^35^. For membrane thickness, all phosphorus atoms were selected as means for determining the bilayer thickness. Cholesterol tilt angle analysis was performed by selecting the C10 and C13 carbons on cholesterol for all sterols with the mass distributions of the phosphorus atoms representing the membrane normal.

To calculate the immersion in the membrane, the oxygen (C-3 bound oxygen) atom on the sterol and where applicable the hydroxyl oxygen was used as atom selection. For determination of immersion values the position in the Z-plane for all selected atoms and phosphorus atoms were obtained. To determine the boundaries of the membrane, the Z-position of phosphorus atoms were obtained for each leaflet for every frame. The membrane center was then calculated by adding the averaged values and dividing it by 2 to obtain a Z-value for the membrane center. The distance from the center of membrane (immersion) was calculated by subtracting the position of the selected atoms from the membrane center previously calculated. The hydrogen bonds were analyzed with VMD using the H-bond plugin. The number of H-bonds between the probe and membrane component (POPC, SM, water) was calculated using whole molecules as selection (e.g., POPC vs. CND) with a donor-acceptor cutoff distance of 3Å and the angle cutoff of 30 degrees. The bonds were analyzed across the last 80 ns of trajectory on every frame. For the cholesterol H-bond analysis, the total number of bonds formed with water, SM, and POPC was calculated and divided by the number of cholesterol molecules in the system to obtain average values, which were rounded to the nearest whole number.

### Animal protocols

 All animal protocols followed NIH guidelines and were approved by Florida Atlantic University’s Institutional Animal Care and Use Committee (IACUC). The study is reported in accordance with ARRIVE guidelines (https://arriveguidelines.org).

### NIH-3T3 cell culture

 NIH 3T3 fibroblasts were obtained from ATCC. One vial of cryopreserved NIH 3T3 cells was thawed in a 37 °C water bath and immediately plated in 75-mL culture flasks pre-coated with Matrigel (Corning). They were grown to confluence in 3T3 culture media (DMEM + GlutaMax with 5% fetal bovine serum). Confluent 3T3 cultures were washed with PBS and detached by 1% Trypsin/EDTA. After centrifugation, cell pellets were resuspended in the same media and plated onto quad-divided glass-bottom 35 mm culture dishes at a concentration of 1,000,000 cells/cm^2^. The resulting cultures were fed with the same media as before and were used within 2–5 days.

### Mouse cortical astrocyte cell culture

 Postnatal day 0–2 mouse pups were euthanized by cervical dislocation, and their cortices were dissected. 0.1% Trypsin/EDTA treatment followed by mechanical dissociation was used to separate cells. After centrifugation, cell pellets were resuspended in astrocyte culture media (DMEM + GlutaMax with 10% fetal bovine serum) and quickly plated in 75-mL culture flasks pre-coated with Matrigel. After 7–10 days in vitro, astrocytes outgrew other types of brain cells that were further removed by multiple washing with cold PBS and repeated shaking. Surviving astrocytes were detached by 1% Trypsin/EDTA and collected by centrifugation before cryopreservation in astrocyte media containing 9% glycerol. For experimental use, cryopreserved astrocytes were revived and plated in the same way as that of 3T3 cells, except for the use of astrocyte media.

### Live-cell fluorescence confocal imaging

 A Nikon A1R confocal microscope equipped with a 60× (N.A. 1.40) oil objective and excitation lasers with matching optical filters for CNDs (405 nm excitation) or Bchol (488 nm excitation), 496 nm long-pass dichroic filter, and 510/20 nm band-pass emission filter), Nile Red (540 nm excitation, 565 nm long-pass dichroic filter, and 600/40 nm band-pass emission filter), and LysoView 650 (640 nm excitation, 680 nm long-pass dichroic filter, and 710 nm long-pass emission filter). Nikon Elements program was used for image acquisition. Laser power, gain, and offset were chosen to maximize fluorescence dynamic range for every channel and minimize bleed-through of other fluorophores. And those settings were kept the same for the same types of imaging experiments. Cells were loaded with the desired probe to obtain a final dye concentration of 1 µM for 1 h and then incubated in dye-free media for desired periods of time until imaging. Before imaging, cells were loaded with Nile Red and LysoView (0.5 µM) for 1 h and then washed with dye-free media immediately before placing inside an Oko-Lab weather chamber which was mounted on the microscope stage. The chamber was pre-warmed to 37 °C and filled with 5% CO_2_ and 100% humidity. The 60× objective was also pre-warmed to 37 °C. For every sample, three fields of view were selected randomly, and images with a frame size of 1024 × 1024 pixels were acquired. For all z-stack acquisitions, the z-axis step size was 0.3 mm. For all time-lapse imaging, the frame interval was 10 s, and the duration was 3–5 min. Acquired images were then further analyzed using ImageJ/Fiji^36^. For pH imaging using CND3 and Transferrin-Alexa568, we loaded 1 µM CND3 and 50 µg/ml Transferrin-Alexa5 in the culture media and incubated them with astrocytes at 37 °C and 5% CO_2_ for 1 h. Cells were then washed with dye-free media and mounted on a microscope for imaging. We used six coverslips from two batches of astrocyte cultures and imaged one field of view per coverslip.

### Particle analysis

 For particle analysis, the fluorescence intensity across the Z-stack was analyzed, and a single frame was extracted and saved for further analysis. To generate ROIs for particle analysis, a trainable Weka segmentation was employed^37^. The Weka classifier was trained on a series of three noisy (low s/n) and three good (high s/n) from the pool of acquired images. Classified images were converted into masks, and ROIs were generated. Upon ROI generation, particle analysis was performed in Fiji/ImageJ with object detection between 0.1 μm^2^-10 μm^2^. For each ROI, mean pixel intensity and area were obtained. For obtaining the diameter, the area was used, and the particles were assumed to be perfect circles. The particle size used for analysis was in the range of 0–3 μm. Data was visualized and analyzed using the R programming language and RStudio package. The Wilcoxon test was employed for statistical significance testing. For CND3 and Transferrin-Alexa568 images, we used the brightest CND3 image (i.e., with pH5.5) to generate masks for individual astrocytes by auto-thresholding. The same masks were used to register CND3 and Transferrin-Alexa568 signals acquired at the same fields of view. The average pixel intensity for every cell over time was calculated.

### Colocalization analysis

 In colocalization studies, while Pearson’s correlation coefficient (PCC) is useful for assessing the overall association between two probes in an image and is being reported frequently, it falls short in accurately measuring the fraction of one marker that colocalizes with another, a crucial aspect in cell biology analyses^38^. In contrast, the Manders’ colocalization coefficient (MCC) directly measures co-occurrence regardless of signal proportionality. However, to employ MCC effectively, it’s imperative to eliminate background noise from pixel intensities^38^. While subtracting a global threshold value is a common method, determining the appropriate threshold can be challenging. Costes et al. devised an efficient, robust, and reproducible approach to automatically identify the threshold value by analyzing the pixel values that yield a positive Pearson’s correlation coefficient (PCC)^39^. It iteratively measured PCC for different pixel intensity values until reaching values where PCC dropped to or below zero. The corresponding intensity values on the regression line were then utilized as thresholds for each channel. Only pixels surpassing both thresholds were considered colocalized. Consequently, the Costes’ method eliminates human bias. To determine both PPC and MCC, we used the EzColocalization^40^ plugin available for ImageJ. Colocalization was performed between selected channels (e.g. CND vs. LysoView) within each Z-stack on a plane-by-plane basis in a batch mode, with Costes’ thresholding. PCC and MCC values were obtained from each slice in Z-stack. Data from 1 h to 24 h was then compared and plotted using GraphPad Prism (ver. 10). Statistical analysis was performed using the Wilcoxon matched-pairs signed rank test.

## Results

### Preparation of CND probes

 Naphthalimide-conjugated molecules have been used as DNA intercalating agents, RNA sensors, cancer therapeutics, organogels, organic light-emitting diodes, enzyme sensors, and cell imaging agents^63–67^, demonstrating their biological compatibility and cellular tolerance. Our recent paper has demonstrated that a 1,8-naphthalimide (ND) scaffold possesses excellent solvatochromic properties^68,69^ and can be used to design environment-sensitive lipid probes^43^. The ND is a push-pull type of fluorophore, exhibiting an intramolecular charge transfer fluorescence mechanism upon excitation.

**Fig. 1:**
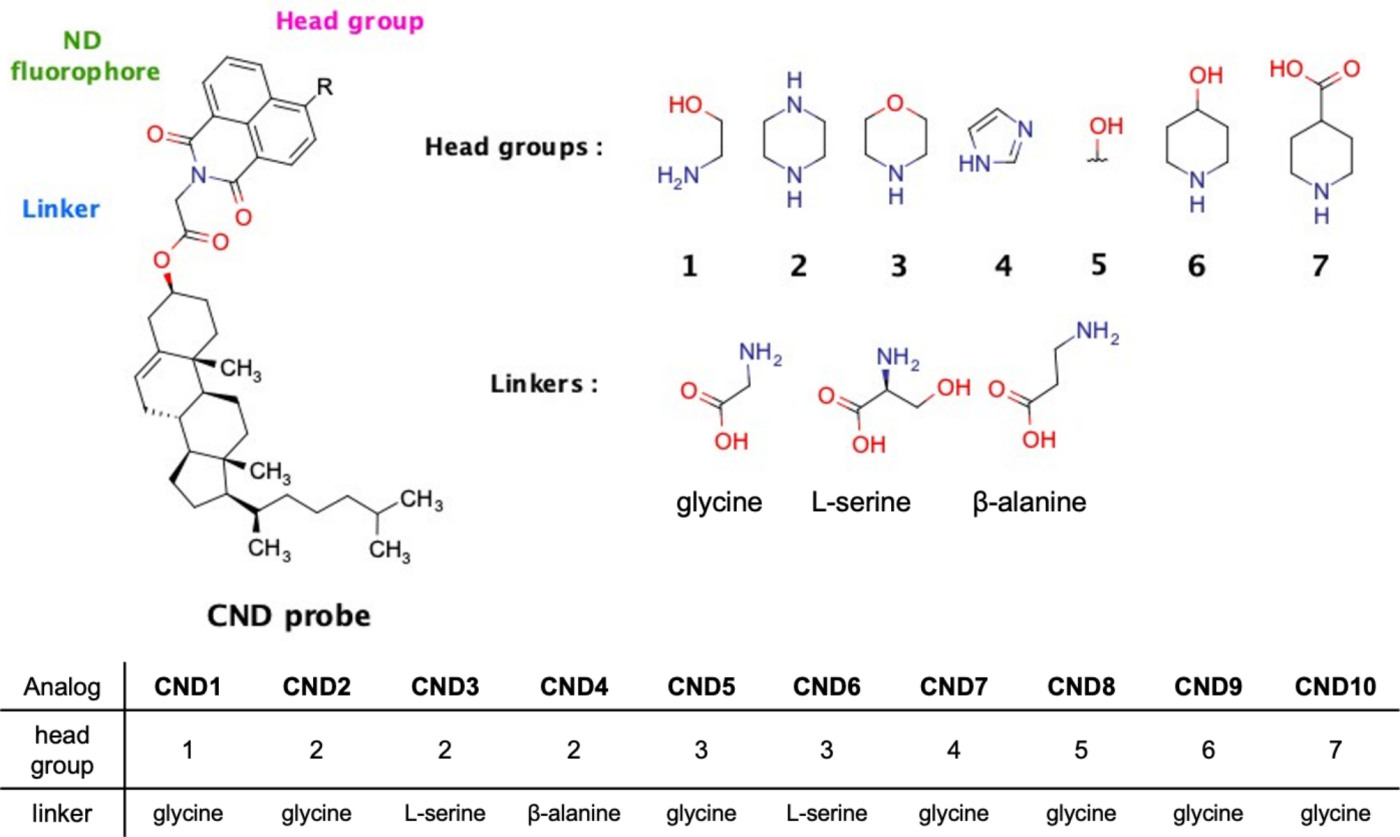
Structural modularity of CND probes. Variation of head groups and linkers used to make CND1-CND10.

All fluorescent cholesterol CND analogs can be prepared by a reaction of an amino acid (linker) with 4-bromo-1,8-naphthalic anhydride to form 4-bromo-1,8-naphthalimide, which then is used to esterify cholesterol (Scheme S1). Substitution of bromine at the C4 position renders a complete CND probe with unique photophysical properties. The order of the reaction sequence depends on the linker used. As linkers, we have employed glycine, L-serine, and β-alanine. Ethanolamine, piperazine, morpholine, imidazole, hydroxyl, 4-hydroxypiperidine, and 4-carboxypiperidine were used as head groups in various combinations to make a set of ten different CND probes (Fig. 1). The choice of linkers and head groups was supported by the molecular dynamics (MD) simulations, with which we analyzed the behavior of cholesterol group and its interactions in the membrane. The chemical synthesis of CND analogs was accomplished in three or four steps, depending on the types of linkers used.

### The influence of the head groups and the linkers on CNDs’ photophysical properties

 To establish the solvatochromic properties of CNDs, we determined the excitation and emission spectra of the CNDs in various solvents (Fig. S1 and Table S1). The absorbance maxima for the analogs with nitrogen-substituted aliphatic rings at the C4 position (CND2-6, 9, and 10) are proximal to 405 nm, which is a common laser wavelength for excitation in fluorescent microscopy. Most of their emission maxima also nicely fall into the commonly used green channel (i.e., 500–550 nm). The substitution at C4 with aliphatic ethanolamine results in obtaining analog (CND1) having red-shifted absorbance maxima (~ 420–440 nm). The solvatochromic properties of the analogs can be illustrated by the Lippert-Mataga plot (Fig. S2). To quantify solvatochromic properties, the maximum wavelengths of absorption and emission (λ_abs_/λ_em_) were obtained, and the Stokes’ shifts (∆ν) were plotted as a function of solvent orientation polarizability (∆f) to obtain Lippert-Mataga (LM) plot (Fig. S3)^41^. The deviation of positive, linear correlations suggests that there are fluorophore-fluorophore or fluorophore-solvent interactions influencing the intramolecular charge transfer (ICT) process in the ground and excited states. CND7 is the only analog that does not exhibit detectable correlation, likely due to its poor spectral profile. With its UV absorbance (342–346 nm) and near-zero fluorescence emission (440–505 nm), CND7 was found to be undesirable for further microscopy studies as a probe. CND8 is the only probe with a hydroxyl group present at the C4 position. It also exhibits unique solvatochromic properties due to its hydroxyl (phenolic) group interacting strongly with polar protic solvents (MeOH and EtOH) through hydrogen bonds (Fig. S1). For all CNDs, the molar extinction coefficients were determined in DMSO and chloroform (Table S2), ranging from 8000 to 16,000 M^− 1^ cm^− 1^, depending on the analog and solvent type used. This agrees with prior studies on substituted naphthalimides^42,43^.

**Fig. 2:**
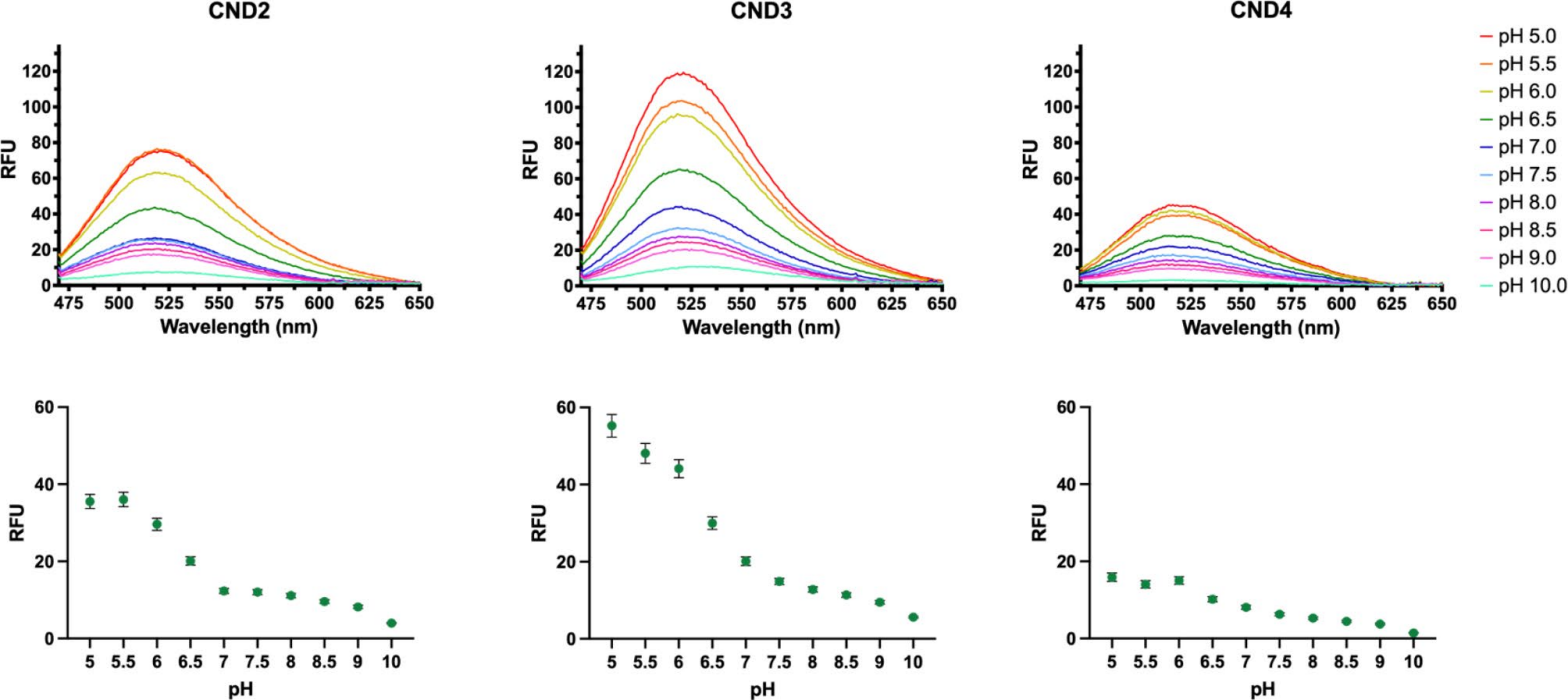
Emission spectra of CND2-CND4 in 1% octyl glucoside micellar solution in Sorensen’s buffer at various pH (λ_Ex_ = 405 nm) demonstrating pH sensitivity of these analogs (top row). The fluorescence in the 470–650 nm range was used to calculate area under the curve to determine the relative fluorescence change (mean ± SEM, bottom row). The fluorescence increases 2-3-fold with pH drop from 7 to 5.

Given the solvatochromic characteristics of naphthalimide scaffold, we speculated that CNDs possess environmental sensitivity towards lipid membranes^44^. To test that, we used a 1% octyl glucoside micellar solution to imitate the membrane environment. In comparison to their spectra in water, CNDs containing ethanolamine (i.e., CND1), piperazine (i.e., CND2/3/4), and hydroxyl group (i.e., CND8) exhibit substantial fluorescence increase (50–400-fold) in the micellar solution (Fig. S3). The protonation state of the piperazine head group can influence the emission of the ND scaffold, rendering piperazine-containing CNDs pH-sensitive^43^. In 1% OG micellar solutions, we subjected CND2/3/4 to Sorensen’s phosphate buffer with variable pH and observed an inverse correlation between their fluorescence and pH (Fig. 2). The protonated amino group of piperazine inhibits the photoinduced electron transfer process in the naphthalimide scaffold^45,46^. For CNDs containing morpholine group (i.e., CND5/6), the increase in fluorescence is unexpectedly moderate. Upon close inspection, we observed a suspension of particles in CND5/6 solutions, which were highly fluorescent. We postulate that their considerable fluorescence in water likely results from the phenomenon known as aggregation-induced emission (AIE)^47^, which was well documented in some naphthalimide-based probes^48^.

### Lipid partitioning of CND probes

**Fig. 3:**
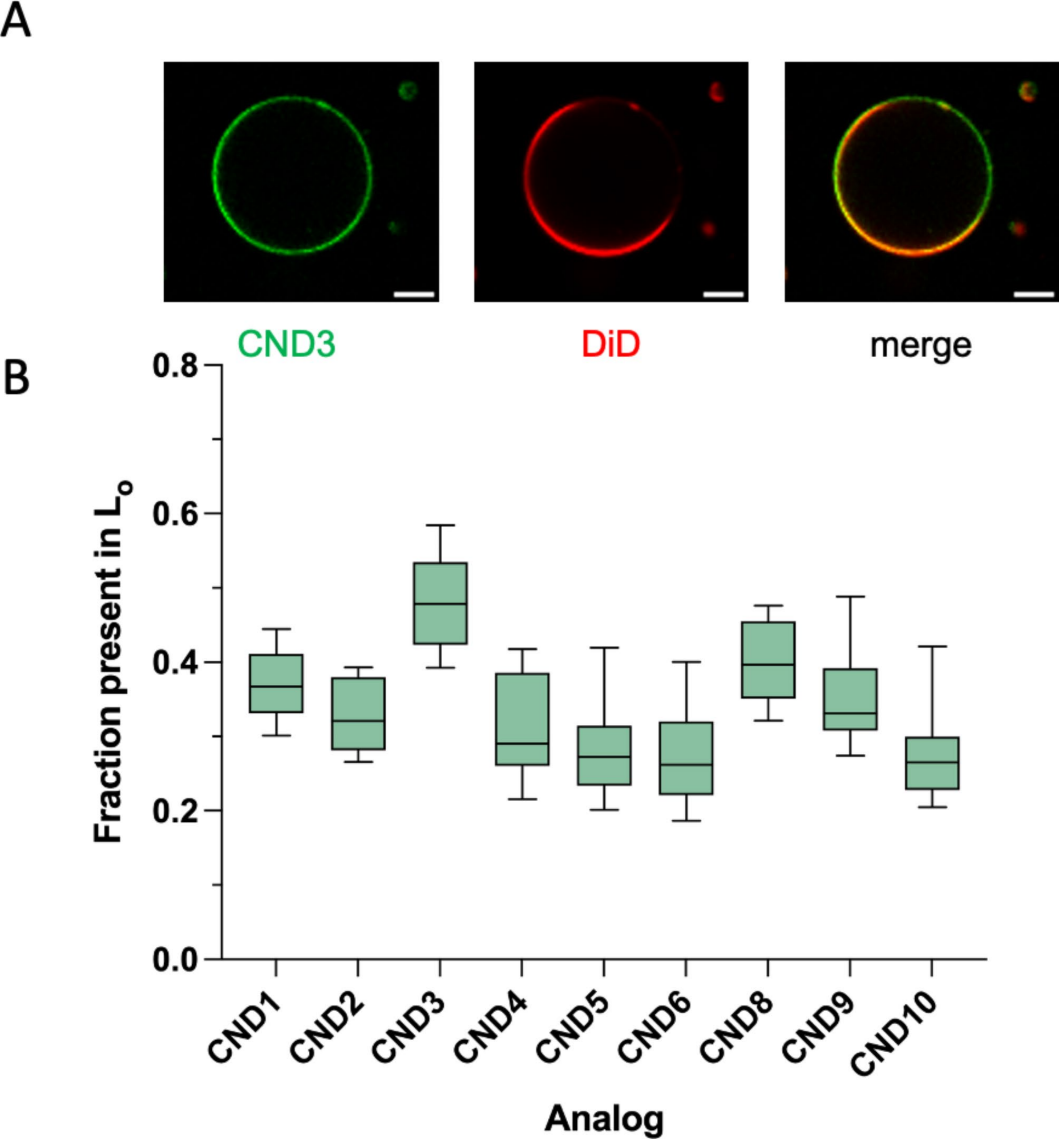
Partitioning of CNDs between L_d_/L_o_ phases in phase-separated GUVs. **A**, representative fluorescent confocal image of CND3 and Ld marker DiD. To determine the partitioning, the fluorescence intensity around the vesicle perimeter was measured using the GUV-AP plugin for ImageJ (see supplementary info). Scale bar, 10 μm. **B**, Fraction of probe partitioning into L_o_ phase for all tested CNDs.

 To determine the L_o_/L_d_ partitioning of CNDs, we made electroformed GUVs from a mixture of DOPC: SM: Chol (2:2:1) containing 0.1 mol% of L_d_ marker (DiD)^48^ and 0.1 mol% CNDs, following existing protocols^49,50^. As shown in Fig. 3A, CNDs’ L_o_ partitioning ranges from 30 to 50% with CND3 being the highest, which is comparable to Bchol^50^. Notably, CND3 possesses a hydroxyl group at its linker, whereas CND2 does not, demonstrating the importance of a hydroxyl group for L_o_ partitioning. Interestingly, the character of the head group also plays a significant role in the partitioning process. CND8, with a hydroxyl-containing head group, has the second-highest L_o_ partitioning. Similarly, the head groups of CND1 and CND9 also possess a hydroxyl group and thus exhibit relatively high L_o_ partitioning. On the other hand, analogs with neutral head groups (morpholine, CND5 and CND6) exhibited low L_o_ partitioning.

### Molecular dynamics simulations of CNDs in lipid bilayer

**Fig. 4:**
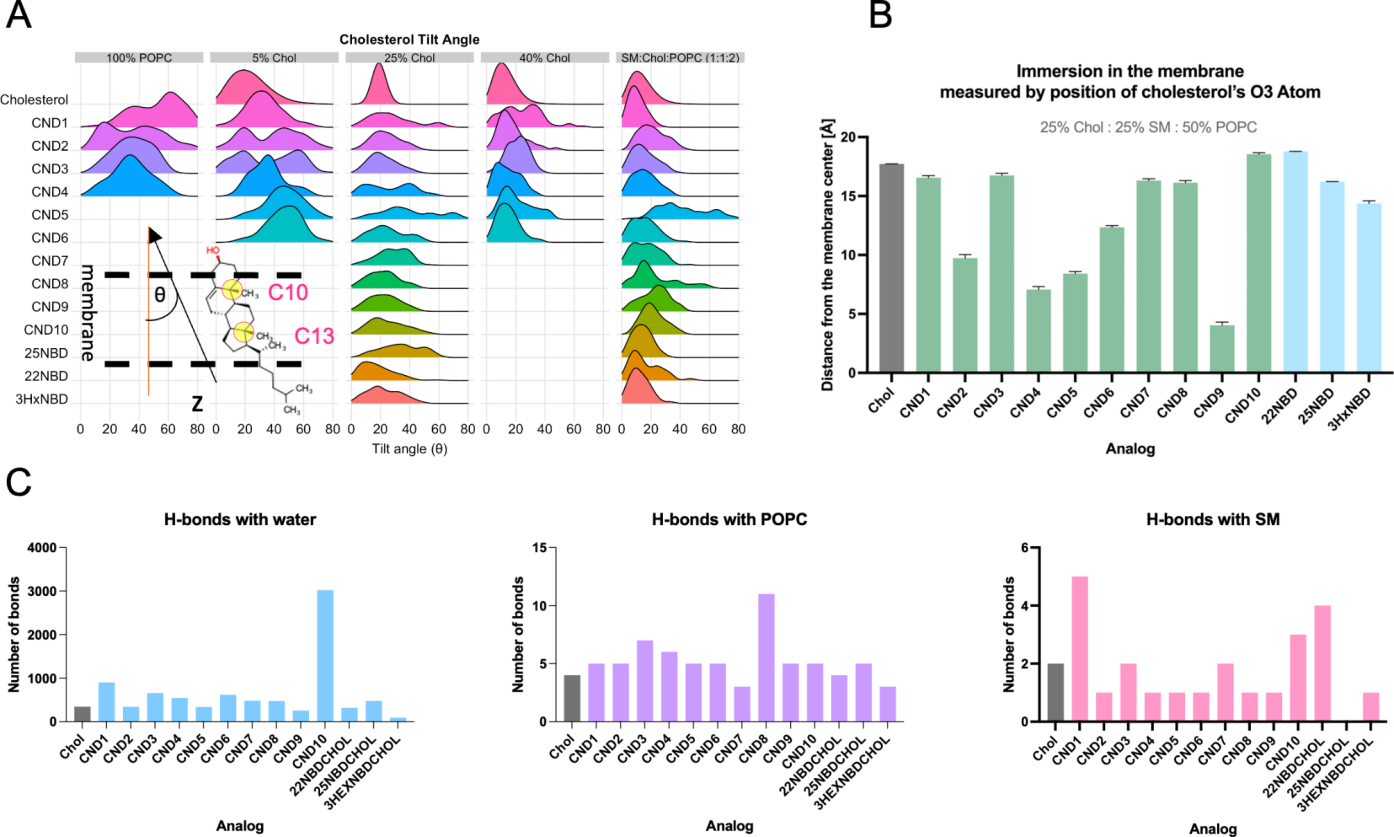
Characterization of probes using molecular dynamics simulations. **A**, analysis of the tilt angle of cholesterol. C10-C13 atoms were selected as selection (inset). **B**, analysis of the depth of probe immersion in the membrane in SM: Chol: POPC system. **C**, analysis of hydrogen bond patterns between cholesterol, probes and surrounding water, POPC, and sphingomyelin (SM).

 To evaluate the molecular interactions between CNDs and lipid membranes, we have conducted a set of molecular dynamics (MD) simulations. For comparison, we’ve included the 22-NBD-Chol, 25-NBD-Chol and 3-Hex-NBD-chol. To mimic different cell membranes, we used five different lipid compositions, four of which contain different amounts of cholesterol and POPC (Table S3). The fifth, SM: Chol: POPC (1:1:2), resembles the plasma membrane, especially microdomains^51,52^, the best. To efficiently evaluate their membrane integration, we focused on two key parameters specific to cholesterol, i.e., tilt angle (θ) and immersion depth^53^. We selected a vector defined by the positions of C10 and C13 atoms within the cholesterol moiety to determine θ relative to the membrane normal (Z). As shown in Fig. 4 and Table S4, the θ angle decreases as membrane cholesterol content and lipid packing increase, which agrees well with the literature^53^. SM (saturated lipids) also reduces θ as it increases lipid packing as well as membrane thickness^54^.

To measure the depth of CND immersion into the lipid membrane, we calculated the distance between the cholesterol O3 atom and the middle point of the lipid bilayer for both Chol: POPC, 1:3 (25% Chol) and the SM: Chol: POPC, 1:1:2 systems (Fig. S4, left column). The immersion depth of other hydroxyl groups within certain CND probes, either in linkers (CND3, CND6) or head groups (CND1, CND8, CND9), was also calculated (Fig. S4, right column). Since the presence of SM lipids in the system influences membrane behavior (ordering) and increases the type of in-membrane interactions available for the probes^52^, the SM: Chol: POPC system caused more difference than the simpler Chol: POPC (1:3) system (comparing the top vs. the bottom plots in Fig. S4). In the SM: Chol: POPC system, the CND1, CND3, and CND8 were immersed in the membrane very similarly to cholesterol. CND10 is the least immersed, even less than cholesterol (Fig. 4B), which is likely due to the electrostatic interactions of the carboxylate group with choline moiety from SM and POPC as well as the formation of hydrogen bonds with water (Fig. 4C right). It seems that the 4-carboxypiperidine head group position carboxylate is at the right height with respect to the choline group to maximize these interactions. In terms of hydrogen bond formation with SM, CND1 performed the best (Fig. 4C left). CND1 ethanolamine head group is water exposed and thus can form multiple hydrogen bonds with the phosphate groups from phospholipids as well as water, which prevents CND1 from further immersion into the lipid bilayer. The interactions of CND8 and its hydroxyl head group also follow a similar pattern, which is the most pronounced with POPC (Fig. 4C middle). On the contrary, the hydroxyl group of CND9 (4-hydroxypiperidine head group) forms the least number of hydrogen bonds with water likely due to the very hydrophobic character of this head group (Fig. 4C). It is also the reason that CND9 exhibits the deepest immersion among all analogs tested (Fig. 4B). The analogs containing piperazine head groups (i.e., CND2, 3 and 4) also show electrostatic interactions with the phosphate groups of phospholipids, although their immersion depth mostly depends on their linkers (Movie S1). CND4 with β-alanine linker shows the second deepest immersion in the membrane (Fig. 4B&C), as such linker enables for great flexibility and stretchability between naphthalimide moiety and the cholesterol residue. CND2’s glycine linker is slightly shorter and more constrained than the β-alanine one (Fig. 4B). The side chain hydroxyl group of serine linker (e.g., CND3) has a strong effect on its membrane immersion. It allows for additional hydrogen bond interactions with surrounding lipids and water and thus better imitating the behavior of native cholesterol’s hydroxyl group (Fig. 4B). Such effect is also evident when comparing CND5 to CND6, which have the same head group (i.e., morpholine) but glycine and serine as linker respectively. Notably, the morpholine head group also affects the membrane behavior of CNDs compared to piperazine moiety. Together, our simulation in SM: Chol: POPC membrane qualitatively agrees with that of GUV results in which CND3, CND8, and CND1 showed the most L_o_ partitioning (Fig. 3).

The immersion depth and cholesterol tilt angle of NBD containing cholesterol analogs (22NBD, 25NBD, and 3HxNBD) was comparable with that of CND1, CND3, and CND8, and the ester-containing 3HxNBD had the deepest immersion in the membrane (Fig. 4B). The cholesterol tilt angle in 3HxNBD was smaller than in 25NBD and on par with that of 22NBD (Fig. 4A). Such results are different for previously reported MD data for these analogs^55^. In the cited study, NBD probes were positioned somewhat sideways relative to the membrane normal. The difference between these results can be attributed to the difference in the type of forcefield used in the simulation and the initial orientation of the probes in the membrane. As mentioned in the recent review, published studies on the behavior of NBD-containing cholesterol probes in the membranes show some discrepancy^56^. Regarding L_o_ partitioning, CNDs, under similar conditions, perform better than 22- and 25NBD analogs and even better than the 3HxNBD^50^.

### Live-cell imaging of CNDs

 Using Bchol as a reference, we first studied the distribution of CNDs in vitro using cultured 3T3 fibroblast cells and mouse astrocytes. To mark lipid droplets and lysosomes with different fluorescence, we used Nile red^57^ and lysosomes (LysoView™ 650), respectively. Following 1-hour incubation with individual CNDs, cells were rinsed with dye-free media to remove unloaded dyes. Multi-color fluorescence confocal imaging was carried out immediately after the 1-hour incubation and again at 24 h post-incubation to assess the localization of CNDs. As expected, different CNDs exhibit different cellular staining patterns. As shown in Figs. S5 and S6, some showed poor cellular uptake (e.g., CND5) while others exhibited strong staining (e.g., CND1, CND2-4); some showed more diffusing pattern in cells (e.g., CND1) while others exhibited punctuated labeling (e.g., CND2-4); some distributed in all parts of cells (e.g., CND6, CND9,) while others concentrated near nuclei (e.g., CND3).

**Fig. 5:**
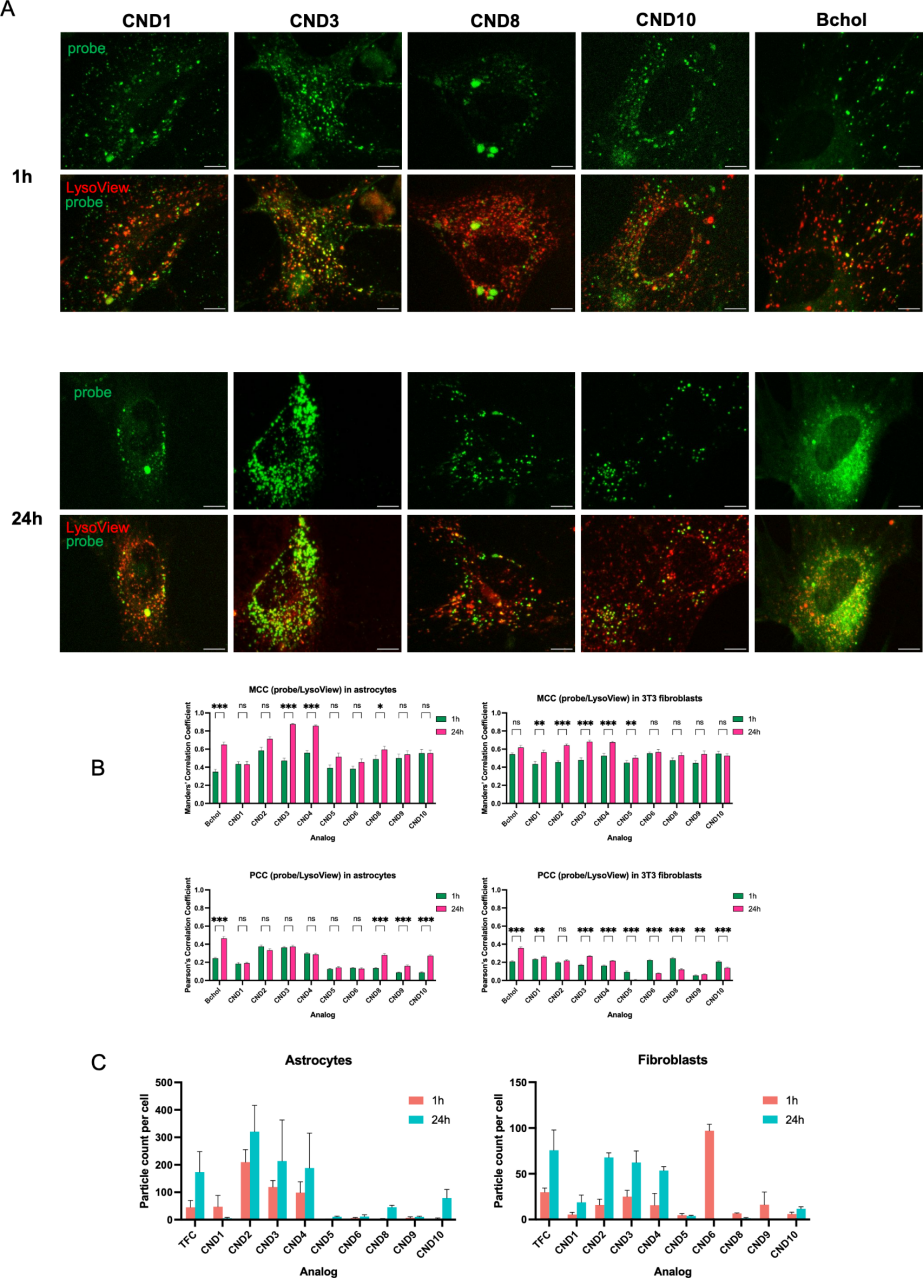
Live cell imaging of CND probes in cultured astrocytes. **A**, sample images for analogs that are neutral (CND1, 8), positively charged (CND3), and negatively charged (CND10) compared with Bchol (average Z-stacks). Overlay images show co-labeled LysoView (red) overlapping with selected CNDs better than Bchol. Scale bar, 10 μm. **B**, Manders’ (MCC) and Pearson’s (PCC) correlation coefficients between probe/LysoView at different time points and cell types. **C**, relative probe uptake and accumulation measured by particle count at different time points in astrocytes and 3T3 fibroblasts. Significance was calculated using a Wilcoxon matched-pairs signed rank. Differences were considered statistically significant at *P* ≤ 0.05 (*), *P* ≤ 0.01 (**), and *P* ≤ 0.001 (***); ns – not significant.

To generate masks for fluorescent puncta quantification, we used a machine learning approach implemented in Weka trainable segmentation (Fig. S7)^37^. We quantified the accumulation of probes by counting the fluorescent puncta per cell across both cell types and both time points (Fig. 5C). Notable patterns emerged. In astrocytes, CNDs with piperazine head groups (CND2, CND3, and CND4) showed a more significant number of fluorescent puncta in cells at both 1 and 24 h (Fig. 5C, S7), indicating that the head group may be the primary factor influencing their uptake and accumulation. In 3T3 cells, a similar pattern was observed. Notably, Bchol and CND6 were taken up more efficiently in 3T3 cells than in astrocytes.

When examining analogs with morpholine head groups, we noticed that the linker also influences the probe uptake. In both cell types, CND6, featuring a serine linker, showed significantly more uptake and intracellular accumulation (Fig. 5C and S6, S7). Interestingly, in 3T3 cells, CND6 displayed a high number of fluorescent puncta at 1 h but decreased dramatically at 24 h (Fig. 5 and S7). This suggests higher efflux or degradation of CND6 by fibroblast cells. Similar but less pronounced patterns were observed in astrocytes. CND5, with a glycine linker, exhibited the least uptake (Fig. 5C and S6, S7), likely due to its overall low solubility and propensity to aggregate in an aqueous solution.

Neutral CNDs with hydroxyl-containing head groups (CND1, 8, and 9) generally had fewer fluorescent puncta compared to those with piperazine head groups, CND2-CND4 (Fig. S7). However, CND1, CND8, and CND9 shared a similar uptake behavior as CND6 in 3T3 cells. In astrocytes, the patterns were the opposite, with CND8 and 9 showing more accumulation and CND1 exhibiting less. CND10, with a negatively charged carboxylate head group, exhibited a comparable distribution pattern in both cell types, accumulation after 24 h, but the degree of that accumulation was more pronounced in astrocytes.

Analyzing cell-wide probe distribution in astrocytes reveals distinct results (Fig. S5 and S7). For CND2-4, the fluorescent puncta were scattered across the cell at 1 h and became more perinuclear at 24 h. CND8, 9, and 10 exhibited a different pattern where the puncta not only became more defined after 24 h but also showed reduced fluorescence throughout the cells. CND1 presented yet another distinct pattern, i.e., puncta were well-defined at 1 h and became less intense (more diffused throughout the cells) by 24 h. Bchol, also accumulated as fluorescent puncta, was more dispersed and remained so even 24 h after loading (Fig. S5).

Next, we measured the size of the fluorescent puncta. While the majority of them were approximately 1 μm in diameter, there was a significant variation in size, as depicted in Fig. S9. In 3T3 cells, the puncta sizes were generally large for all CNDs except those with free hydroxyl head groups (CND6, 8, and 9). In astrocytes, however, the largest increase in puncta size was seen with CNDs that have piperazine head groups (CND2-4). This size increase was also noted for hydroxyl-containing head group analogs (CND8 and 9) and the negatively charged CND10. We further compared the accumulation of probes within the fluorescent puncta by assessing the average fluorescence intensity within individual particles (Fig. S10). As a reference, Bchol exhibited high average intensity in both cell types, slightly more in 3T3 cells. The fluorescent puncta of CNDs with positively charged head groups (CND2-4) exhibited a strikingly higher average intensity.

To determine the nature of those fluorescence puncta, we performed co-labeling with LysoView (a commercially available marker selective for lysosomes) and Nile red (typically used to label lipid droplets)^57^. By comparing co-staining between 1-hr and 24-hour time points, Bchol shows accumulation in lysosomes and lipid droplets that increases over time in both cell types (Fig. 5 & S8). This observation aligns with existing literature in which Bchol was found in lipid droplets inside CHO and HeLa cells and this accumulation was attributed to its BODIPY group^6,24,58^. Bchol accumulated in lysosomes after prolonged incubation was also reported previously^59^. Like Bchol, we also observed CNDs’ co-localization with LysoView and Nile red, which increased over time as well. Notably, the degrees of co-localizations at different time points vary among different CNDs (Fig. 5). Although we cannot exclude the possibility of nonspecific labeling, particularly by Nile red^60^, it is likely that CNDs, just like Bchol enter both lysosomes and lipid droplets over time. It is also possible that some lipid droplets and lysosomes are spatially too close to be distinguished by fluorescence imaging.

**Fig. 6:**
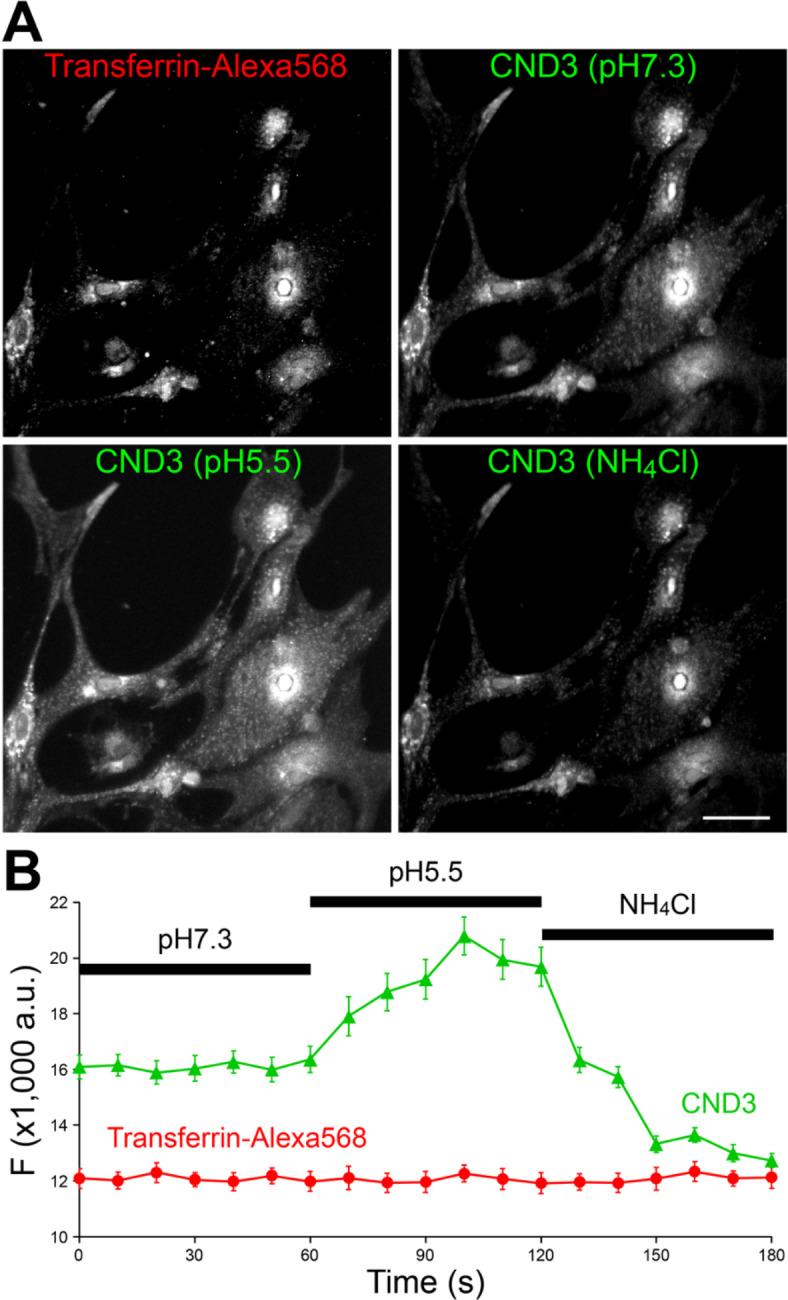
CND3 fluorescence changes during pH manipulation in astrocytes. **A**, sample images of CND3 co-loaded with Transferrin-Alexa568 conjugate in astrocytes 1 h after incubation and washing. When astrocytes were perfused with normal (pH 7.3), acidic (pH 5.5), and NH_4_Cl-containing Tyrode’s solutions, CND3 fluorescence changes were observed. Scale bar, 20 μm. **B**, average fluorescence change of CND3 and Transferrin-Alexa568 in astrocytes during 1-minute perfusion of the three solutions. Error bars are standard errors of means.

CNDs with piperazine head groups (i.e., CND2-4) exhibit pH sensitivity, allowing them to report luminal pH in organelles like endosomes and lysosomes. To demonstrate this, we used CND3, which has the highest pH sensitivity (Fig. 2). In astrocytes, we co-loaded CND3 with human Transferrin conjugated to Alexa568, which is known to label endosomes^61^. After 1-hour incubation and washing, those astrocytes were subjected to live-cell imaging. As shown in Fig. 6A, there is a strong similarity between CND3 and Transferrin-Alexa568 signals. A weaker and more diffused staining of CND3 can be attributed to its localization on the plasma membrane. We perfused those astrocytes with three different Tyrode’s solutions, i.e., normal (pH 7.3), acidic (pH 5.5), and NH_4_Cl (50mM). While the first two primarily affect extracellular pH, the last neutralizes all luminally acidic organelles like endosomes and lysosomes^62^. As shown in Fig. 6, the application of acidic Tyrode’s solution caused a significant increase of CND3 fluorescence, more prominent in its diffusing labeling, i.e., surface CND3. When NH_4_Cl was applied, a significant decrease of CND3 fluorescence was observed, consistent with the neutralization of endosomes. In either case, the fluorescence of Transferrin-Alexa568 showed little change because it is pH-insensitive. Therefore, we conclude that pH-sensitive CNDs can serve as membrane-based pH reporters for surface and organelles’ lumen.

## Discussion

We have taken advantage of the unique ND structure and its fluorescent properties to create a series of cholesteryl probes (Fig. 1). The modular structure of CND probes allows us to build a toolbox of fluorescent cholesteryl probes with different membrane- and pH-sensitivities. As proof of concept, we have tested three different linkers and seven different head groups, demonstrating the generalization of this methodology for creating a large group of fluorescent reporters with a variety of properties. We found that the character of CND probes depends on the combination of linkers connecting cholesterol and ND scaffold and the head group installed on the fluorophore at the C4 position of the ND scaffold. Table 1 qualitatively summarizes the features of these probes.

**Table 1:** Summary of features of CND probes.

CND	Head group character	Linker	Aggregation tendency	pH sensitivity	% Lo partition	In-membrane immersion depth	Sterol tilt angle	Cellular uptake
1	Hydroxyl	Glycine	No	No	37	~ cholesterol	< cholesterol	+++
2	Charged (+)	Glycine	No	Yes	33	< cholesterol	> cholesterol	++++
3	Charged (+)	Serine	No	Yes	48	~ cholesterol	~ cholesterol	++++
4	Charged (+)	β-alanine	No	Yes	31	< cholesterol	~ cholesterol	++++
5	Neutral	Glycine	Yes	No	27	<< cholesterol	>> cholesterol	+
6	Neutral	Serine	Yes	No	27	< cholesterol	~ cholesterol	++
7	Charged (+)	Glycine	Yes	No	N/A	~ cholesterol	~ cholesterol	N/A
8	Hydroxyl	Glycine	No	No	40	~ cholesterol	> cholesterol	++
9	Hydroxyl	Glycine	Yes	No	35	<< cholesterol	> cholesterol	++
10	Charged (-)	Glycine	No	No	27	> cholesterol	> cholesterol	++

N/A – not assessed.

Resulting analogs can be categorized into three groups, depending on the nature of the head group: neutral (CND5, CND6), neutral but bearing a hydroxyl group (CND1, CND8, CND9), and charged (CND2-CND4, CND10). Each of them was assessed for its solvatochromic behavior in organic solvents and model membranes. The unique photophysical properties of CNDs make them more useful in live-cell fluorescence imaging than NBD- and BODIPY-conjugated cholesterol probes. Particularly, they have much larger Stokes shifts (~ 130 nm) than NBD-conjugated probes (~ 40 nm) and Bchol (~ 10 nm), which is particularly beneficial for co-imaging with other fluorescent probes.

Our results testify that the head group character has the greatest impact on the fluorescence of the 1,8-naphthalimide scaffold, photoproperties, and cellular labeling. The neutral CNDs, with morpholine head group (CND5, CND6), exhibit elevated aggregation in water and enhanced fluorescence of these aggregates due to the AIE effect. The limited solubility of the neutral analogs results in a lower cellular uptake compared to positively charged analogs. It also affects the probes’ partitioning in the lipid membrane, as shown by computer simulations and GUV tests. The employed all-atom molecular dynamics simulations provided a putative explanation of how CNDs behave in model membranes compared to cholesterol and helped us understand the role of linkers and head groups in the intramolecular interactions with other lipids in the membrane. To imitate the diverse lipid environments found in different types of cellular membranes, we have adopted five different membrane models in the simulations. As anticipated, the presence of cholesterol and SM lipids in the system influences membrane behavior (ordering) and increases the type of in-membrane interactions available for the probes^52^. We found that the SM: Chol: POPC membrane system showed better probe differentiation than the simpler Chol: POPC (1:3) system (comparing the top vs. the bottom plots in Fig. S4). That system also better reflected the experimental GUV data for probe partitioning into the L_o_ phase. In the experiments using GUVs, the neutral CNDs morpholine head group had the lowest L_o_ coefficient. The MD simulations revealed that the neutral analogs exhibit higher than average cholesterol tilt angles and deeper immersion in the membrane. In these analogs, the naphthalimide moiety tends to reside in the hydrophobic environment of lipid membranes.

The relatively hydrophobic nature and the resulting behavior of the neutral analogs containing the morpholine group can be improved by the incorporation of the hydroxyl group in the linker module. When glycine (CND5) is replaced with serine, the resulting compound (CND6) becomes more soluble. Moreover, such change has little effect on L_o_ partitioning but significantly improves membrane immersion depth. The difference between CND5 and CND6 is also evident in cell uptake results, especially in 3T3 fibroblasts. The CND6 produced a large number of fluorescent puncta 1 h after incubation. The significant clearance of CND6 at 24 h can be attributed to probe efflux (for example, by the ABC transporters^70^) or degradation of CND6.

The CND9 analog, which contains a relatively hydrophobic 4-hydroxypiperazine head group, highlights the importance of the hydroxyl moiety, a characteristic feature of the second category of neutral CND probes. Compared with CND5 and CND6, its L_o_ partitioning is better, likely due to the presence of an H-bonding hydroxyl group. Its relatively low fluorescence is compensated for by better uptake and membrane distribution at the 24-hour time point. Other analogs containing hydroxyl head groups, such as CND1 and CND8, follow the same trend. With its phenolic character, the hydroxyl group modifies the fluorescence properties of the ND scaffold in CND8. The same applies to CND1 and its ethanolamine head group. These probes do not exhibit AIE, in contrast to CND5 and CND6. The low membrane immersion depth demonstrates that the hydroxyl group participates in hydrogen bonding. CND8 and CND1 also partition well into the L_o_ phase, similar to CND3. These analogs were taken up by the cells and exhibited the same distribution pattern after 24 h, although CND1’s fluorescent puncta were relatively less visible in astrocytes, suggesting either more clearance or more diffused distribution. Clearly, as computer simulations show, the presence of a hydroxyl group increases the hydrogen bonding of these analogs, not only with water but also with other lipids in the membrane. CND1 formed the most hydrogen bonds with SM, while CND8 did so with POPC. These bonds are not only more frequent but also long-lasting.

Analogs containing polarizable piperazine head groups (CND2, CND3, CND4) represent a novel class of pH-sensitive fluorescent cholesteryl probes. The piperazine head group exhibits not only substantial AIE inhibition but also pH-dependent fluorescence change. Such pH sensitivity is very valuable since various organelles in the endosomal pathway contain cholesterol and exhibit different luminal pH (e.g., ~pH 6 in endosomes and ~ pH 4.5 in lysosomes)^71^. Hence, those pH-sensitive CNDs are useful for mapping out pH variance across different organelles in different types of cells, similar to the previously reported ND6 probe^72^. Moreover, such pH-sensitive cholesterol probes or lipid probes, in general, are very helpful in imaging the exo-/endocytosis of synaptic vesicles at nerve terminals^73^ and endosomal trafficking in general, bearing great promise in studying cholesterol-related disorders like Alzheimer’s disease^73–75^. The pH sensitivity of these analogs correlates with the exposure of the piperazine moiety to the aqueous environment, which also improves hydrogen bonding and electrostatic interactions with other phospholipids.

On the contrary, the negatively charged CND10 analog containing 4-carboxypiperazine is not pH-sensitive and differs significantly from the positively charged CNDs. Our simulation shows that the carboxylate moiety allows CND10 to immerse in the membrane less than cholesterol does while making the most contact with water among the analogs tested. However, the negatively charged carboxylate does not improve partitioning into the L_o_ phase. It also shows much lower cell uptake and fluorescence than the positively charged analogs.

Interestingly, the simulation data show that the immersion depth in the membrane among CND2-CND4 is influenced by the nature of the linker. The flexible β-alanine linker causes CND4 to immerse in the membrane the most. The glycine linker is more constrained than the β-alanine linker but less constrained than the serine linker. In contrast, the latter contains an additional hydroxyl group that facilitates not only pH sensitivity but also L_o_ partitioning. Our best analog, CND3, with its Lo partitioning of ~ 50%, is comparable to Bchol^50^. Different tests collectively demonstrate that, in general, the introduction of a hydroxyl group into the linker structure significantly enhances lipid partitioning performance. As previously mentioned, the improved properties are also evident in the case of CND5 and CND6. The piperazine-containing CND2-CND4 analogs clearly outperformed all other CNDs in terms of fluorescent signal intensity in live cell imaging. Not only are they readily taken up by the cells, but they also accumulate over time, showing no toxicity, as judged by cell morphology. The distinct staining patterns suggest that the nature of the head group is likely the primary factor determining probe uptake and accumulation.

We think that certain CNDs (i.e., CND1, CND2-4, CND6, CND8), by functional and structural resemblance, may be used to track intracellular sterol transportation *via* endosomal pathway^76^, possibly by interactions with caveolae^77,78^. In addition, those CNDs may also be useful in illustrating cholesterol exchange between lipid droplets and endosomes/lysosomes through vesicular and non-vesicular pathways.

Considering the central role of lysosomes and their participation in subcellular cholesterol distribution^79^, we deployed colocalization analysis for CNDs and LysoView (or Nile red). Similarly to Bchol, the colocalization of CNDs with both LysoView and Nile red increased over time, although the degree of co-localization at different time points varies among different CNDs (Fig. 5B and S8). It is highly likely that CNDs, just like Bchol, translocate to both lysosomes and lipid droplets at 1 and 24 h. This is consistent with the fact that most cholesterol taken up by cells ends up in lysosomes and lipid droplets^80^.

Given the nonspecific labeling by the Nile red^60^, and the crowded intracellular puncta, we cannot exclude the possibility that some CNDs are more exclusive for lysosomes or lipid droplets. It is also possible that lipid droplets laden with CNDs undergo autophagy, and end up in autophagosomes^81,82^, causing the overlapping of the two organelle markers^83^. Our analysis further revealed that pH-sensitive analogs (CND2-CND4) exhibited a 2-3-fold increase in fluorescence intensity after 24 h compared to other CNDs (Fig. S10). Such a substantial surge in fluorescence intensity could be due to their accumulation in autolysosomes, where the acidic environment inhibits the photoinduced electron transfer (PET) process in the naphthalimide group, resulting in amplified fluorescence. It seems rather unlikely that the increased intensity of CND2-CND4 is simply due to the accumulation of these analogs in lipid droplets or AIE because they are much less aggregable. In future studies, the subcellular distribution of CNDs, especially among endosomes, lysosomes, and lipid droplets, can be better elucidated by using highly specific organelle markers, as well as different loading methods (e.g., using methyl-β-cyclodextrin or LDL complexes)^59,84^.

## Conclusions

In summary, we have successfully developed a novel class of fluorescent cholesteryl probes, incorporating a 1,8-naphthalimide (ND) scaffold known for its excellent solvatochromic and fluorescence properties. These CND probes demonstrate remarkable versatility and functionality, enabling the detailed study of cholesterol dynamics within cellular membranes. The modular design of these probes, which allows for incorporating various head groups and linkers, provides a robust toolbox for investigating membrane environments and cholesterol trafficking. Our findings highlight the unique advantages of CND probes, including their large Stokes shifts and environment-sensitive fluorescence, which outperform traditional NBD- and BODIPY-conjugated cholesterol probes in several key aspects. Notably, the pH-sensitive CND analogs (CND2, CND3, and CND4) represent a significant advancement in the field of fluorescent cholesterol probes. Their ability to map pH changes across different organelles provides new ways for studying intracellular processes and cholesterol-related disorders. Additionally, the specific interactions and behaviors of CND1, CND6, and CND8 suggest their potential as tools for elucidating intracellular cholesterol trafficking and distribution. Our molecular dynamics simulations provide valuable insights into the interactions between CND probes and lipid membranes, further validating their utility in mimicking cholesterol behavior. The in vitro testing in 3T3 fibroblasts and mouse astrocytes confirms the efficacy and non-toxicity of these probes, paving the way for their application in live-cell imaging and beyond. The introduction of these environment-sensitive CND probes is anticipated to impact the study of membrane biology, lipid dynamics, and cellular targeting. We expect that these compounds will enhance our understanding of cholesterol-related processes and cellular membrane dynamics and find applications in drug delivery systems and other areas of biomedical research.

## Electronic supplementary material

Below is the link to the electronic supplementary material.

Supplementary Material 1

Supplementary Material 2. **Movie S1**. Visualization of molecular dynamics simulation trajectory of CND2 and CND3 in SM: Chol: POPC system (AVI).

Supplementary Material 3. **Movie S2**. Time-lapse of CND2 in 3T3 fibroblast at 24 h after incubation showing the movement of fluorescent puncta containing CND2 (green) and LysoView (red) (AVI).

## Data Availability

The datasets generated during the current study are available from the corresponding author upon reasonable request.
