# Supplementary material for: Development and characterization of fluorescent cholesteryl probes with enhanced solvatochromic and pH-sensitive properties for live-cell imaging
Source: Sci Rep. 2024 Dec 28;14:30777. doi: 10.1038/s41598-024-80958-2 (PMC11680847; doi:10.1038/s41598-024-80958-2)
Supplement: Supplementary file 1 — Supplementary Material 1 [file 41598_2024_80958_MOESM1_ESM.pdf]

Supplementary Information for:

Development and Characterization of Fluorescent Cholesteryl Probes with Enhanced Solvatochromic and pH-sensitive Properties for Live-Cell Imaging

Vicente Rubio[1], Nicholas McInchak[1], Genesis Fernandez[1], Dana Benavides[1], Diana Herrera[1], Catherine Jimenez[1], Haylee Mesa[2], Jonathan Meade[2], Qi Zhang[1,2], Ph.D., Maciej J. Stawikowski[1], Ph.D.

[1] *Department of Chemistry and Biochemistry, Charles E. Schmidt College of Science, Florida Atlantic University, 777 Glades Rd., Boca Raton, FL 33431, USA*
[2] *Florida Atlantic Stiles-Nicholson Brain Institute, Florida Atlantic University, 5353 Parkside Dr., Jupiter, FL 33458, USA*

Correspondence should be addressed to
Maciej J. Stawikowski (mstawikowski@fau.edu)

Table of Contents

PAGE

Supplementary figures and tables referenced in the main text

Photophysical characterization of compounds S3-S10
Molecular Dynamics related data S11-13
Cell images. Particle masks and particle quantification S14-S39

NMR and MS spectra of all intermediates and final compounds

NMR spectra S55
MS spectra S72

Movie S1. Visualization of molecular dynamics simulation trajectory of CND2 and CND3 in SM:Chol:POPC system. (AVI)

Movie S2. Time-lapse of CND2 in 3T3 fibroblast at 24h after incubation showing the movement of fluorescent puncta containing CND2 (green) and LysoView (red). (AVI)

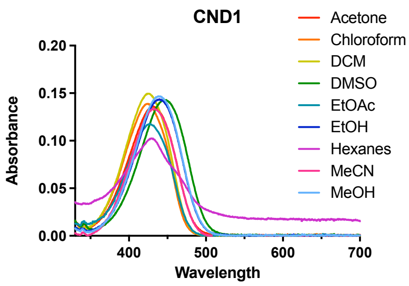
CND1
Absorbance
0.20
0.15
0.10
0.05
0.00
400
500
600
700
Wavelength
Acetone
Chloroform
DCM
DMSO
EtOAc
EtOH
Hexanes
MeCN
MeOH

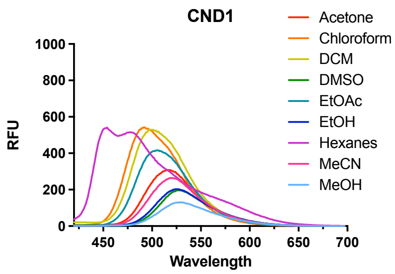
CND1
RFU
1000
800
600
400
200
0
450
500
550
600
650
700
Wavelength
Acetone
Chloroform
DCM
DMSO
EtOAc
EtOH
Hexanes
MeCN
MeOH

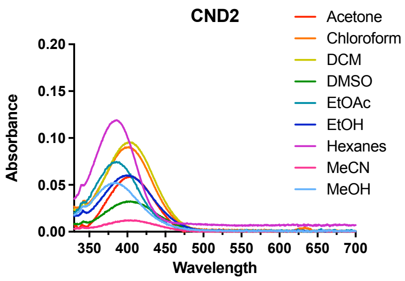
CND2
Absorbance
0.20
0.15
0.10
0.05
0.00
350
400
450
500
550
600
650
700
Wavelength
Acetone
Chloroform
DCM
DMSO
EtOAc
EtOH
Hexanes
MeCN
MeOH

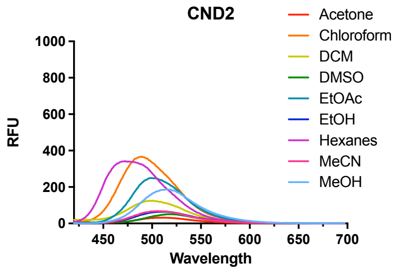
CND2
RFU
1000
800
600
400
200
0
450
500
550
600
650
700
Wavelength
Acetone
Chloroform
DCM
DMSO
EtOAc
EtOH
Hexanes
MeCN
MeOH

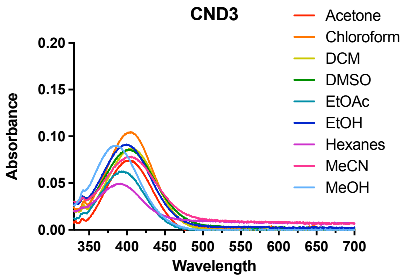
CND3
Absorbance
0.20
0.15
0.10
0.05
0.00
350
400
450
500
550
600
650
700
Wavelength
Acetone
Chloroform
DCM
DMSO
EtOAc
EtOH
Hexanes
MeCN
MeOH

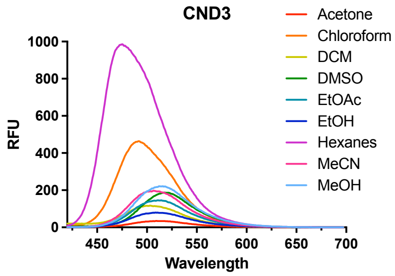
CND3
RFU
1000
800
600
400
200
0
450
500
550
600
650
700
Wavelength
Acetone
Chloroform
DCM
DMSO
EtOAc
EtOH
Hexanes
MeCN
MeOH

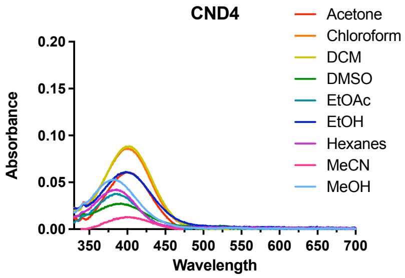
CND4
Absorbance
0.20
0.15
0.10
0.05
0.00
350
400
450
500
550
600
650
700
Wavelength
Acetone
Chloroform
DCM
DMSO
EtOAc
EtOH
Hexanes
MeCN
MeOH

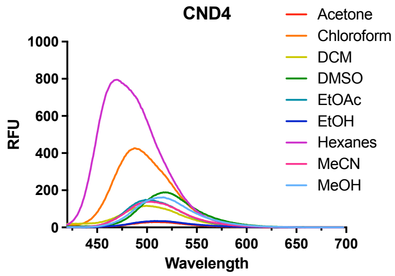
CND4
RFU
1000
800
600
400
200
0
450
500
550
600
650
700
Wavelength
Acetone
Chloroform
DCM
DMSO
EtOAc
EtOH
Hexanes
MeCN
MeOH

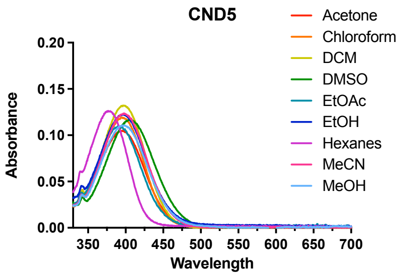
CND5
Absorbance
0.20
0.15
0.10
0.05
0.00
350
400
450
500
550
600
650
700
Wavelength
Acetone
Chloroform
DCM
DMSO
EtOAc
EtOH
Hexanes
MeCN
MeOH

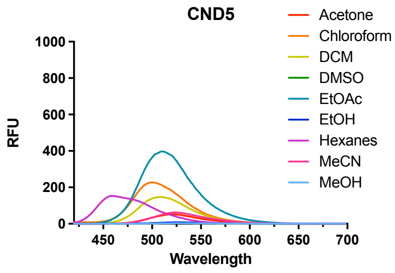
CND5
RFU
1000
800
600
400
200
0
450
500
550
600
650
700
Wavelength
Acetone
Chloroform
DCM
DMSO
EtOAc
EtOH
Hexanes
MeCN
MeOH

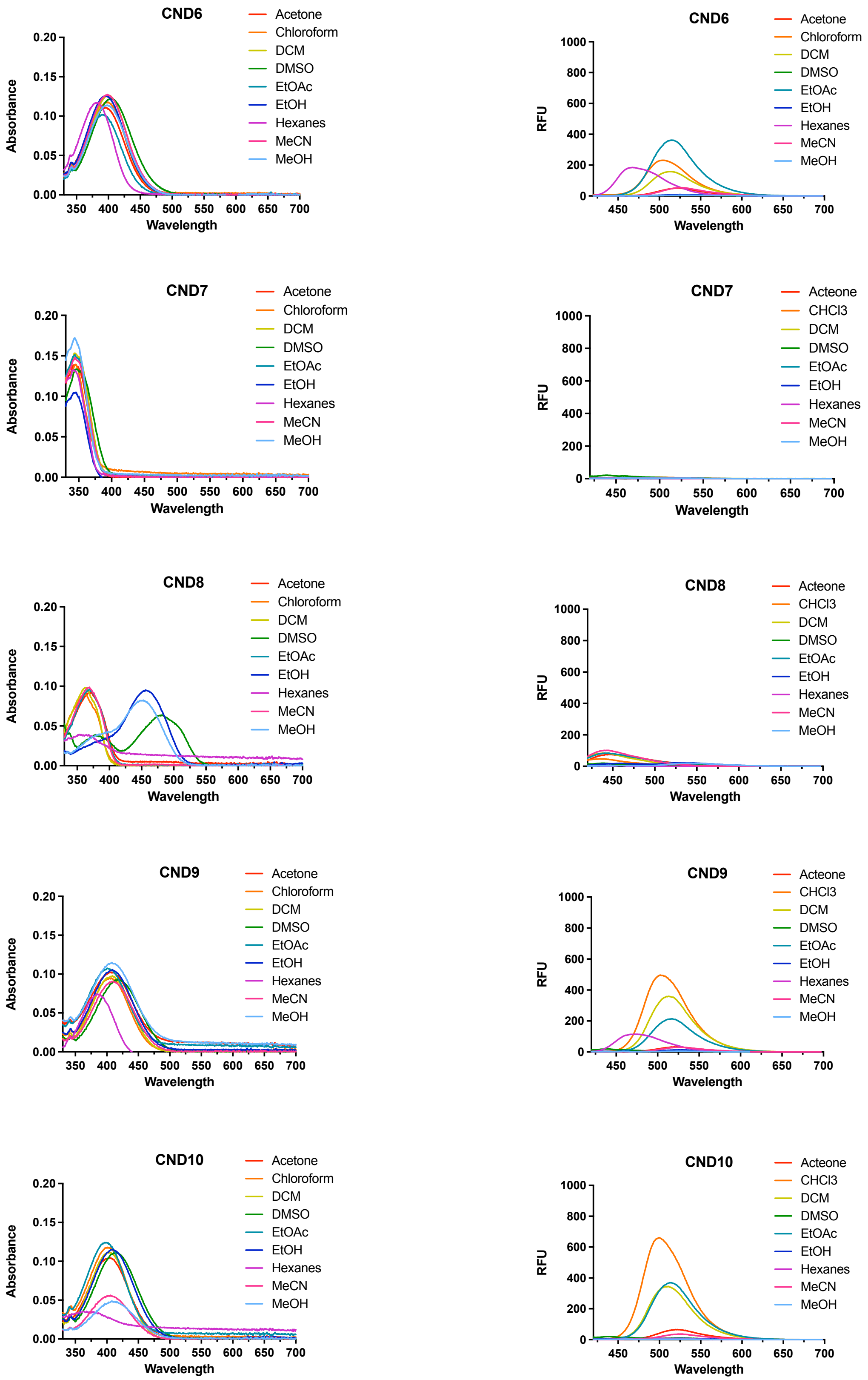

Fig. S1. The absorbance and emission spectra of CND analogs in various solvents.

Table S1. Absorbance (abs), emission (em) maxima, and Stokes' shift (Δλ) for all CND analogs. Values are provided in nanometers and wavenumbers. Values not obtained are denoted as n/a.

CND1						
Solvent	λ abs (nm)	λ em (nm)	Δλ (nm)	ν abs (cm^{-1})	ν em (cm^{-1})	Δν (cm^{-1})
Acetone	431	519	88	23202	19268	3934
Chloroform	425	492	67	23529	20325	3204
DCM	425	499	74	23529	20040	3489
MeCN	431	519	88	23202	19268	3934
DMSO	446	528	82	22422	18939	3482
EtOH	440	525	85	22727	19048	3680
Hexanes	429	454, 478	25, 49	23310	22026, 20920	1284, 2390
MeOH	440	530	90	22727	18868	3859
EtOAc	427	506	79	23419	19762	3656

CND2						
Solvent	λ abs (nm)	λ em (nm)	Δλ (nm)	ν abs (cm^{-1})	ν em (cm^{-1})	Δν (cm^{-1})
Acetone	403	512	109	24814	19531	5283
Chloroform	402	490	88	24876	20408	4467
DCM	402	501	99	24876	19960	4916
MeCN	406	505	99	24631	19802	4829
DMSO	403	519	116	24814	19268	5546
EtOH	400	511	111	25000	19569	5431
Hexanes	386	472	86	25907	21186	4720
MeOH	386	515	129	25907	19417	6489
EtOAc	386	499	113	25906	20040	5866

CND3						
Solvent	λ abs (nm)	λ em (nm)	Δλ (nm)	ν abs (cm^{-1})	ν em (cm^{-1})	Δν (cm^{-1})
Acetone	401	510	109	24938	19608	5330
Chloroform	401	492	91	24938	20325	4612
DCM	406	503	97	24631	19881	4750
MeCN	404	507	103	24752	19724	5029
DMSO	403	518	115	24814	19305	5509
EtOH	399	509	110	25063	19646	5416
Hexanes	392	475	83	25510	21053	4458
MeOH	386	513	127	25907	19493	6414
EtOAc	395	511	116	25316	19569	5746

CND4						
Solvent	λ abs (nm)	λ em (nm)	Δλ (nm)	ν abs (cm^{-1})	ν em (cm^{-1})	Δν (cm^{-1})
Acetone	399	503	104	25063	19881	5182
Chloroform	401	488	87	24938	20492	4446
DCM	400	499	99	25000	20040	4960
MeCN	399	504	105	25063	19841	5221
DMSO	391	517	126	25575	19342	6233
EtOH	398	508	110	25126	19685	5441
Hexanes	385	470	85	25974	21053	4921
MeOH	381	517	136	26247	19342	6904
EtOAc	383	499	116	26109	20040	6069

CND5						
Solvent	λ abs (nm)	λ em (nm)	Δλ (nm)	ν abs (cm^{-1})	ν em (cm^{-1})	Δν (cm^{-1})
Acetone	396	520	124	25253	19231	6022
Chloroform	397	500	103	25189	20000	5189
DCM	397	509	112	25189	19646	5543
MeCN	398	524	126	25126	19084	6042
DMSO	405	528	123	24691	18939	5752
EtOH	397	528	131	25189	18939	6250
Hexanes	378	459	81	26455	21786	4669
MeOH	395	530	135	25316	18868	6449
EtOAc	390	510	120	25641.03	19607	6033

CND6						
Solvent	λ abs (nm)	λ em (nm)	Δλ (nm)	ν abs (cm^{-1})	ν em (cm^{-1})	Δν (cm^{-1})
Acetone	396	524	128	25253	19084	6169
Chloroform	398	505	107	25126	19802	5324
DCM	401	513	112	24938	19493	5444
MeCN	399	525	126	25063	19048	6015
DMSO	405	528	123	24691	18939	5752
EtOH	397	525	128	25189	19048	6141
Hexanes	384	467	83	26042	21413	4628
MeOH	398	529	131	25126	18904	6222
EtOAc	389	515	126	25706	19417	6289

CND7						
Solvent	λ abs (nm)	λ em (nm)	Δλ (nm)	ν abs (cm^{-1})	ν em (cm^{-1})	Δν (cm^{-1})
Acetone	345	460	115	28986	21739	7246
Chloroform	345	501	156	28986	19960	9025
DCM	343	499	156	29155	20040	9114
MeCN	344	499	155	29070	20040	9030
DMSO	346	440	94	28902	22727	6174
EtOH	343	505	162	29155	19802	9353
Hexanes	342	459	117	29240	21786	7453
MeOH	344	460	116	29070	21739	7331
EtOAc	344	492	148	29070	20325	8745

CND8						
Solvent	λ abs (nm)	λ em (nm)	Δλ (nm)	ν abs (cm^{-1})	ν em (cm^{-1})	Δν (cm^{-1})
Acetone	368	448	80	27174	22321	4852
Chloroform	362	435	73	27624	22989	4636
DCM	363	438	75	27548	22831	4717
MeCN	369	443	74	27100	22573	4527
DMSO	481	n/a	n/a	20790	n/a	n/a
EtOH	456	533	77	21930	18762	3168
Hexanes	354	460	106	28249	21739	6509
MeOH	453	541	88	22075	18484	3591
EtOAc	370	440	70	27027	22727	4300

CND9						
Solvent	λ abs (nm)	λ em (nm)	Δλ (nm)	ν abs (cm^{-1})	ν em (cm^{-1})	Δν (cm^{-1})
Acetone	406	526	120	24631	19011	5619
Chloroform	407	503	96	24570	19881	4689
DCM	409	513	104	24450	19493	4957
MeCN	406	525	119	24631	19048	5583
DMSO	419	n/a	n/a	23866	n/a	n/a
EtOH	409	530	121	24450	18868	5582
Hexanes	387	472	85	25840	21186	4653
MeOH	408	528	120	24510	18939	5570
EtOAc	401	517	116	24938	19342	5595

CND10						
Solvent	λ abs (nm)	λ em (nm)	Δλ (nm)	ν abs (cm^{-1})	ν em (cm^{-1})	Δν (cm^{-1})
Acetone	399	522	123	25063	19157	5906
Chloroform	401	500	99	24938	20000	4938
DCM	401	511	110	24938	19569	5368
MeCN	402	523	121	24876	19120	5755
DMSO	416	n/a	n/a	24038	n/a	n/a
EtOH	409	527	118	24450	18975	5475
Hexanes	362	493	131	27624	20284	7340
MeOH	408	n/a	n/a	24510	n/a	n/a
EtOAc	397	512	115	25189	19531	5658

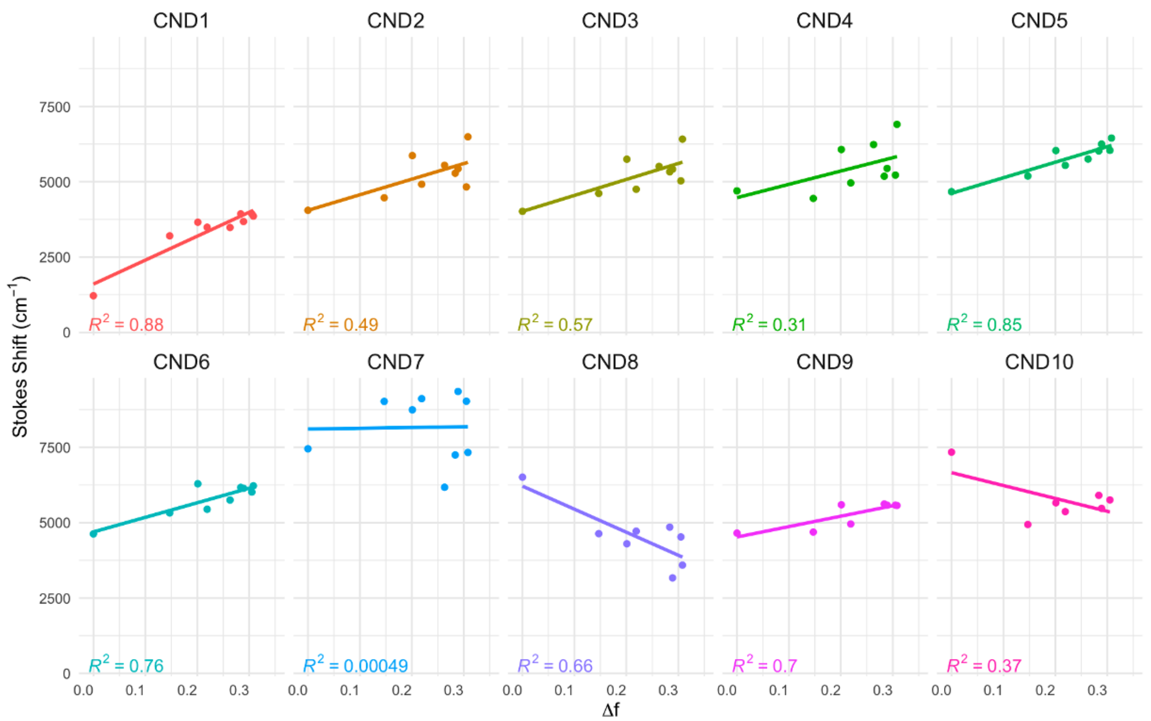

Fig. S2. Lippert-Mataga plot for CND series in selected organic solvents. Solvents tested in increasing order of orientation polarizability: hexanes, CHCl3, EtOAc, DCM, DMSO, acetone, EtOH, MeCN, MeOH. For CND8, CND9, and CND10, DMSO was omitted due to no detectable fluorescence. For CND10, MeOH was also omitted. Compounds were tested at 1 μM.

Table S2. Molar extinction coefficients of the CND series with a maximum wavelength of absorption for $CHCl_3$ and DMSO.

**Solvent**	**$CHCl_3$**		**DMSO**	
Analog	ε (M^{-1} cm^{-1})	λ_{abs} (nm)	ε (M^{-1} cm^{-1})	λ_{abs} (nm)
CND1	12850	425	16590	446
CND2	10170	402	9398	403
CND3	9455	401	10810	403
CND4	9228	401	8839	391
CND5	12000	397	11470	405
CND6	12510	398	11450	405
CND7	14830	345	13310	346
CND8	10240	362	12170	481
CND9	10420	407	11460	419
CND10	12840	401	10680	416

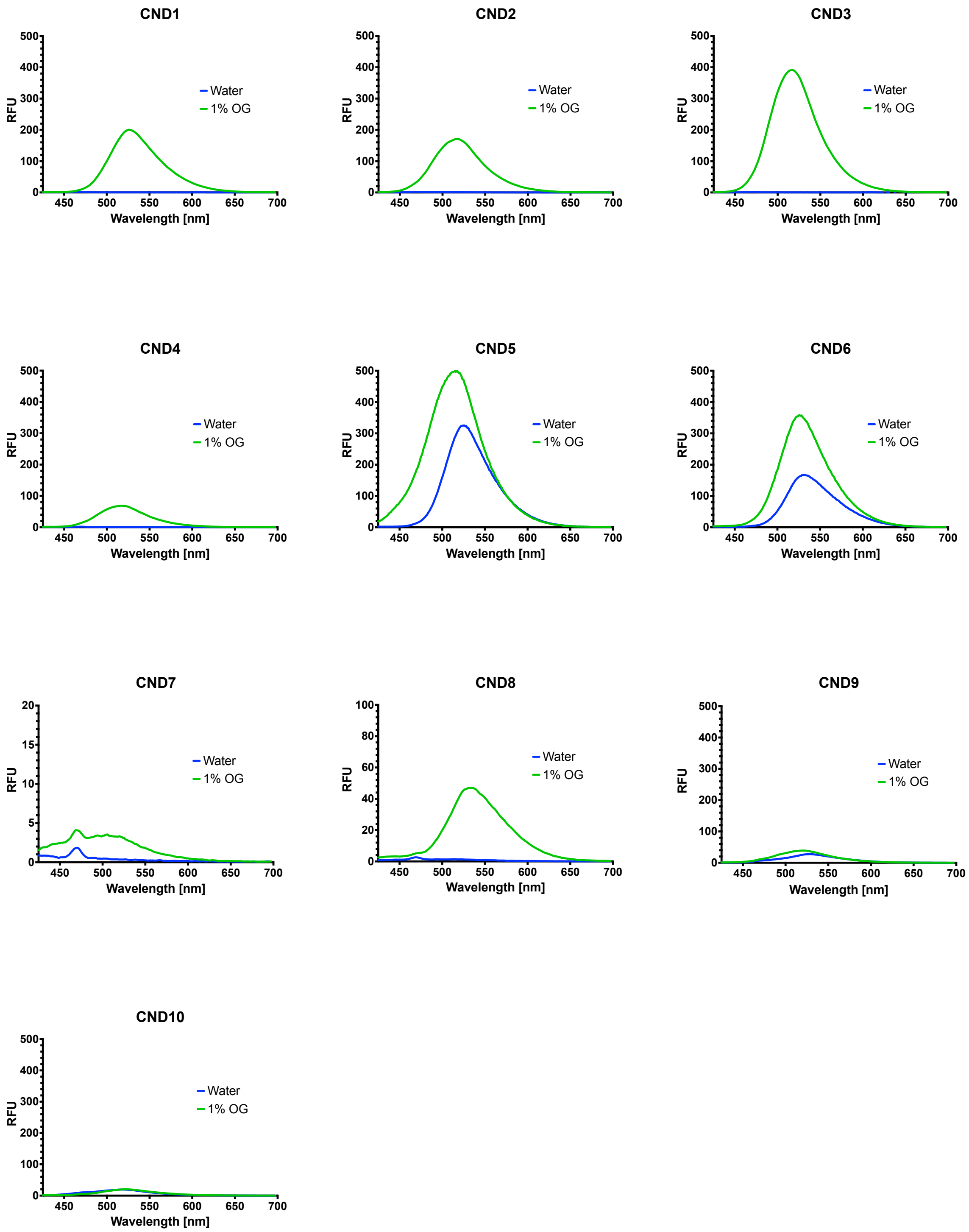

Fig. S3. Fluorescence emission spectra of CND analogs in 1% octyl glucoside micellar solution (green) and water (blue) showing membrane sensing and aggregation properties. Compounds were present at 1μM concentration.

Table S3. Lipid bilayer composition used for molecular dynamics simulations in this study.

System Name	POPC (mol %)	Chol (mol %)	SM (mol %)
100% POPC	100	0	0
5% Chol	95	5	0
25% Chol	75	25	0
40% Chol	60	40	0
SM:Chol:POPC (1:1:2)	50	25	25

Table S4. Cholesterol tilt angle analysis from molecular dynamics trajectories. Average sterol tilt angles with standard error of mean for cholesterol and all tested fluorescent cholesterol probes.

Analog	100% POPC	5% Chol	25% Chol	40% Chol	SM:Chol:POPC
Cholesterol	-	25 ± 0.23	19 ± 0.07	14 ± 0.06	15 ± 0.05
CND1	56 ± 2.10	33 ± 1.38	34 ± 2.58	25 ± 1.52	10 ± 0.60
CND2	47 ± 3.08	41 ± 2.38	24 ± 1.08	17 ± 1.06	21 ± 1.01
CND3	36 ± 1.50	36 ± 2.24	22 ± 1.03	21 ± 0.81	15 ± 0.93
CND4	37 ± 1.88	38 ± 2.33	37 ± 4.34	15 ± 0.92	16 ± 0.92
CND5	-	47 ± 1.53	40 ± 2.10	21 ± 1.21	42 ± 1.94
CND6	-	47 ± 1.29	25 ± 1.35	15 ± 0.83	15 ± 1.00
CND7	-	-	28 ± 1.15	-	17 ± 1.04
CND8	-	-	22 ± 1.15	-	23 ± 1.63
CND9	-	-	22 ± 1.14	-	23 ± 1.06
CND10	-	-	25 ± 1.35	-	21 ± 0.89
22NBD	-	-	17 ± 1.23	-	16 ± 1.15
25NBD	-	-	34 ± 1.51	-	14 ± 0.71
3HxNBD	-	-	22 ± 1.25	-	12 ± 0.74

Table S5. Average membrane thickness [Å] ± SEM derived from last 80 ns of MD simulation trajectory.

Analog	100% POPC	5% Chol	25% Chol	40% Chol	SM:Chol:POPC
CND1	39.8 ± 0.08	40.5 ± 0.06	44.8 ± 0.06	46.3 ± 0.05	47.2 ± 0.04
CND2	39.4 ± 0.08	40.5 ± 0.07	45.2 ± 0.06	46.2 ± 0.05	47.4 ± 0.07
CND3	39.1 ± 0.09	40.3 ± 0.05	44.7 ± 0.05	46.6 ± 0.04	47.1 ± 0.06
CND4	39.6 ± 0.07	40.6 ± 0.06	44.8 ± 0.05	46.8 ± 0.04	47.1 ± 0.04
CND5	-	40.8 ± 0.07	45.1 ± 0.05	46.3 ± 0.04	47.1 ± 0.05
CND6	-	40.5 ± 0.06	44.5 ± 0.06	46.5 ± 0.04	47.1 ± 0.04
CND7	-	-	45.0 ± 0.08	-	47.5 ± 0.05
CND8	-	-	44.8 ± 0.06	-	47.0 ± 0.04
CND9	-	-	44.4 ± 0.06	-	47.1 ± 0.08
CND10	-	-	45.0 ± 0.08	-	47.3 ± 0.05
22NBD	-	-	44.6 ± 0.06	-	48.0 ± 0.07
25NBD	-	-	44.7 ± 0.06	-	47.0 ± 0.07
3HxNBD	-	-	44.9 ± 0.05	-	47.0 ± 0.05

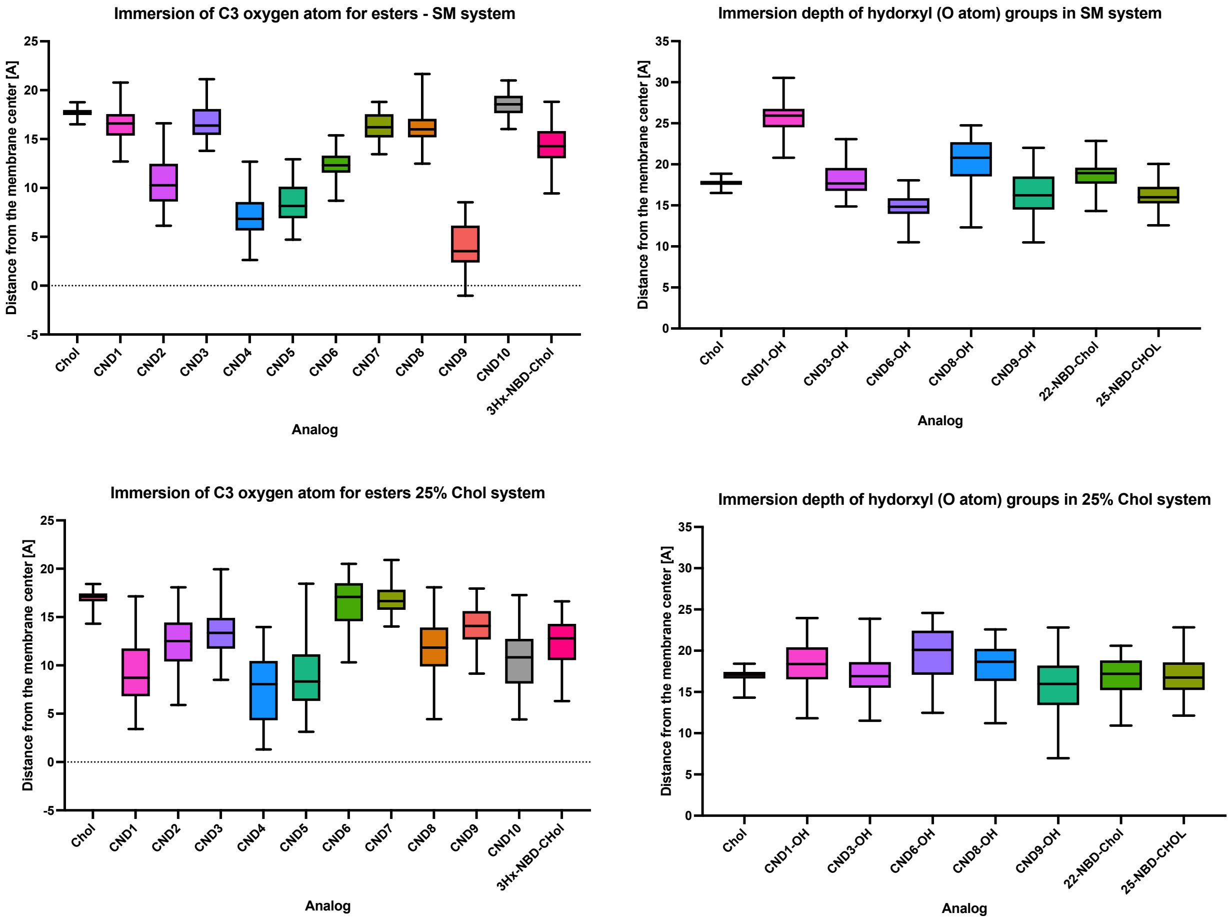

Fig. S4. Comparison of the immersion depth for all analogs in Chol:POPC, 1:3 (25% Chol) and the SM:Chol:POPC, 1:1:2 systems measured as position of C3-oxygen atom (left column). The position of available other hydroxyl groups present in some analogs with respect to cholesterol was also determined (right column). Different behaviors of probes, depending on lipid composition, are evident.

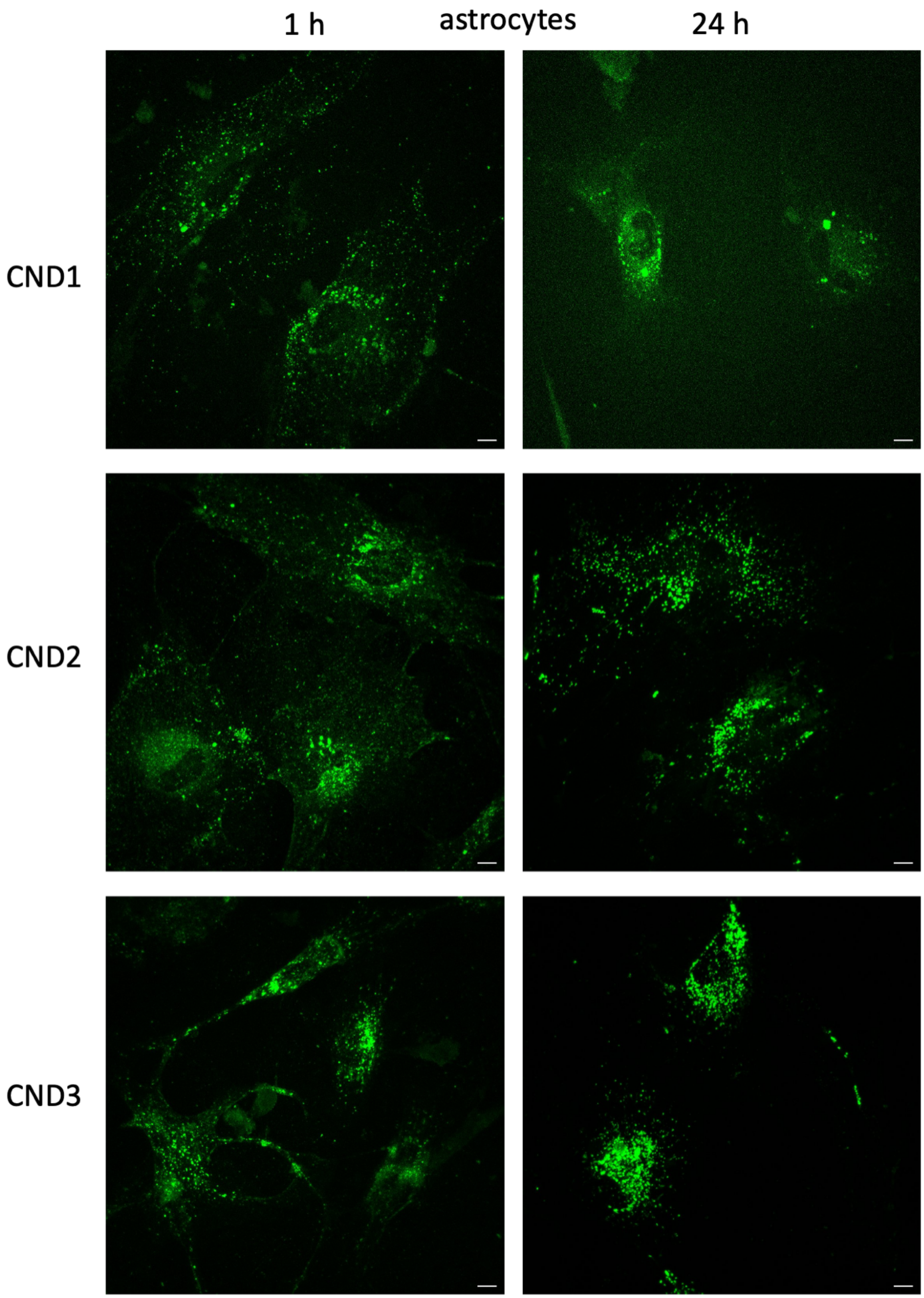
1 h
astrocytes
24 h
CND1
CND2
CND3

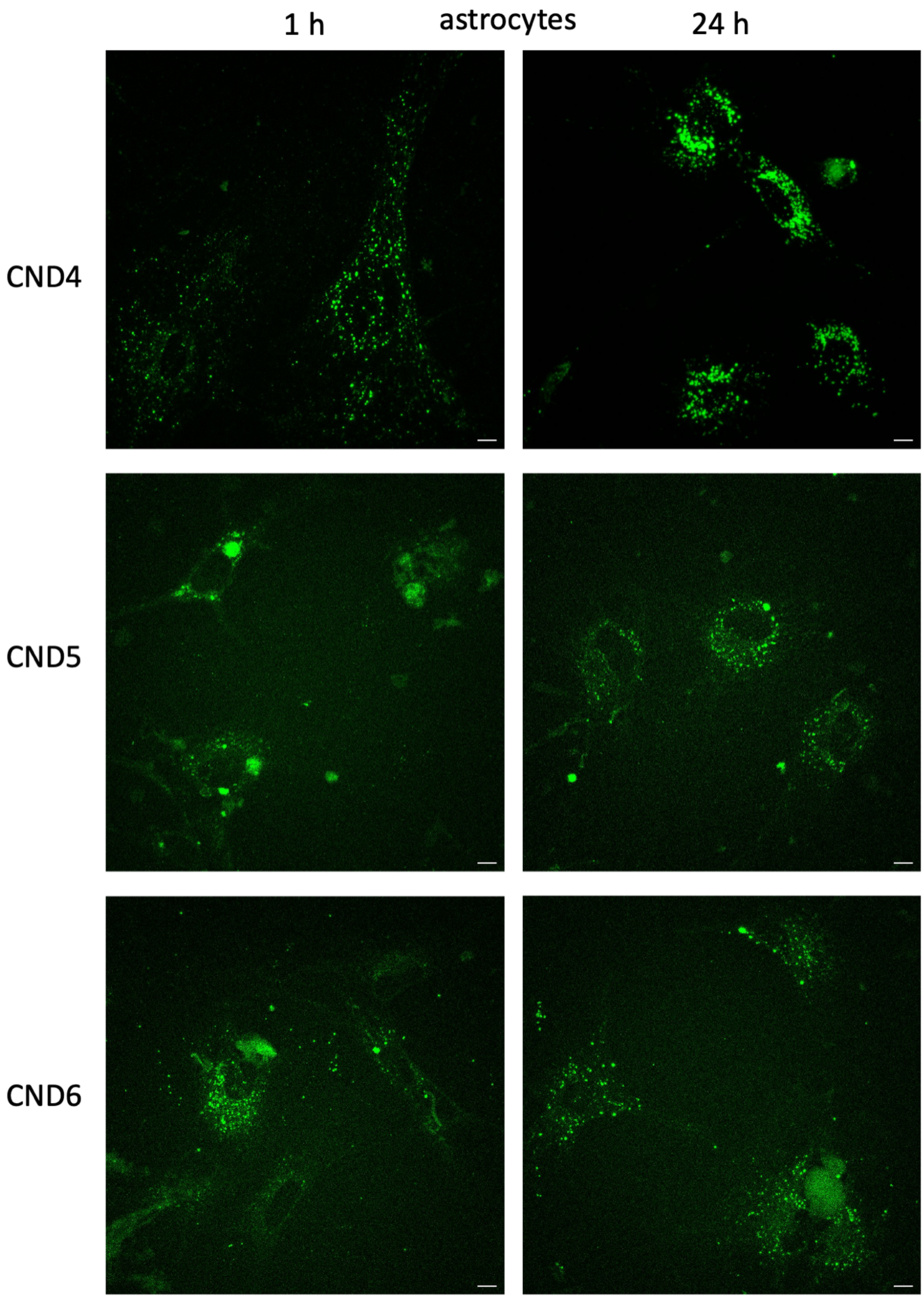
1 h
astrocytes
24 h
CND4
CND5
CND6

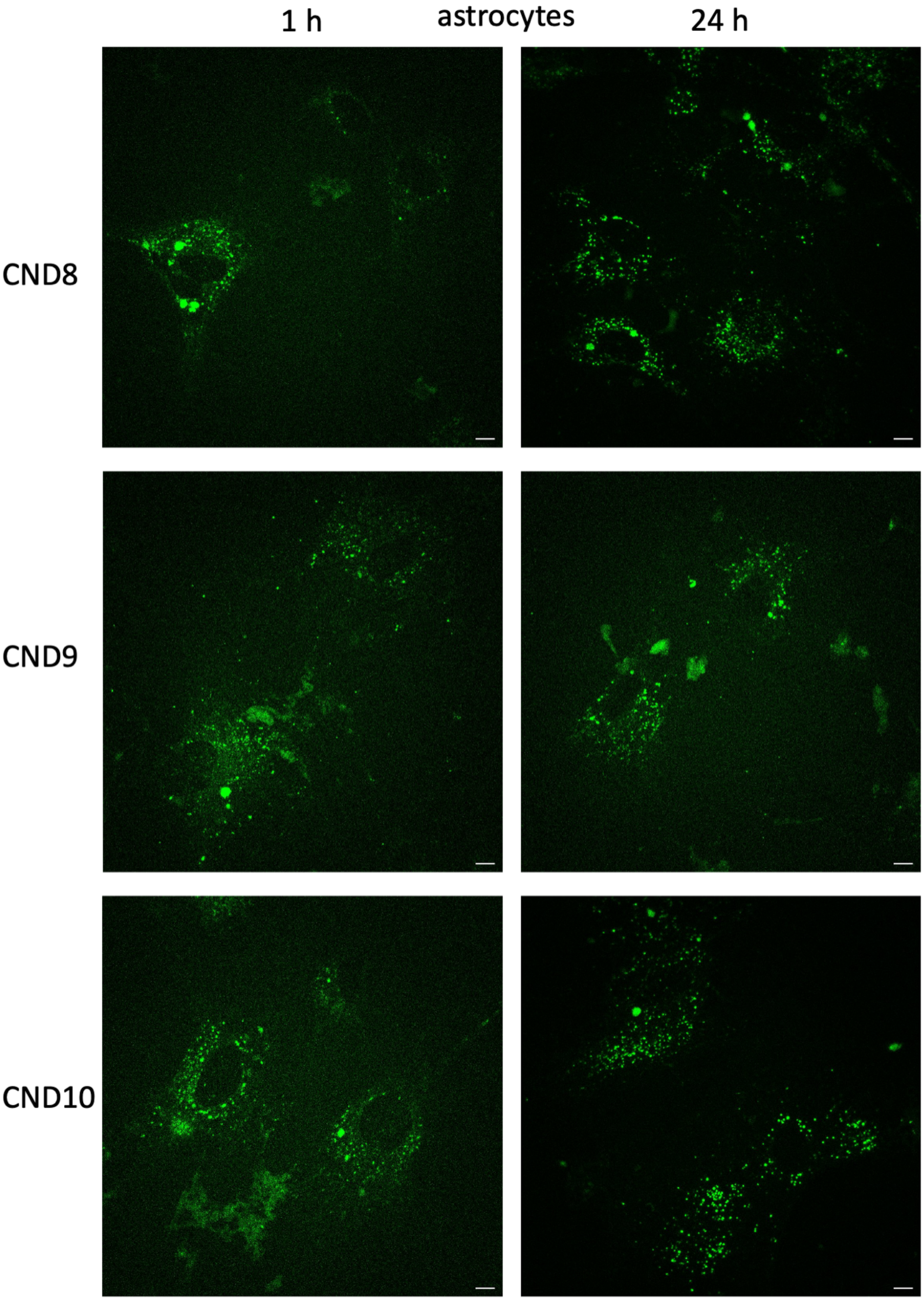
1 h
astrocytes
24 h
CND8
CND9
CND10

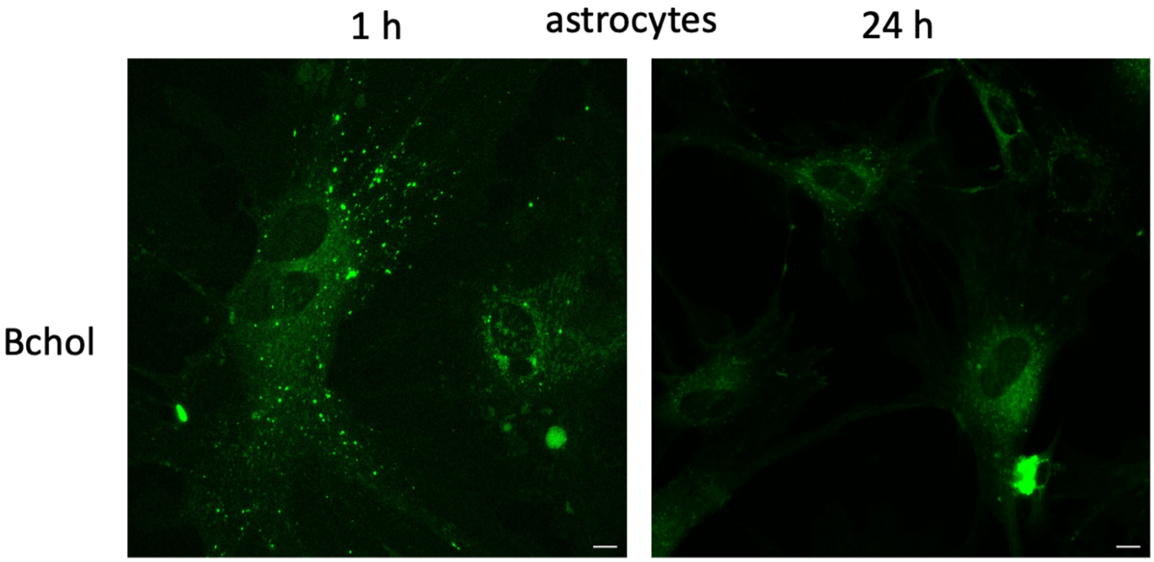
1 h
astrocytes
24 h
Bchol

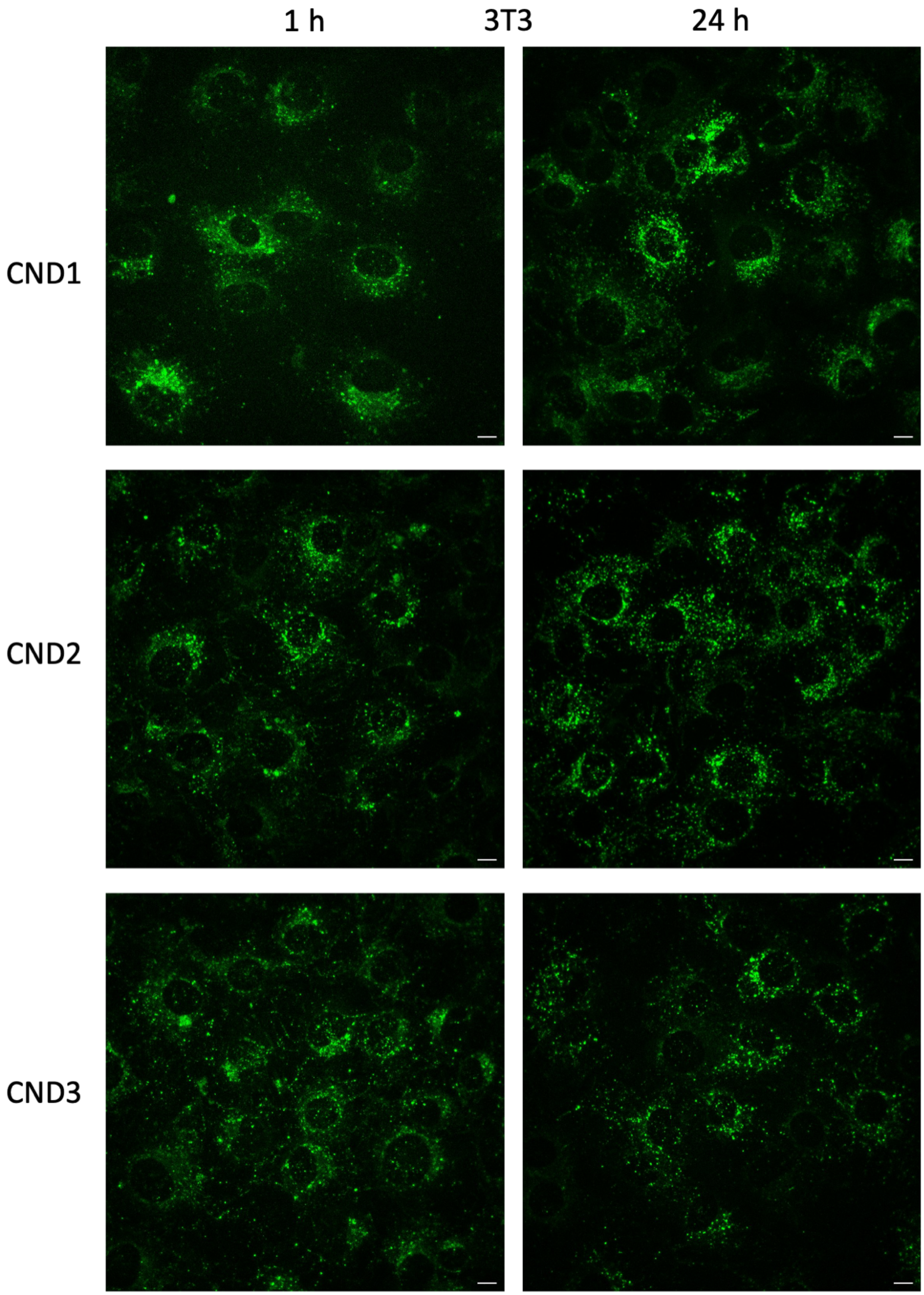
1 h
3T3
24 h
CND1
CND2
CND3

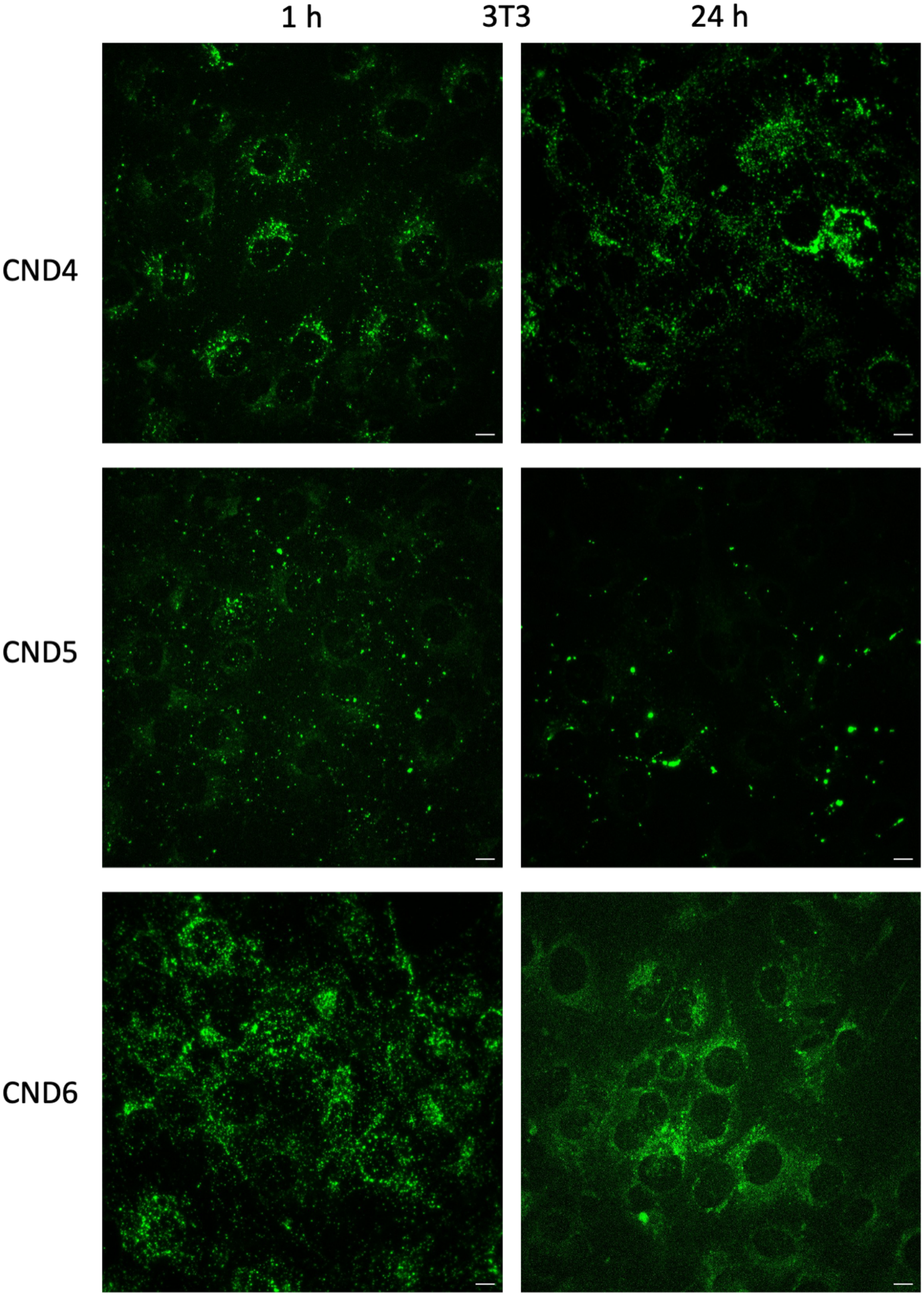
1 h
3T3
24 h
CND4
CND5
CND6

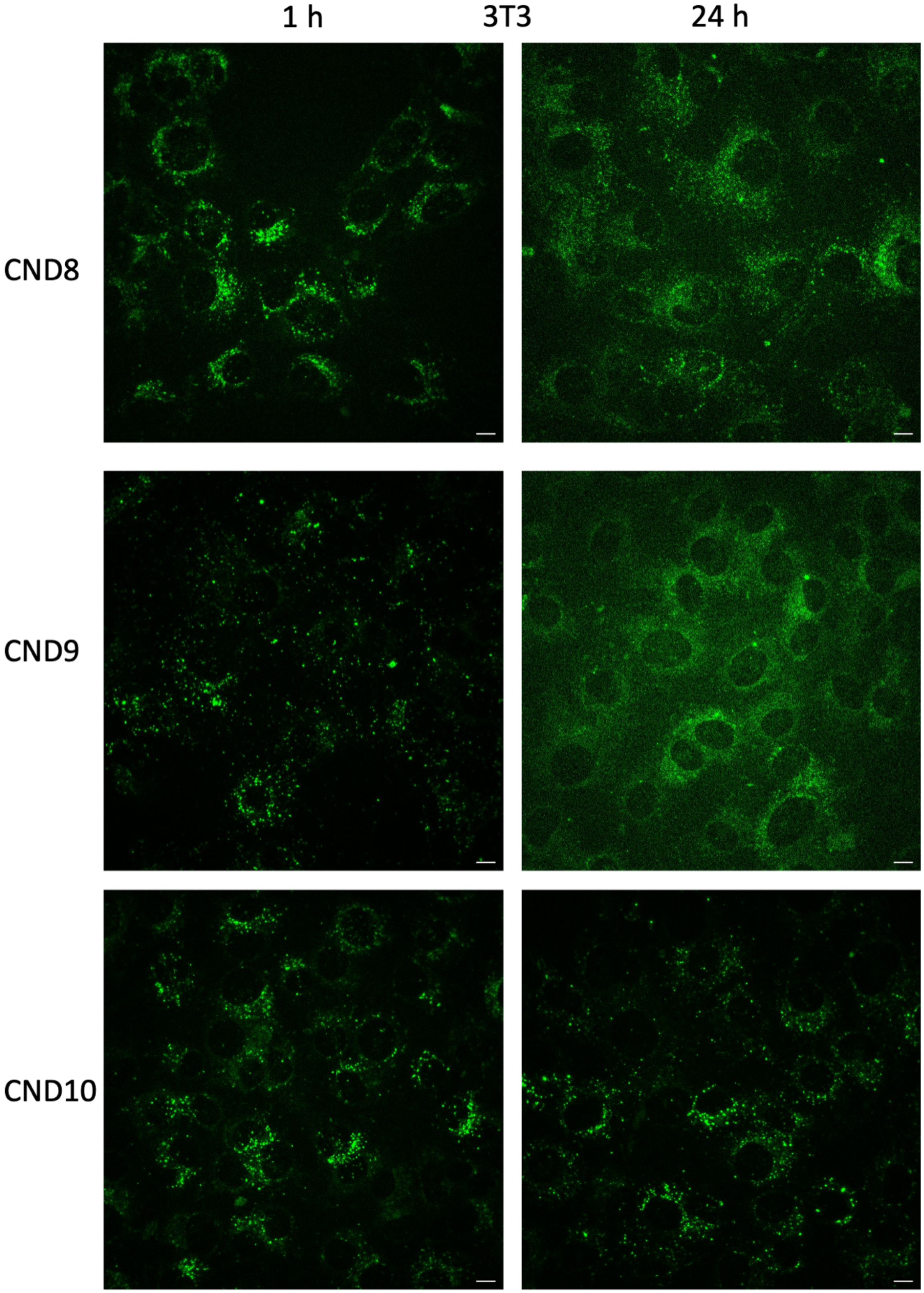
1 h
3T3
24 h
CND8
CND9
CND10

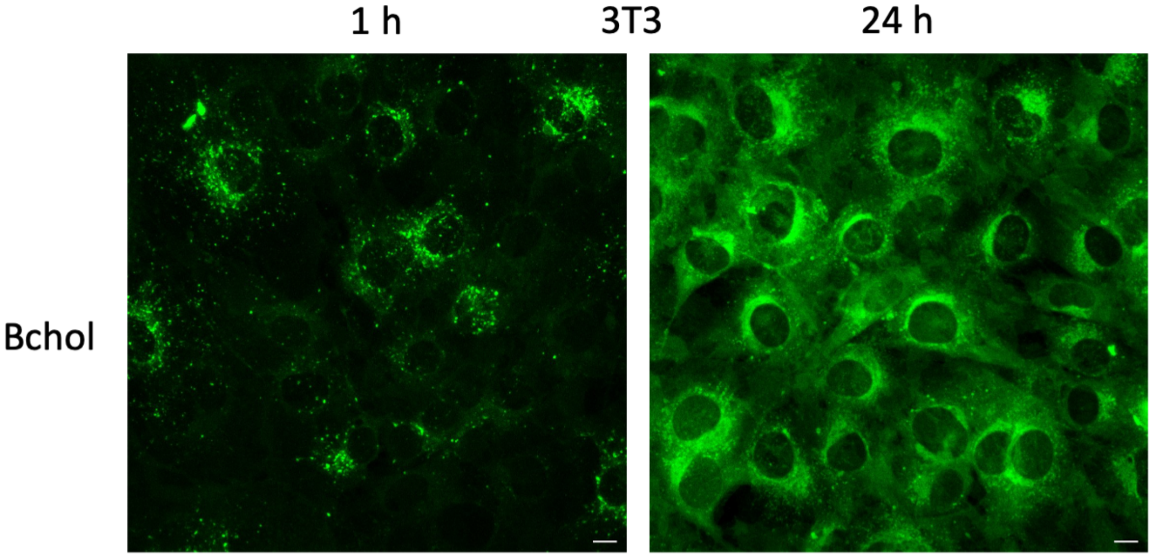

Fig. S5. Representative examples of fluorescent confocal images of green channels representing tested probes in astrocytes and 3T3 fibroblasts pulse chased after 1h and 24h. Images depict the average intensity of Z-stack. Bar size – 10 μm.

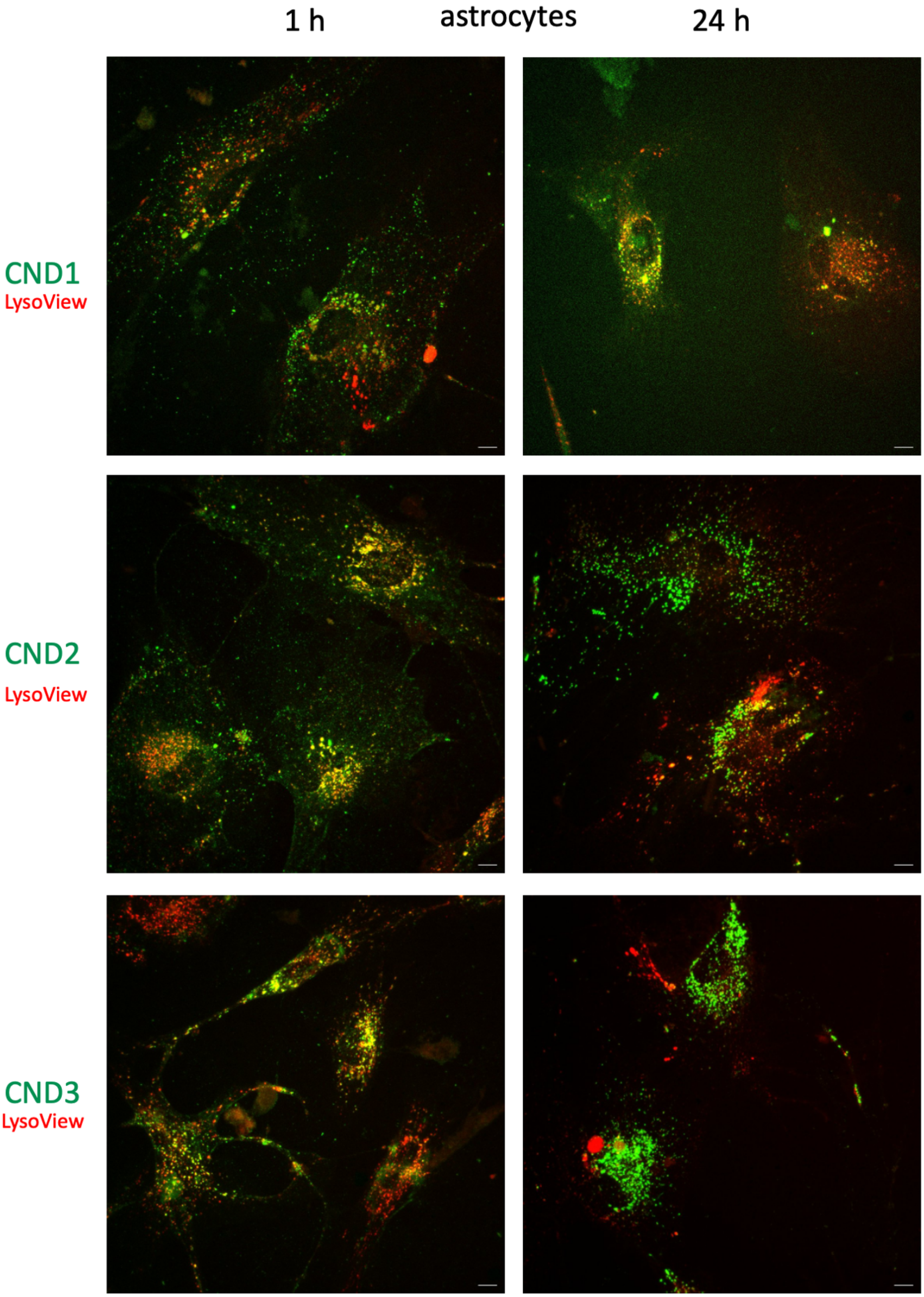
1 h
astrocytes
24 h
CND1
LysoView
CND2
LysoView
CND3
LysoView

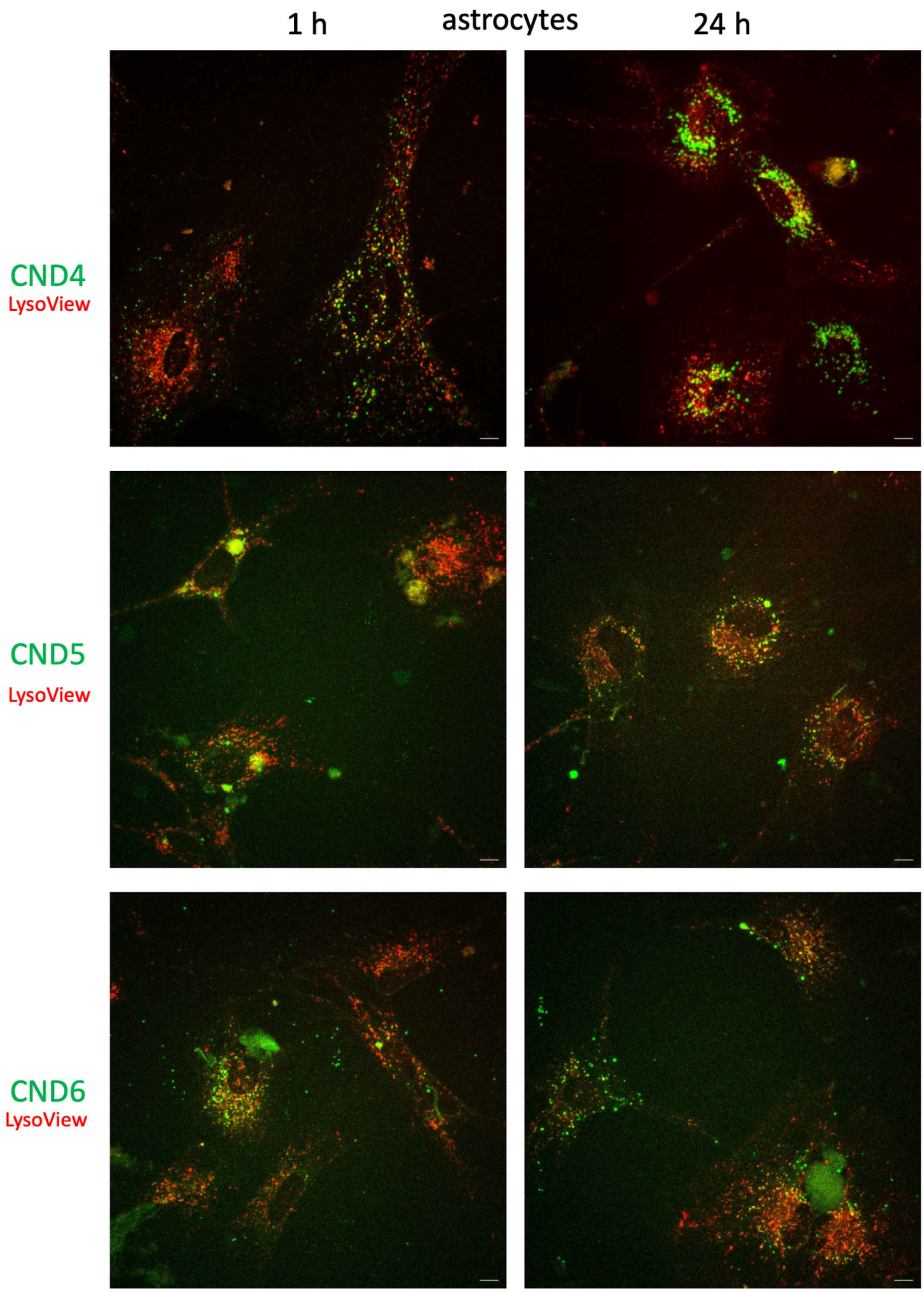
1 h
astrocytes
24 h
CND4
LysoView
CND5
LysoView
CND6
LysoView

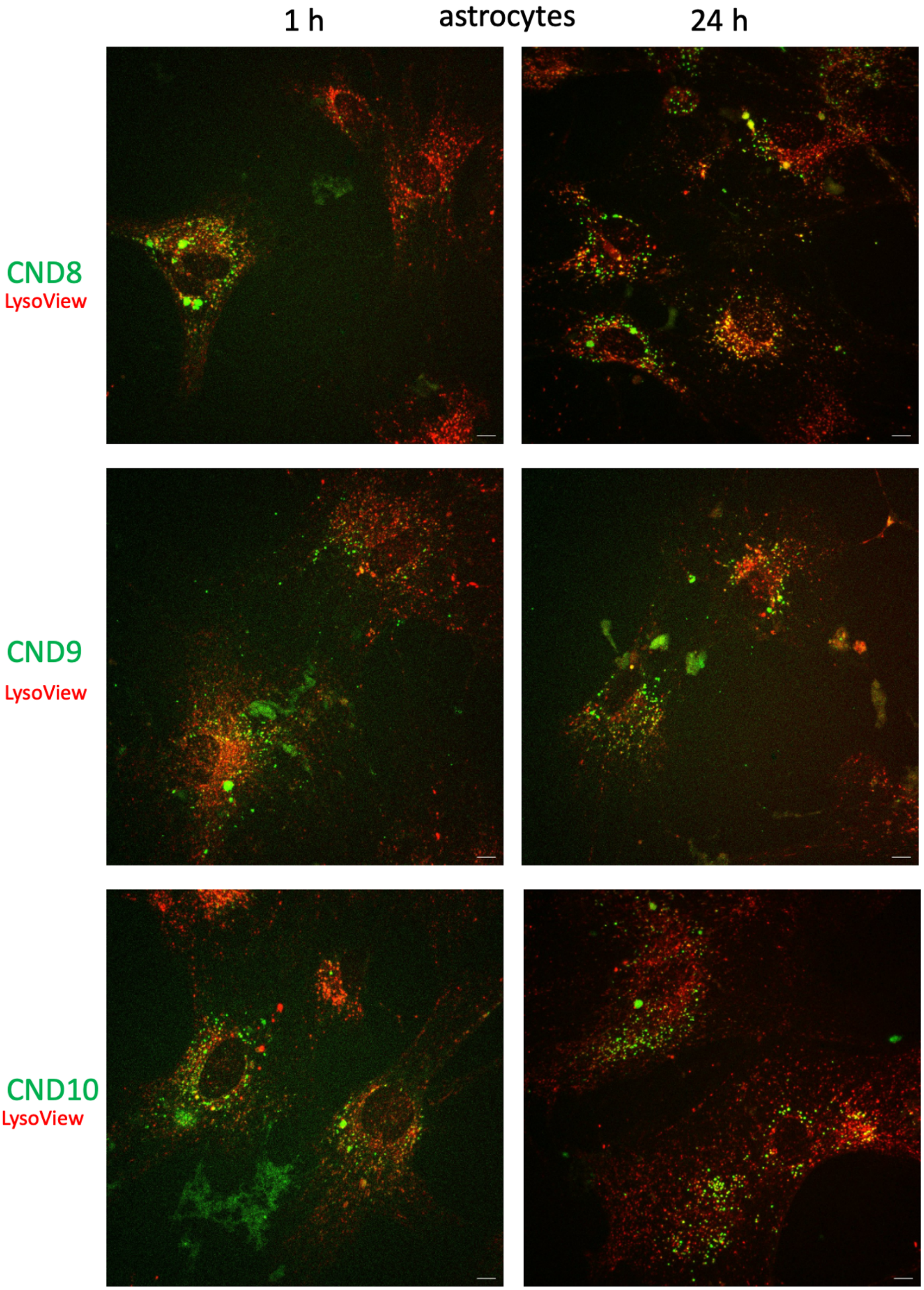
1 h
astrocytes
24 h
CND8
LysoView
CND9
LysoView
CND10
LysoView

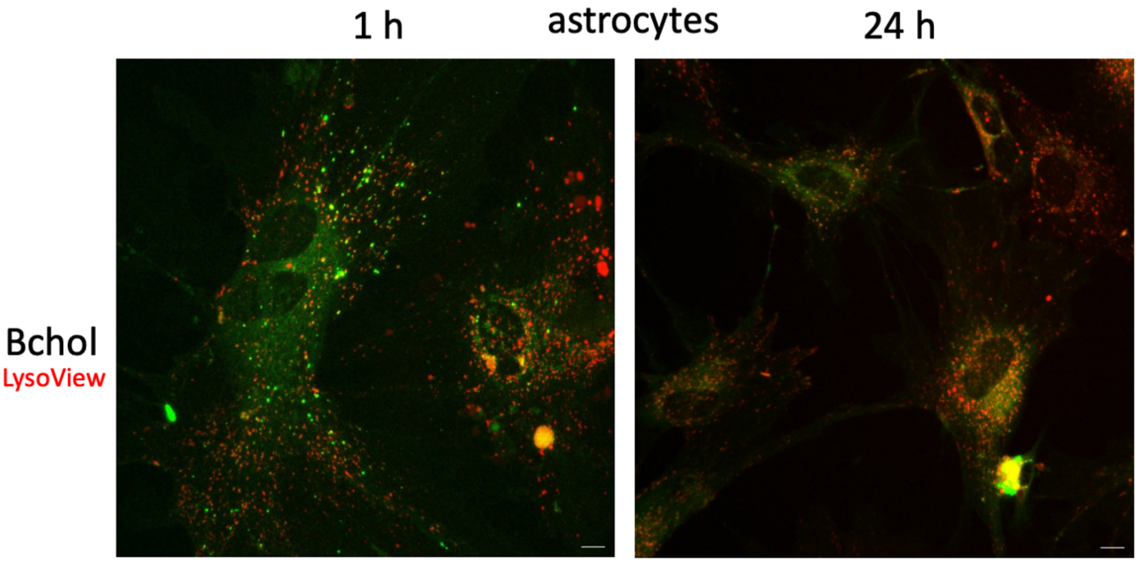
1 h
astrocytes
24 h
Bchol
LysoView

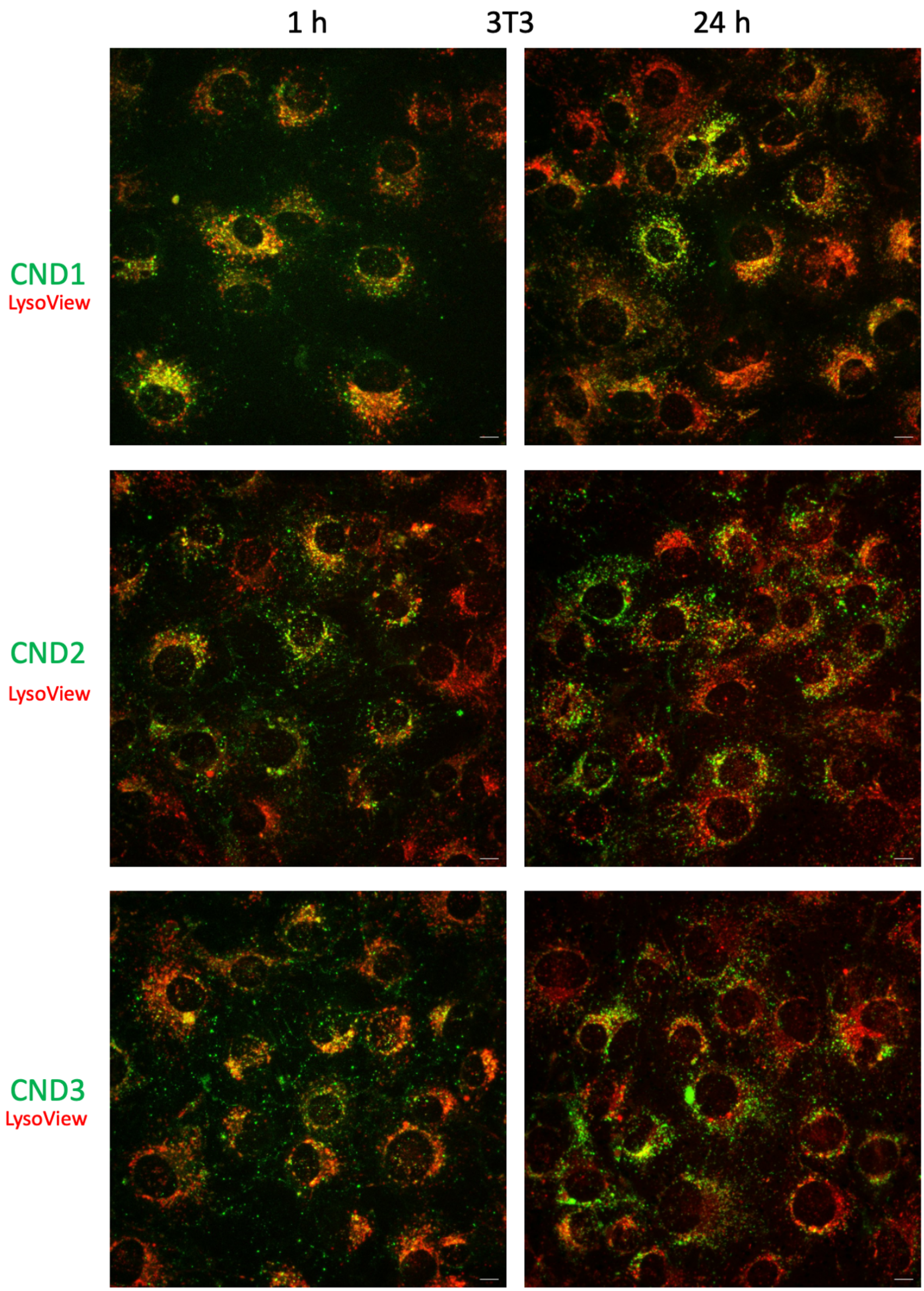

1 h
3T3
24 h
CND1
LysoView
CND2
LysoView
CND3
LysoView

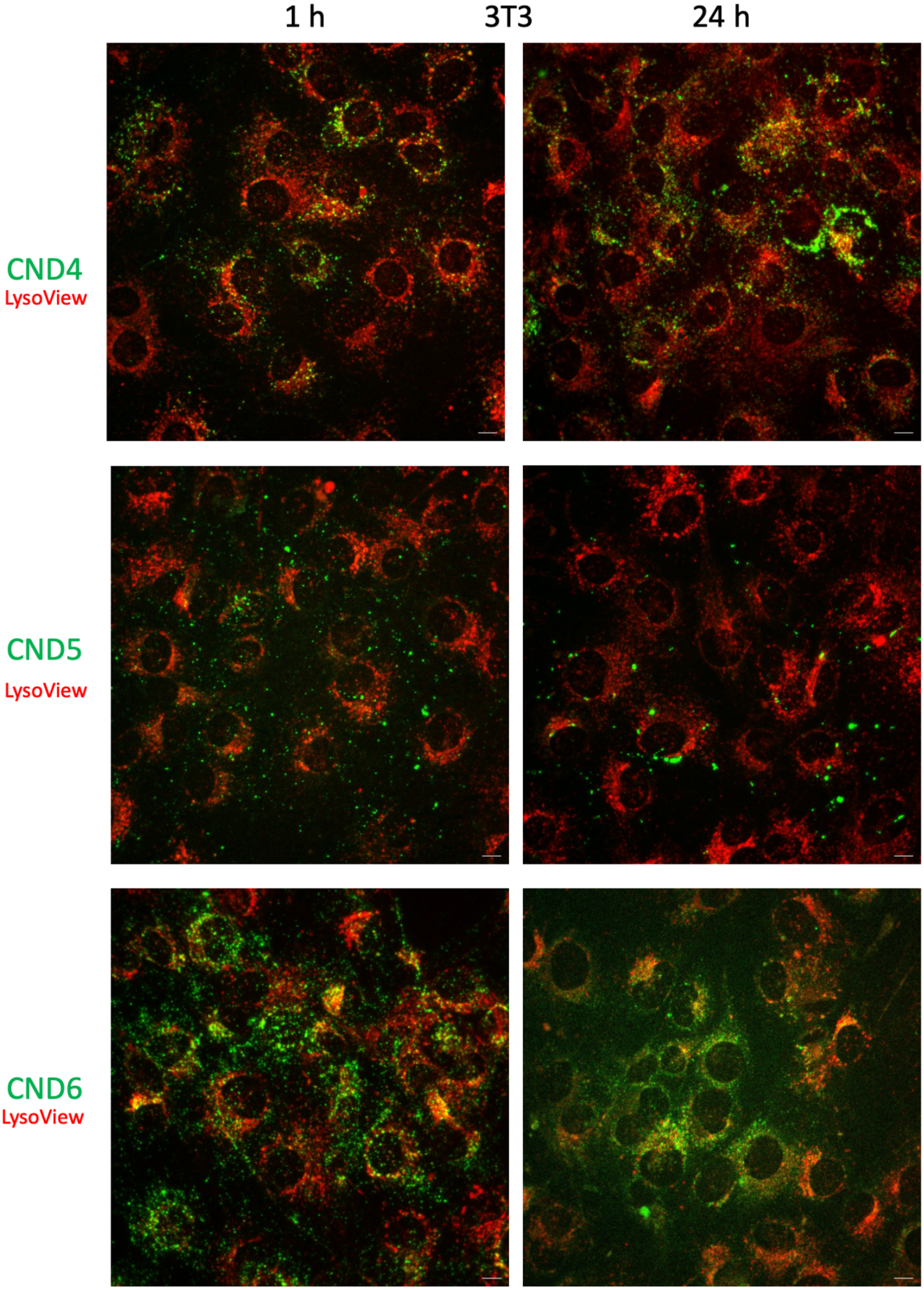
1 h
3T3
24 h
CND4
LysoView
CND5
LysoView
CND6
LysoView

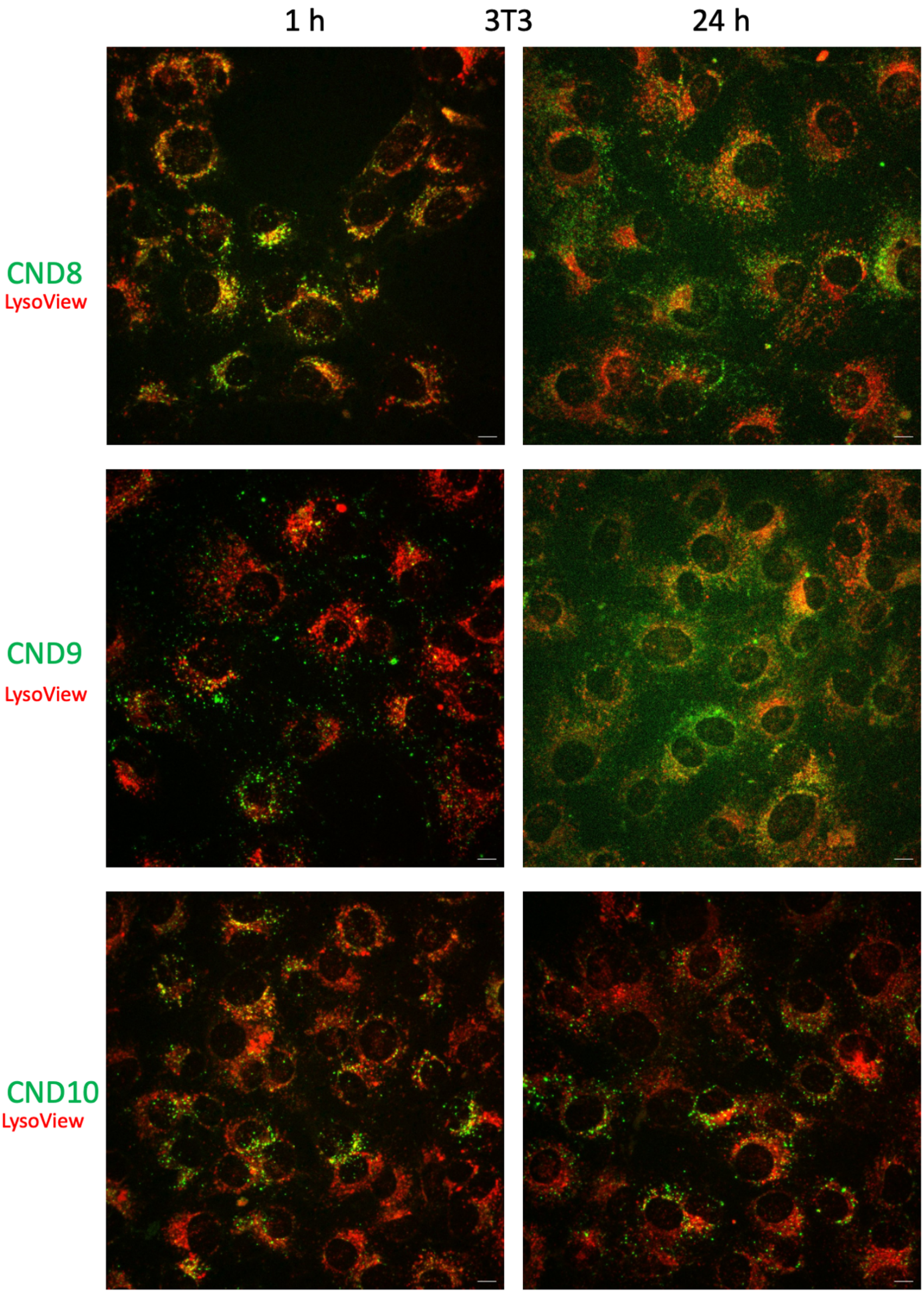

1 h
3T3
24 h
CND8
LysoView
CND9
LysoView
CND10
LysoView

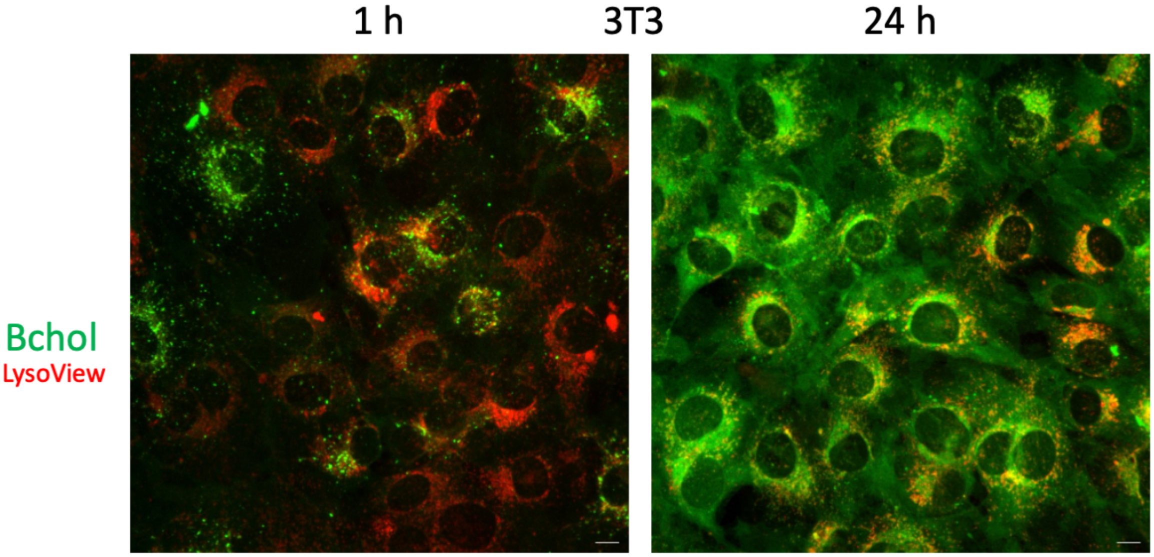

Fig. S6. Representative examples of fluorescent confocal composite images of analogs in astrocytes and 3T3 fibroblasts pulse chased for 1h and 24h. Tested probes are depicted in green, LysoView marker is in red. Images in each channel depict the average intensity of the Z-stack. Bar size – 10 μm.

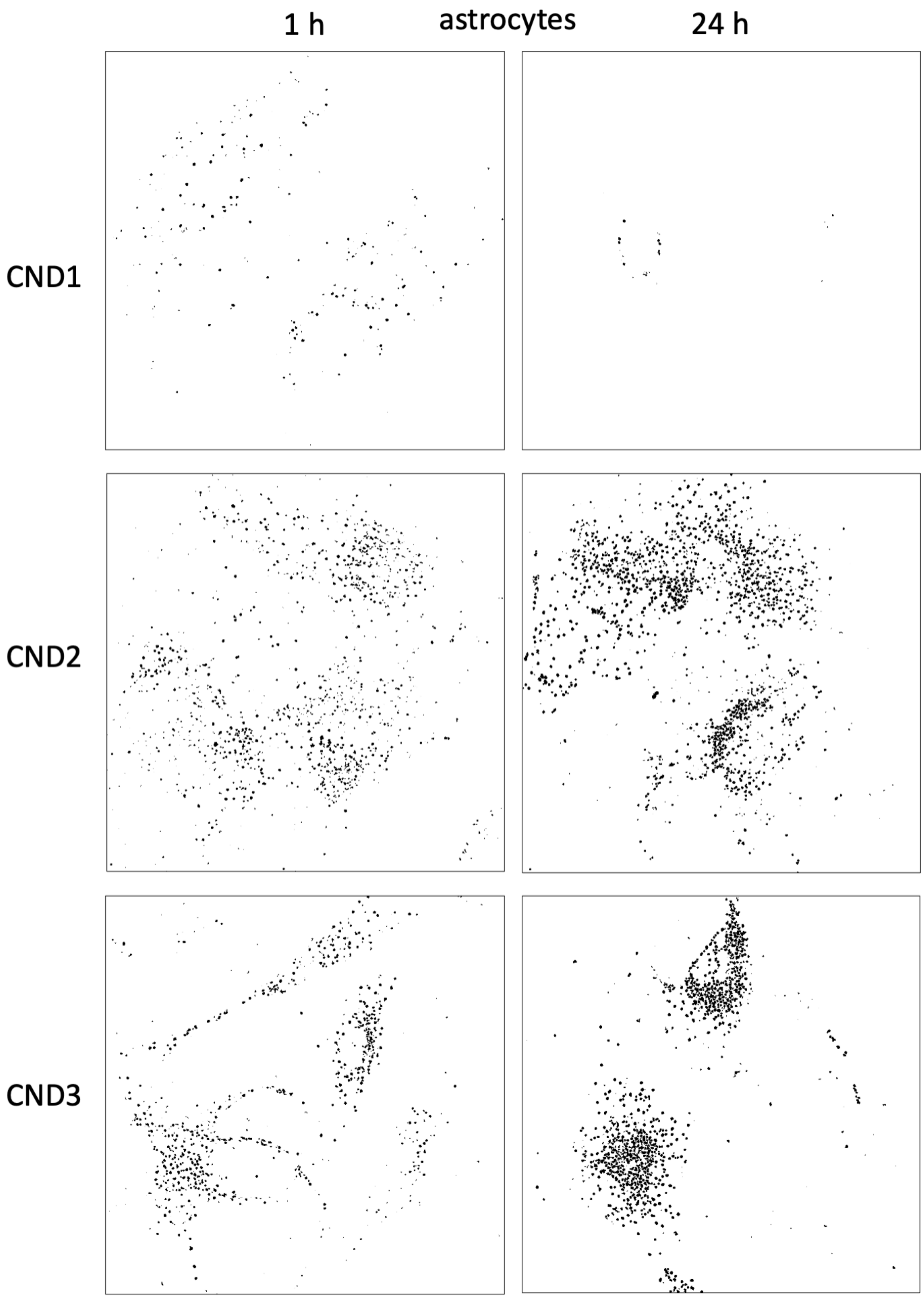
1 h
astrocytes
24 h
CND1
CND2
CND3

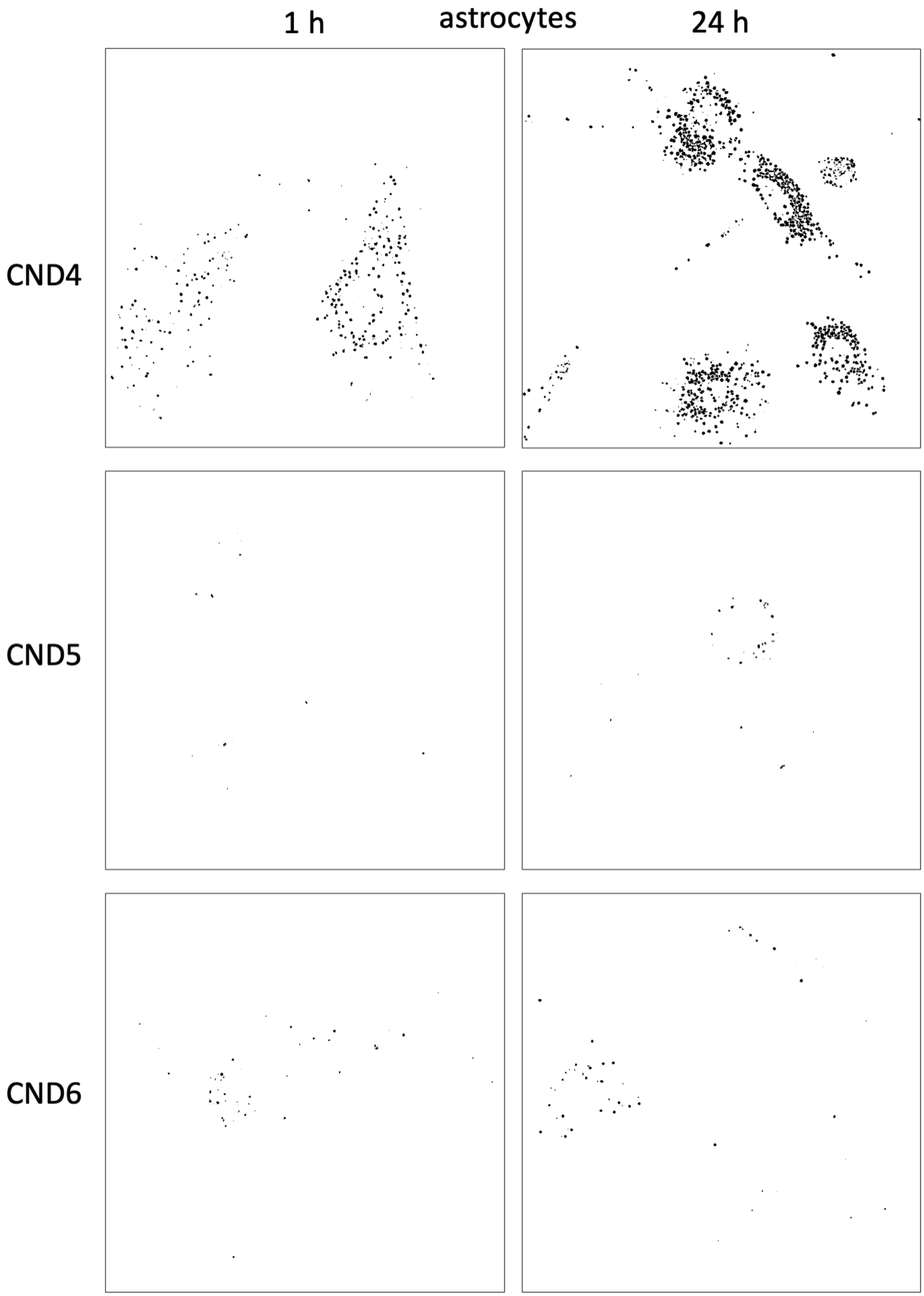
1 h
astrocytes
24 h
CND4
CND5
CND6

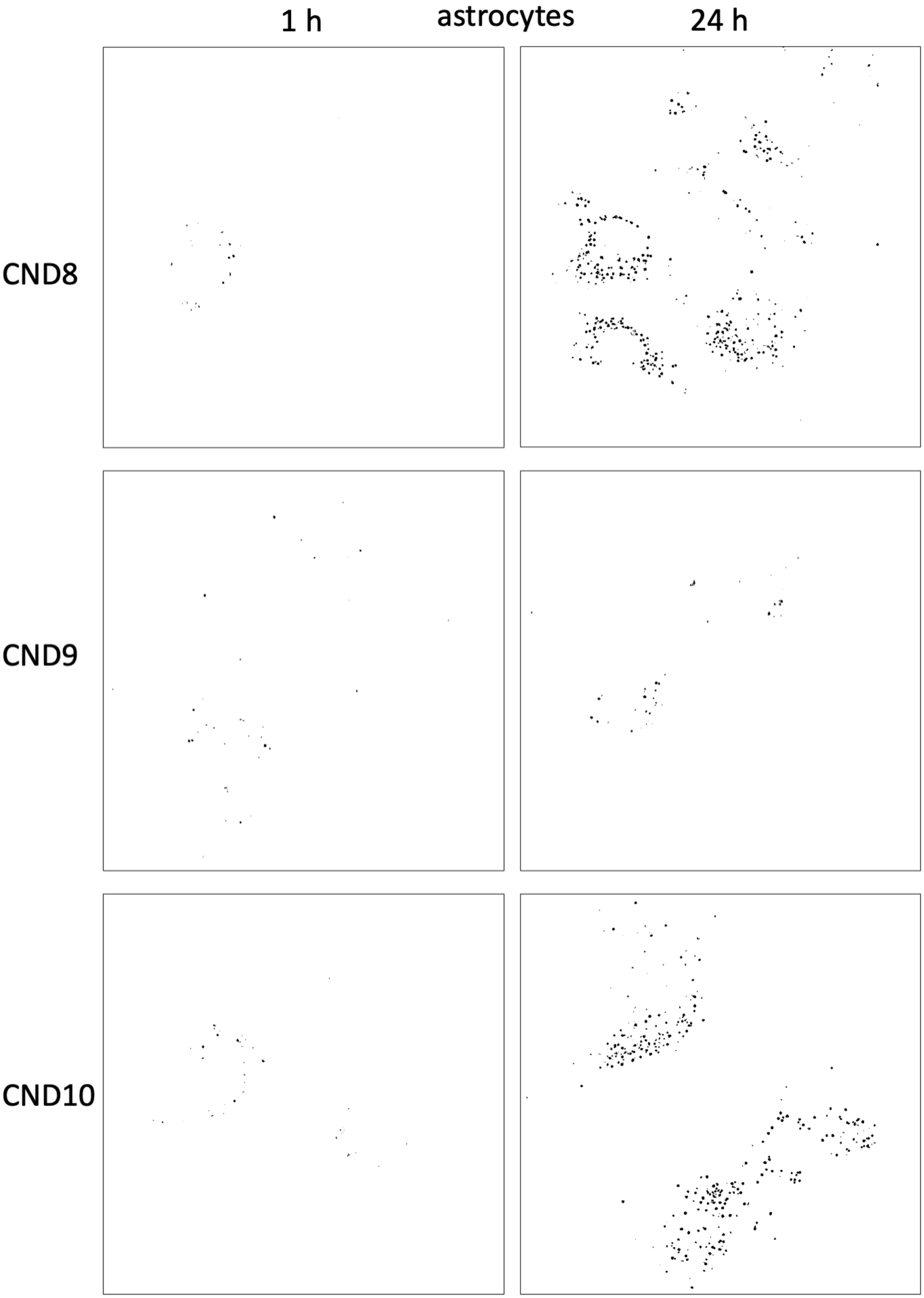

1 h
astrocytes
24 h
CND8
CND9
CND10

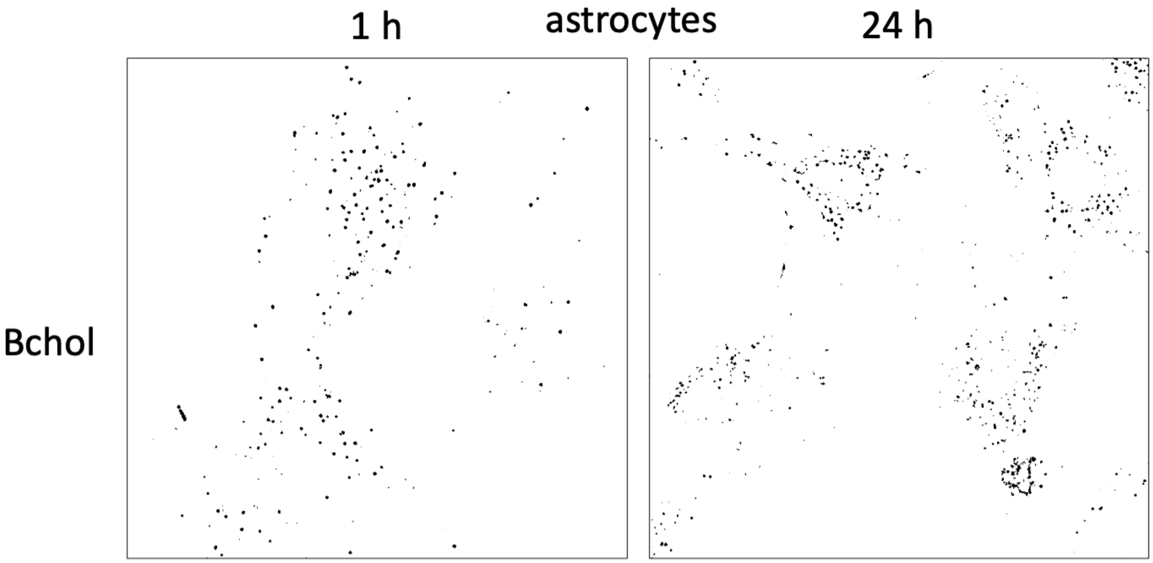
1 h
astrocytes
24 h
Bchol

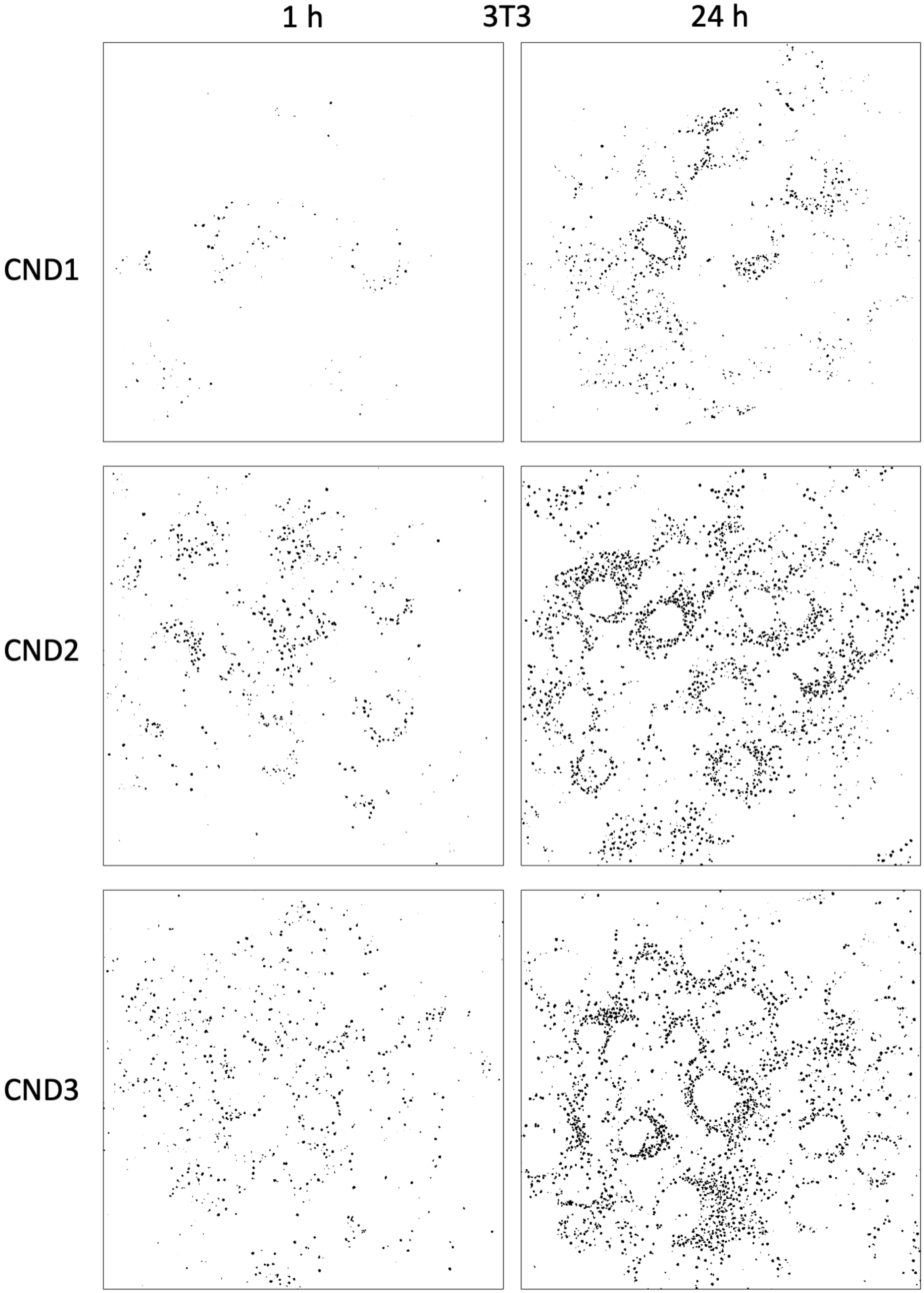

1 h
3T3
24 h
CND1
CND2
CND3

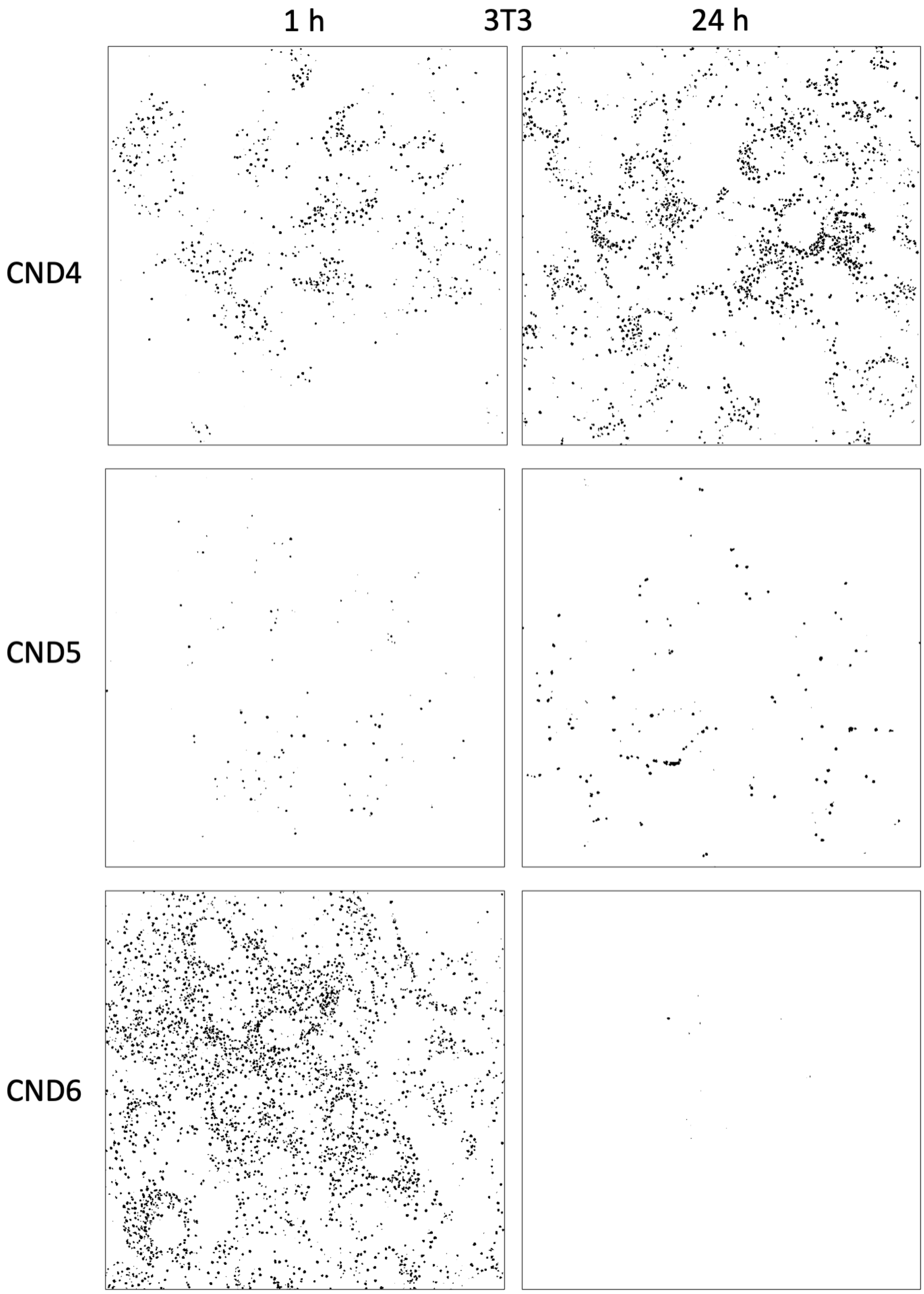
1 h
3T3
24 h
CND4
CND5
CND6

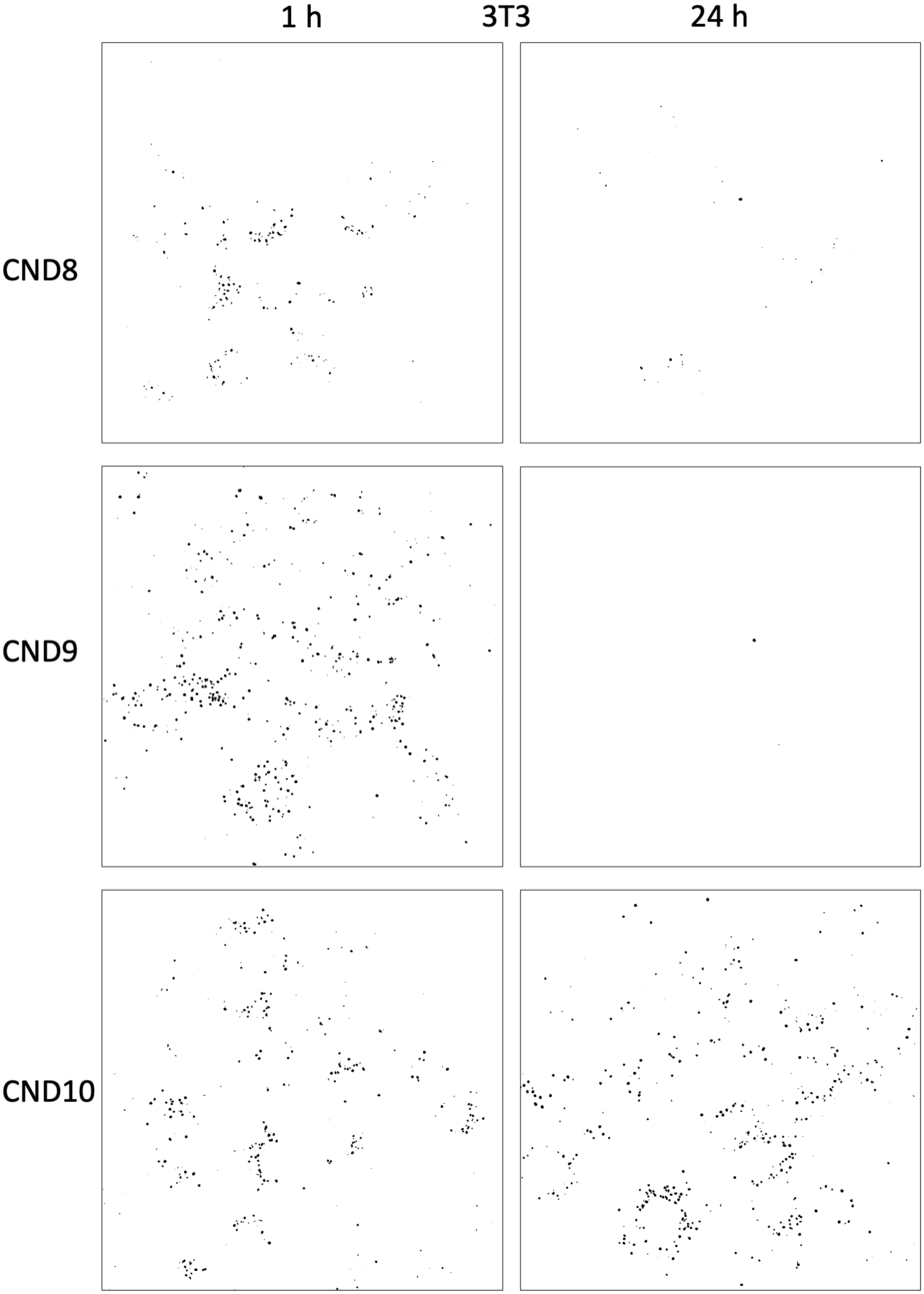
1 h
3T3
24 h
CND8
CND9
CND10

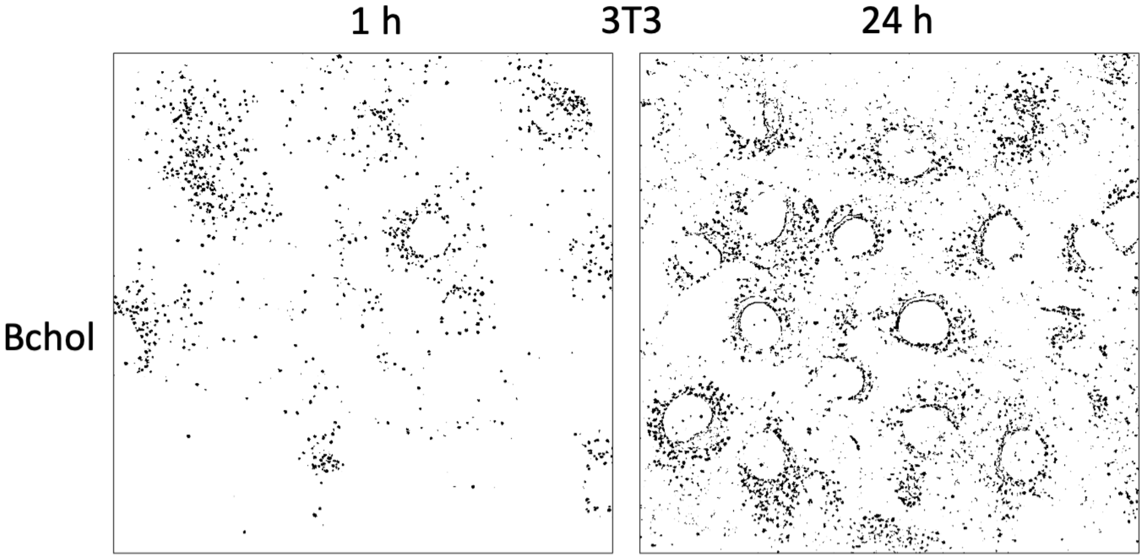

Fig. S7. Representative examples of fluorescent puncta masks obtained through Weka trainable segmentation used to determine particle masks/ROIs. These masks were generated from a single-frame image of the highest intensity profile frame in the green channel in the Z-stack image.

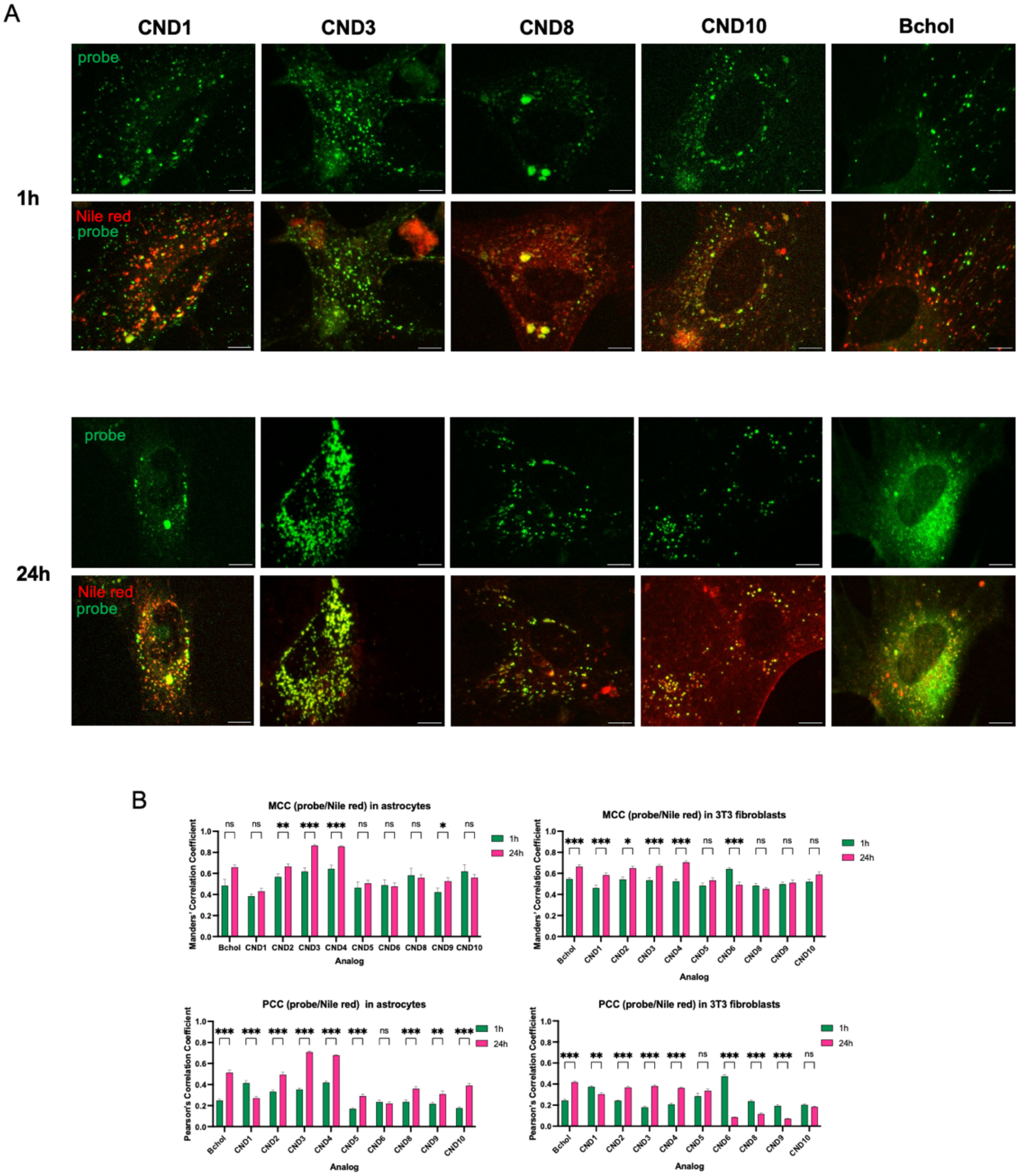

Fig. S8. Live cell imaging of CND probes in cultured astrocytes. **A**, sample images for analogs that are neutral (CND1, 8), positively charged (CND3), and negatively charged (CND10) compared with Bchol. Overlay images show co-labeled Nile red (red) overlapping with selected CNDs better than Bchol. Scale bar, 10 μm. **B**, Manders' and Pearson's correlation coefficients between probe/LysoView at different time points and cell types.

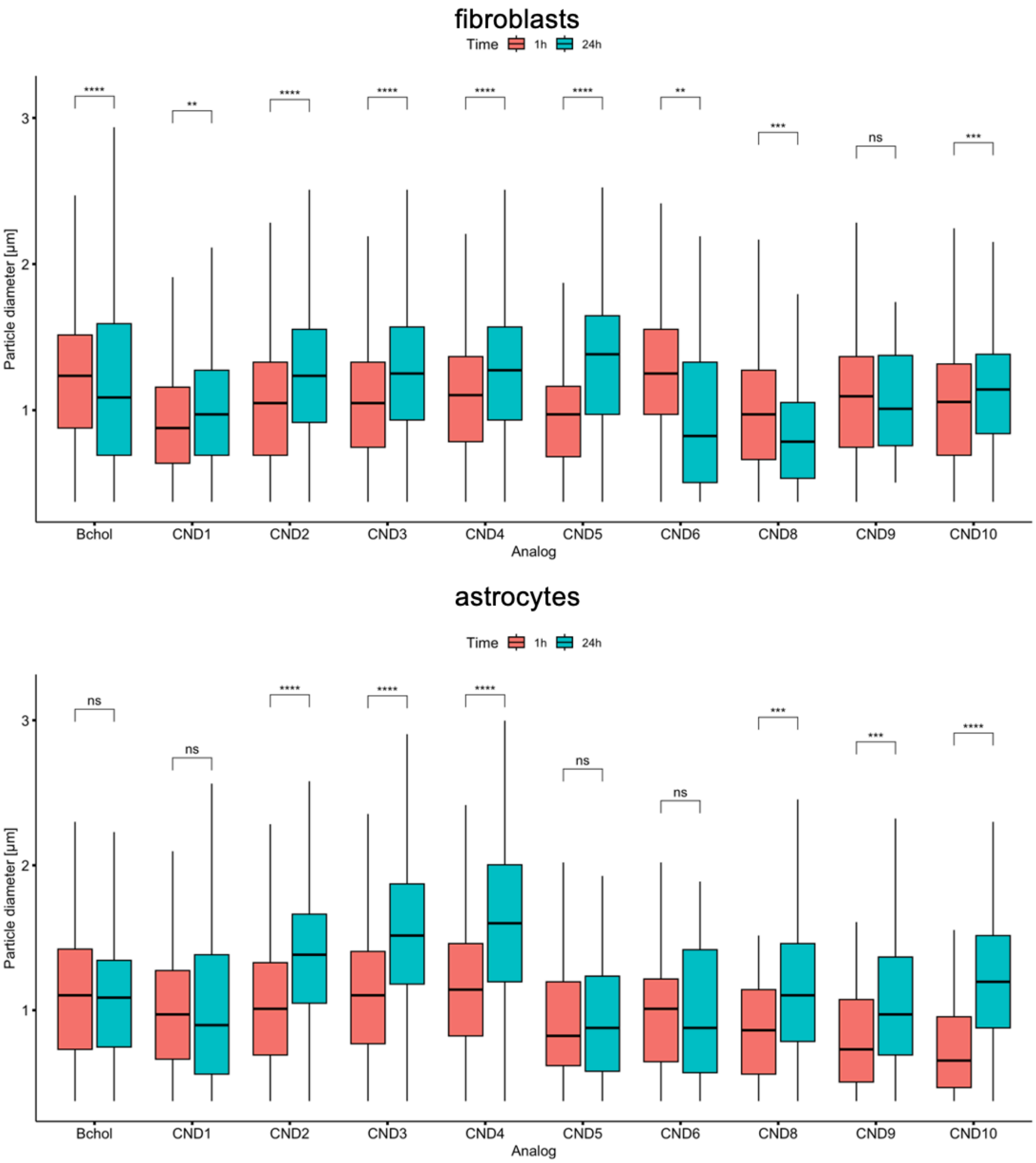

Fig. S9. Change in the particle size (diameter) for all tested CND analogs and compared to Bchol at 1h and 24h for both cell lines. Particles were identified in a range between 0-3μm. Statistical analysis was performed using a t-test (rstatix package in R). p-values: **** ($p<0.0001$), *** ($p<0.001$), ** ($p<0.01$), *($p<0.05$), ns – not significant.

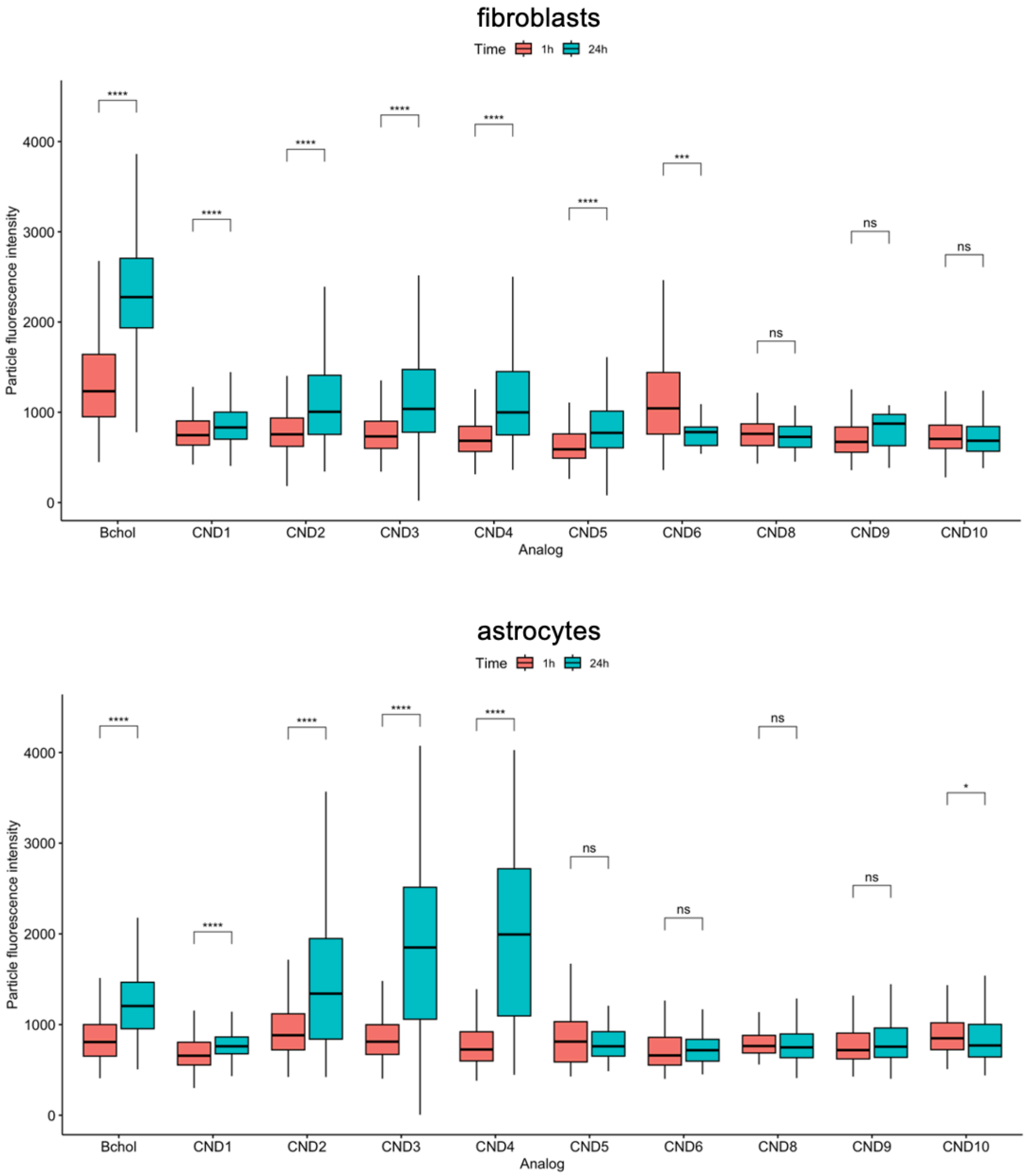

Fig. S10. Change in the particle intensity for all tested CND analogs and compared to Bchol at 1h and 24h for both cell lines. Particles were identified in a range between 0-3μm. Statistical analysis was performed using a t-test (rstatix package in R). p-values: **** (p<0.0001), *** (p<0.001), ** (p<0.01), *(p<0.05), ns – not significant.

Chemical synthesis of CND probes

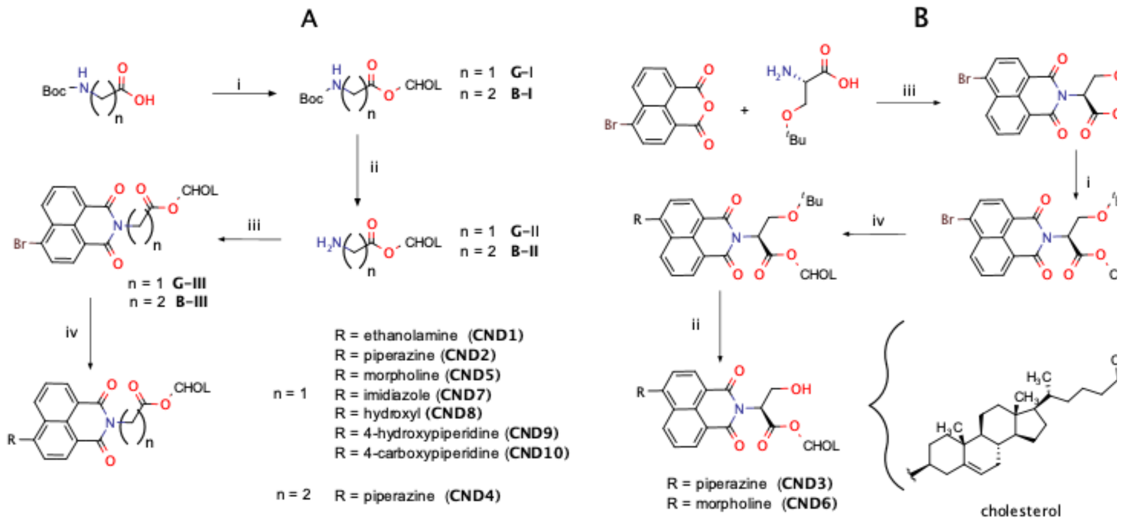

Scheme S1. Synthetic scheme for glycine and beta alanine linkers (**A**) and serine linker (**B**). Reagents and conditions: (i) DCC, DMAP, DCM, N_2, 0°C, (ii) TFA, TIS, DCM, rt, (iii) 1,8-naphthalic anhydride, EtOH, reflux, (iv) amine (headgroup) DMSO, 100 °C, catalyst (CND7: metformin, CND8:N-hydroxyphthalimide)

Synthesis of Glycine linker Analogs: CND1, CND2, CND5, CND7 - CND10

Cholesteryl Boc-glycinate and cholesteryl glycinate were synthesized as previously described, and this method was adapted for the esterification of all carboxylic acid-containing intermediates.[1]

(**G-I**) *Cholesteryl Boc-glycinate:* In a round bottom flask, cholesterol (500 mg, 1.30 mmol), boc-glycine (343 mg 1.95 mmol), and DMAP (16 mg 0.130 mmol) were dissolved in anhydrous DCM (10 mL). The solution was stirred purged with N_2 and maintained at 0°C in an ice bath. DCC (430 mg, 2.08 mmol) was dissolved in anhydrous DCM (5 mL) and added to the reaction mixture slowly. The reaction stirred for 30 minutes on the ice bath before being removed and stirring at room temperature overnight. Dicyclohexylurea was filtered off and the filtrate was collected. The filtrate was washed three times with 10% HCl (20 mL) and the organic layer was dried over Na_2SO_4 and then was removed by vacuum to give white crystals. Boc-Gly-Chol was purified on silica column in 10% EtOAc in hexanes (1:9 v:v). Solvent was dried over Na_2SO_4 and removed by vacuum to obtain 536 mg of a white solid (yield = 76%). ^{1}H-NMR (400 MHz $CDCl_3$): δ 5.39 (s, 1H), 5.04 (s, 1H), 4.70 (m, 1H), 3.88 (d, 2H), 2.35 (d, 2H) 0.64 – 2.1 (m, 50H) ^{13}C-NMR (400 MHz $CDCl_3$): δ 169.7, 155.6, 139.3, 122.9, 79.8, 75.1, 56.6, 56.1, 50.0, 42.6, 42.3, 39.7, 39.5, 38.0, 36.5, 36.1, 35.7, 31.89, 31.85, 28.3, 28.2, 28.0, 27.7, 24.2, 23.8, 22.8, 22.5, 21.0, 19.2, 18.7, 11.8. $C_{34}H_{57}NO_4$ HRMS-ESI: $[M+Na]^+$ $C_{34}H_{57}NO_4$ calcd. m/z = 566.4180 Da, obs. m/z = 566.4188 Da

(**G-II**) *Cholesteryl glycinate:* In a round bottom flask, 536 mg (0.99 mmol) **G-I** was dissolved in 20 mL DCM, 270 µL (1.30 mmol) triisopropylsilane and 3 mL TFA at room

temperature. Reaction was stirred for 2 hours before being quenched with 50 mL H_2O and saturated sodium bicarbonate to remove TFA. The organic layer is then evaporated and then purified on silica (MeOH:EtOAC 1:1 v:v) to yield 420 mg of a white solid (yield = 96%). ^{1}H-NMR (400 MHz $CDCl_3$): δ 5.40 (s, 1H) 4.69 (m, 1H), 3.41 (s, 2H), 2.35 (d, 2H), 0.64 – 2.1 (m, 41H) ^{13}C-NMR (400 MHz $CDCl_3$): δ 173.7, 139.4, 122.8, 74.5, 56.6, 56.1, 50.0, 44.2, 42.3, 39.7, 39.5, 38.1, 36.9, 36.5, 36.1, 35.7, 31.9, 31.8, 28.2, 28.0, 27.7, 24.2, 23.8, 22.8, 22.5, 21.0, 19.2, 18.7, 11.8. $C_{29}H_{49}NO_2$ HRMS-ESI: calcd. m/z = 466.3662 Da, obs. m/z = 466.3656

(**G-III**) *4-bromo-1,8-naphthalimide cholesteryl glycinate:* In a round bottom flask, 34mg (0.077 mmol) of **G-II** and 21mg (1 equivalent) of 4-bromo-1,8-naphthalic anhydride was dissolved in ethanol and refluxed overnight. After reaction is complete, ethanol is removed by vacuum. The crude product is purified on silica in (10:10:1 $CHCl_3$:Hexanes:EtOAc) and dried on Na_2SO_4. Solvent was removed by vacuum to yield 24 mg of a white solid (yield = 44%). ^{1}H-NMR (400 MHz $CDCl_3$): δ 8.66 (d, 1H), 8.58 (d, 1H), 8.42 (d, 1H), 8.06 (d, 1H), 7.86 (t, 1H), 5.39 (s, 1H), 4.92 (s, 2H), 4.73 (m, 1H), 2.4 (d, 2H) 0.64 – 2.1 (m, 41H) ^{13}C-NMR (400 MHz $CDCl_3$): δ 167.2, 163.2, 139.4, 133.6, 132.3, 131.5, 131.1, 130.7, 129.1, 128.1, 122.8, 122.8, 122.6, 121.83, 75.5, 56.7, 56.1, 50.0, 42.3, 41.6, 39.7, 39.5, 37.9, 36.9, 36.5, 36.1, 35.7, 31.9, 31.8, 28.2. 28.0, 27.7, 24.2, 23.8, 22.8, 22.5, 21.0, 19.3, 18.7, 11.8, $C_{41}H_{52}BrNO_4$, HRMS-ESI: calcd. m/z = 726.2929 Da, obs. m/z = 726.2929 Da

(**CND1**) *4-ethanolamine-1,8-naphthalimide cholesteryl glycinate:* In a round bottom flask, 58 mg (0.0827 mmol) of **G-III** was dissolved in 2 mL of DMSO. 3 equivalents of neat ethanolamine (15 µL) was added to reaction and was stirred and heated to 100°C. The reaction ran for two hours and was then diluted with 250 mL of water to give < 1% DMSO solution. The aqueous layer was extracted three times with EtOAc (75mL). 35 mg of crude was used for purification on a CombiFlash®Rf+ system in a DCM:MeOH gradient. 100% DCM was allowed to run for three minutes and then the gradient was increased to 5% MeOH over a period of 20 minutes. The gradient ran isocratically at 5% MeOH for an additional 5 minutes before increasing to 100% MeOH over the next 25 minutes. The solvent was evaporated to give an orange solid in a yield of 34 mg (yield = 58%): ^{1}H-NMR (400 MHz $CDCl_3$): δ 8.08 (dd, 2H), 7.92 (d, 1H), 7.08 (t, 1H), 6.43 (d, 1H), 6.35 (t, 1H) 5.46 (s, 1H), 4.89 (m, 3H), 3.96 (s, 2H), 3.74 (s, 1H), 3.47 (q, 2H), 2.53 (d, 2H), 0.64 – 2.1 (m, 41H) ^{13}C-NMR (400 MHz $CDCl_3$): δ 169.4, 164.3, 150.7, 139.5, 134.5, 130.8, 128.9, 127.1, 124.3, 122.9, 121.0, 119.8, 108.3, 104.0, 75.9, 60.6, 56.7, 56.2, 50.1, 46.6, 42.3, 41.4, 39.7, 39.5, 38.1, 37.0, 36.6, 36.2, 35.7, 31.9, 31.8, 28.2, 28.0, 27.7, 24.2, 23.8, 22.7, 22.5, 21.0, 19.3, 18.7, 11.8. $C_{43}H_{58}N_2O_5$ HRMS-ESI: $[M+Na]^+$ calcd. m/z = 705.4238 Da, obs. m/z = 705.4206 Da

(**CND2**) *4-piperazinyl -1,8-naphthalimide cholesteryl glycinate:* In a round bottom flask, 100 mg (0.142 mmol) of **G-III** was suspended in 2 mL of DMSO. 122 mg (1.420 mmol, 10 equiv) of piperazine was added to the reaction mixture and the reaction was heated at 100°C with stirring for three hours. The reaction was cooled to room temperature and diluted with water to give < 1% DMSO solution. The crude product was then extracted with three 50 mL washes of EtOAc. The crude product was purified on a silica column in

a gradient of 100% $CHCl_3$ to 5:1 $CHCl_3$:IPA in 1% triethylamine. The pure fractions were washed with 10% HCl to remove the triethylamine. The organic layer was collected and washed with a saturated solution of sodium bicarbonate followed by a wash with deionized water. The organic layer was collected and evaporated to obtain 56 mg of a yellow solid (yield = 55%). ^{1}H-NMR (400MHz $CDCl_3$): δ 8.60 (d, 1H), 8.54 (d, 1H), 8.45 (d, 1H), 7.72 (t, 1H), 7.24 (d, 1H), 5.38 (s, 1H), 4.92 (s, 2H), 4.73 (m, 1H), 3.23 (dt, 8H), 2.39 (d, 2H) 0.64 – 2.1 (m, 41H) ^{13}C-NMR (400 MHz $CDCl_3$): δ 167.6, 164.1, 163.6, 156.7,139.5, 132.9, 131.4, 130.7, 130.1, 126.2, 125.6, 122.8, 122.7, 116.1, 114.9, 75.2, 56.69, 56.1, 54.3, 49.9, 46.1, 42.3, 41.5, 39.7, 39.5, 37.9, 36.9, 36.5, 36.1, 35.7, 31.9, 31.8, 28.2, 28.0, 27.7, 24.2, 23.8, 22.8, 22.5, 21.0, 19.3, 18.7, 11.8. $C_{45}H_{61}N_3O_4$ HRMS-ESI: $[M+H]^+$ calcd. m/z = 708.4735 Da, obs. m/z = 708.4708 Da

(**CND5**) *4-morpholine-1,8-naphthalimide cholesteryl glycinate*: In a round bottom flask, 50 mg of **G-III** was dissolved in 2 mL of DMSO, stirred and heated to 100°C. To the heated solution, 62 μL (0.713 mmol, 10 equiv) of morpholine was added to the reaction and stirred for 4 hours. After the reaction, the solution was cooled to room temperature and diluted with 200mL of water. The aqueous layer was extracted with EtOAc (3x50mL) and collected. The organic layer was then evaporated to obtain a crude yellow solid. The crude product was purified in 20:1 DCM:MeOH on a silica column to yield a bright yellow solid 33 mg (yield = 65%). ^{1}H-NMR (400 MHz $CDCl_3$): δ 8.63 (d, 1H), 8.56 (d, 1H), 8.47 (d, 1H), 7.73 (t, 1H), 7.27 (d, 1H), 5.39 (d, 1H), 4.9 (s, 2H), 4.73 (m, 1H), 4.04 (t, 4H), 3.30 (t, 4H), 2.40- 0.69 (m, 43H). ^{13}C-NMR (400 MHz $CDCl_3$): δ 167.5, 164.0, 163.5, 155.9, 139.5, 132.9, 131.5, 130.4, 130.1, 126.2, 125.8, 122.9, 122.7, 116.7, 114.9, 75.3, 66.9, 56.7, 56.1, 53.4, 50.0, 42.3, 41.5, 39.7, 39.5, 37.9, 36.9, 36.5, 36.1, 35.7, 31.9, 31.8, 28.2, 28.0, 27.7, 24.2, 23.8, 22.8, 22.5, 21.0, 19.3, 18.7, 11.8. $C_{45}H_{60}N_2O_5$ HRMS-ESI: $[M+Na]^+$ calcd. m/z = 731.4394 Da, obs. m/z = 731.4361 Da

(**CND7**) *4-imidazole-1,8-naphthalimide-cholesteryl-glycinate*: 50 mg of **G-III**, 10mg of imidazole, 42 mg of Cs_2CO_3, 1.6 mg of metformin, and 4.9 mg of CuI were suspended in 1 mL DMF. The suspended reaction solution was stirred and heated to 110 °C for 24 hours. After the 24 hours, the reaction was cooled to room temperature and diluted in 100 mL of water and acidified. The aqueous solution was extracted with EtOAc (3x50mL) and the organic layer was collected and evaporated. The crude solid was purified in a gradient of 100% $CHCl_3$ to 99:1 $CHCl_3$:TEA to yield 26 mg of an off white/yellow solid (yield = 53%). ^{1}H-NMR (400 MHz $CDCl_3$): δ 8.73 (m, 2H), 8.13 (d, 1H), 7.88 (m, 2H), 7.77 (d, 1H), 7.40 (d 2H), 5.40 (s, 1H), 4.96 (s, 2H), 4.75 (m, 1H), 2.42 – 0.69 (m, 43H). ^{13}C-NMR (400 MHz $CDCl_3$): δ 167.2, 163.4, 162.9, 139.4, 139.4, 132.5, 131.4, 130.6, 129.3, 129.2, 128.4, 127.6, 124.1, 122.9, 122.7, 122.6, 75.6, 56.6, 56.1, 49.9, 42.3, 41.7, 39.7, 39.5, 37.9, 36.9, 36.5, 36.1, 35.7, 31.9, 31.8, 28.2, 28.0, 27.7, 24.2, 23.8, 22.8, 22.5, 21.0, 19.3, 18.7, 11.8. $C_{44}H_{55}N_3O_4$ HRMS-ESI: $[M+H]^+$ calcd. m/z = 690.4252 Da, obs. m/z = 690.4265 Da

(**CND8**) *4-hydroxy-1,8-naphthalimide-cholesteryl-glycinate*: 50 mg of **G-III** (0.071 mmol), 58 mg of *N*-Hydroxyphthalimide (5 equivalents), 59 mg of K_2CO_3 (5 equivalents) were suspended in 2 mL DMSO. The reaction was heated to 100 °C and spun for 3 hours. After the 3 hours, the reaction was cooled to room temperature, diluted with

200 mL water, and acidified with 10% HCl. The aqueous layer was extracted with EtOAc (3x25mL) and then it was evaporated. The crude solid was purified on a silica column in a gradient of 100% $CHCl_3$ to 9:1 $CHCl_3$:EtOAc to yield 29 mg of a yellow solid (yield = 64%). ^{1}H-NMR (400 MHz $CDCl_3$): δ 8.46, (d, 1H), 7.98 (d, 1H), 7.94 (d, 1H), 7.38 (t, 1H), 6.72 (d, 1H), 5.47 (s, 1H), 4.99 (s, 1H), 4.89 (m, 1H), 2.54 (d, 2H), 0.71 – 2.06 (m, 42H). ^{13}C-NMR (400 MHz $CDCl_3$): δ 170.8, 164.3, 163.3, 158.9, 139.2, 134.0, 131.5, 129.0, 125.4, 123.2, 122.0, 121.1, 113.3, 109.8, 56.7, 56.1, 50.0, 42.3, 41.4, 39.7, 39.5, 37.9, 36.9, 36.6, 36.1, 35.8, 31.9, 31.8, 28.2, 28.0, 27.7, 24.3, 22.8, 22.5, 21.0, 19.3, 18.7, 11.8. $C_{41}H_{53}NO_5$ HRMS-ESI: $[M+Na]^+$ calcd. m/z = 662.3816 Da, obs. m/z = 662.3783 Da

(**CND9**) *4-(4-hydroxy-piperadinyl)-1,8-naphthalimide-cholesteryl-glycinate*: 50 mg of **G-III** (0.071 mmol) and 36 mg of 4-hydroxypiperadine (5 equivalents) was dissolved in 1 mL DMSO, stirred, and heated to 100 °C for four hours. After the four hours, the reaction was cooled to room temperature and diluted with 100 mL of water. The aqueous layer was then extracted with EtOAc (3x25mL) and the organic layer was collected and evaporated. The crude solid was then purified on silica column in a gradient of 1:1 $CHCl_3$:hexanes to 9:1 $CHCl_3$:EtOAc to yield 42 mg of a yellow/orange solid (yield = 82%). ^{1}H-NMR (400 MHz $CDCl_3$): δ 8.60 (d, 1H), 8.52 (d, 1H), 8.41 (d, 1H), 7.72 (t, 1H), 7.24 (d, 1H), 5.38 (s, 1H), 4.92 (s, 2H), 4.73 (m, 1H), 4.04 (m, 1H), 3.53 (m, 2H), 3.10 (t, 2H), 0.68-2.39 (m, 48H). ^{13}C-NMR (400 MHz $CDCl_3$): δ 167.6, 164.2, 163.6, 156.7, 139.5, 132.9, 131.4, 130.7, 130.1, 126.3, 125.6, 122.8, 122.7, 115.9, 115.0, 75.2, 67.3, 56.6, 56.1, 50.7, 49.9, 42.3, 41.5, 39.7, 39.5, 37.9, 36.9, 36.5, 36.1, 35.7, 34.6, 31.9, 31.8, 28.2, 28.0, 27.7, 24.2, 23.8, 22.8, 22.5, 21.0, 19.3, 18.7, 11.8. $C_{46}H_{62}N_2O_5$ HRMS-ESI: $[M+H]^+$ calcd. m/z = 723.4731 Da, obs. m/z = 723.4707 Da

(**CND10**) *4-(4-carboxy-piperadinyl)-1,8-naphthalmide-choleseryl-glycinate*: 100 mg of **G-III** (0.142 mmol), 100 mg of tert-Butyl piperadine-4-carboxylate (3.8 equiv), was suspended in 3 mL of DMSO. The reaction was heated to 110 °C and ran for four hours and then cooled to room temperature. The reaction mixture was then diluted in 300 mL water and the aqueous layer was acidified. The aqueous layer was extracted with EtOAc (3x50 mL) and evaporated. The crude yellow solid was solubilized in 6 mL DCM and 80 μL TIS. The reaction mixture was spun at room temperature and then 2 mL of TFA was added to the reaction. The reaction ran for 2 hours and then the reaction was quenched with 30 mL H_2O and TFA was neutralized with saturated $NaHCO_3$. The reaction was then acidified with 10% HCl and then extracted with EtOAc (3x30mL). The organic layer was collected and evaporated to obtain a crude yellow solid. 64 mg of the crude yellow solid was purified in a gradient of 50:1 $CHCl_3$:IPA to 20:1 $CHCl_3$:IPA to obtain 36 mg of yellow solid. (yield = 56%). ^{1}H-NMR (400 MHz $CDCl_3$): δ 8.62 (d, 1H), 8.54 (d, 1H), 8.42 (d, 1H), 7.74 (t, 1H), 7.25 (d, 1H), 5.38 (s, 1H), 4.93 (s, 2H), 4.74 (m, 1H), 3.61 (d, 2H), 3.04 (t, 2H), 0.69 – 2.68 (m, 48H). ^{13}C-NMR (400 MHz $CDCl_3$): δ 179.3, 167.6, 164.1, 163.6, 156.7, 139.5, 132.9, 131.5, 130.6, 130.1, 126.4, 125.7, 122.8, 122.7, 116.2, 115.1, 75.3, 56.6, 56.1, 52.8, 49.9, 42.3, 41.5, 40.4, 39.7, 39.5, 37.9, 36.9, 36.5, 36.1, 35.8, 31.9, 31.8, 28.3, 28.0, 27.7, 24.2, 23.8, 22.8, 22.5, 21.0, 19.3, 18.7, 11.8. $C_{47}H_{62}N_2O_6$ HRMS-ESI: $[M+Na]^+$ calcd. m/z = 773.4500 Da, obs. m/z = 773.4467 Da

Synthesis of β-alanine Linker Analog: CND4

(**B-I**) *Cholesteryl-(Boc-β-alanine) ester:* In a round bottom flask, 100mg of cholesterol (0.259 mmol), 73.5 mg of *Boc-β-alanine* (0.388 mmol, 1.5 equivalents), and 5 mg of DMAP (0.0259 mmol) were dissolved in anhydrous DCM (10 mL). The solution was stirred and purged with N_2 and maintained at 0°C in an ice bath. 85 mg of DCC (0.414 mmol) was dissolved in 2 mL of anhydrous DCM and added to the reaction mixture slowly. The reaction was allowed to stir for 30 minutes on the ice bath before being removed and stirring at room temperature overnight. Dicyclohexylurea was filtered off and the filtrate was collected. The filtrate was washed three times with 20 mL of 10% HCl and the organic layer was dried over Na_2SO_4 and then was evaporated to give 86 mg of white crystals (yield = 60%) ^{1}H-NMR (400 MHz $CDCl_3$): δ 5.40 (s, 1H), 5.02 (s, 1H), 4.66 (m, 1H), 3.40 (q, 2H), 2.51 (t, 2H), 2.34 (d, 2H), 2.01 – 0.69 (m, 50H) ^{13}C-NMR (400 MHz $CDCl_3$): δ 171.9, 155.7, 139.5, 122.7, 79.3, 74.3, 56.7, 56.1, 50.0, 42.3, 39.7, 39.5, 38.1, 36.9, 36.6, 36.1, 35.7, 34.9, 31.9, 31.8, 28.4, 28.2, 28.0, 27.7, 24.2, 23.8, 22.8, 22.5. 21.0, 19.3, 18.7, 11.8. $C_{35}H_{59}NO_4$ HRMS-ESI: $[M+Na]^+$ calcd. m/z = 580.4336 Da, obs. m/z = 580.4347 Da

(**B-II**) *Cholesteryl-β-alanine ester:* In a round bottom flask, 86 mg **B-I** was dissolved in 10 mL DCM, and 3 mL TFA at room temperature. Reaction was stirred for 2 hours before being quenched with 50mL H_2O and saturated sodium bicarbonate to neutralize TFA. The product is then extracted with EtOAc and then organic layer is collected, and the organic solvent is evaporated. Attempts to purify βAla-Chol and NMR experiments fail due to the hydrolysis of the β-Alanine cholesterol ester to β-Alanine and cholesterol. After extraction step, the crude product is sufficient to perform the next reaction. The crude product is a wax-like substance. $C_{30}H_{51}NO_2$ HRMS-ESI: $[M+H]^+$ calcd. m/z = 458.3993 Da, obs. m/z = 458.4013 Da

(**B--III**) *4-bromo-1,8-naphthalimide-cholesteryl-β-alanine*: In a round bottom flask, **B-II** and 72 mg (1 equivalent) of 4-bromo-1,8-naphthalic anhydride was dissolved in ethanol and refluxed overnight. After reaction is complete, ethanol is removed by vacuum. The solid was purified on silica gel on 10:10:1 $CHCl_3$:Hx:EtOAc (v:v:v) to obtain 117 mg of a white solid (yield = 63%) ^{1}H-NMR (400MHz $CDCl_3$): δ 8.68, (d, 1H), 8.59 (d, 1H), 8.43 (d, 1H), 8.06 (d, 1H), 7.86 (t, 1H), 5.36 (s, 1H), 4.63 (m, 1H), 4.50 (t, 2H), 2.76 (t, 2H), 0.50-2.50 (m, 43H). ^{13}C-NMR (400Mhz $CDCl_3$): δ 170.5, 163.3, 139.6, 133.4, 132.1, 131.3, 131.1, 130.6, 130.4, 129.0, 128.0, 122.9, 122.6, 122.1, 74.3, 56.6, 56.1, 50.0, 42.3, 39.7, 39.5, 38.0, 36.9, 36.5, 36.3, 36.1, 35.7, 32.9, 31.9, 31.8, 28.2, 28.0, 27.6, 24.2, 23.8, 22.8, 22.5, 21.0, 19.2, 18.7, 11.8. $C_{42}H_{54}BrNO_4$ HRMS-ESI: $[M+Na]^+$ calcd. m/z = 740.3117 Da obs. m/z = 740.3097 Da

(**CND4**) *4-piperizinyl-1,8-naphthalimide-cholesteryl-β-alanate* 117mg of **B-III** was dissolved in 4mL of DMSO and 100mg of piperazine (7 equivalents, 1.148mmol), was heated to 100° C for 1 hour. After 1 hour the reaction was diluted in H_2O and extracted with DCM. DCM was evaporated and the crude was purified on silica gel in 7:3 DCM:IPA 0.2% triethylamine. The organic layer was washed with 10% HCl to remove triethylamine. The organic layer was then washed with saturated sodium bicarbonate, followed by a wash with deionized H_2O to obtain 50 mg of a yellow solid (yield = 42%). ^{1}H-NMR (400MHz $CDCl_3$): δ 8.61 (d,1H), 8.55 (d, 1H), 8.44 (d, 1H), 7.71 (t, 1H), 7.25 (d, 1H), 5.36 (s, 1H),

4.64 (m, 1H), 4.49 (t, 2H), 3.25 (dt, 8H), 2.75 (t, 2H), 2.3 – 0.5 (m, 43H). ^{13}C-NMR (400Mhz $CDCl_3$): δ 170.7, 164.2, 163.7, 156.3, 139.7, 132.6, 131.2, 130.3, 129.9, 126.2, 125.7, 123.1, 122.5, 116.6, 115.0, 74.2, 56.6, 56.1, 54.1, 50.0, 46.0, 42.3, 39.7, 39.5, 38.0, 36.9, 36.5, 36.1, 36.0, 35.7, 33.0, 31.9, 31.8, 28.2, 28.0, 27.6, 24.2, 23.8, 22.8, 22.5, 21.0, 19.2, 18.7, 11.8. $C_{46}H_{63}N_3O_4$ HRMS-ESI: $[M+H]^+$ calcd. m/z = 722.4891 Da, obs. m/z = 722.4878 Da

Synthesis of Serine Linker Analogs: CND3 and CND6

(**S-I**) *4-bromo-1,8-naphthalimide-(O-tertbutyl)-L-serine:* In a round bottom flask, 100 mg (0.361 mmol) of 4-bromo-1,8-naphthalic anhydride and 70 mg (1.2 equivalents, 0.433mmol) of O-tert-butyl-L-Serine was dissolved in 10mL of ethanol refluxed overnight. The reaction solution was concentrated on the rotavapor and then diluted in water and acidified (pH = 1). The aqueous solution was then extracted with EtOAc (3x 50mL) and the organic layer was collected and evaporated. The crude brown solid was purified on a silica column in 20:1 DCM:MeOH (v:v) and dried on sodium sulfate to yield 130 mg of a white solid (yield = 86%). ^{1}H-NMR (400MHz $CDCl_3$): δ 8.69 (d, 1H), 8.63 (d, 1H), 8.45 (d, 1H) 8.08 (d, 1H), 7.88 (t, 1H), 5.92 (t, 1H), 4.36 (t, 1H), 3.88 (t, 1H), 1.25 (s, 9H) ^{13}C-NMR (400MHz $CDCl_3$): δ 169.8, 163.2, 133.7, 132.5, 131.7, 131.2, 130.8, 130.7, 129.2, 128.1, 122.6, 121.7, 75.6, 70.5, 59.1, 52.5, 29.6, 27.3. $C_{19}H_{18}BrNO_5$ HRMS-ESI: $[M+Na]^+$ calcd. m/z = 444.0243 Da obs. m/z = 444.0263 Da

(**S-II**) *4-bromo-1,8-naphthalimide-cholesteryl-O-terbutyl-L-serine:* In a round bottom flask, 210 mg of cholesterol (0.542 mmol, 1.5 equivalents) 5 mg of DMAP (0.1 equivalents, 0.036 mmol) and **S-I** were dissolved in 10 mL of anhydrous DCM at 0° C under an N_2 atmosphere. Subsequently, 120 mg of DCC (1.6 equivalents, .577mmol) dissolved in 3mL of anhydrous DCM was added to the reaction. The reaction proceeded overnight and dicyclohexaurea was filtered off and washed with DCM while the filtrate was collected. The filtrate was extracted with 25 mL of 10% HCl and then subsequently by deionized H_2O. The organic layer was evaporated and then was purified on silica gel in 10:10:1 $CHCl_3$:hexanes:EtOAc (v:v:v) to obtain 180 mg of white crystals (yield = 63%). ^{1}H-NMR (400 MHz $CDCl_3$): δ 8.69 (d, 1H), 8.62 (d, 1H), 8.45 (d, 1H), 8.08 (d, 1H), 7.88 (t, 1H), 5.90 (q, 1H), 5.38 (s, 1H), 4.75 (m, 1H), 4.20 (q, 1H), 4.07 (t, 1H), 0.6 - 2.5 (m, 52H). ^{13}C-NMR (400 MHz $CDCl_3$): δ 167.8, 163.2, 139.5, 133.4, 132.3, 131.4, 131.1, 130.7, 130.4, 129.2, 128.1, 122.9, 122.7, 122.0, 75.0, 75.0, 73.3, 59.4, 56.6, 56.1, 54.2, 49.9, 42.3, 39.7, 39.5, 37.9, 37.8, 36.9, 36.8, 36.5, 36.1, 35.7, 31.8, 31.8, 28.2, 28.0, 27.5, 27.4, 24.2, 23.8, 22.8, 22.5, 21.0, 19.2, 18.7, 11.8. $C_{46}H_{62}BrNO_5$ HRMS-ESI: $[M+Na]^+$ calcd. m/z = 812.3694 Da, obs. m/z = 812.3709 Da

*(***CND3***) 4-piperazynl-1,8-naphthalimide-Cholesteryl-L-serinate:* 180 mg of **S-II** was suspended in 8 mL of DMSO and was stirred with 138 mg of piperazine (1.603mmol, 7 equivalents) and heated at 100°C for 1 hour. After 1 hour, the orange reaction solution was diluted in H_2O (< 1% DMSO) and extracted with DCM (3x100mL). The organic layer was collected and evaporated to obtain the crude 164 mg of yellow/orange solid. 164 mg of crude product was dissolved in 10mL DCM and 70 μL of triisopropylsilane (TIS) and stirred. To the stirred solution, 2mL of TFA was added and stirred at room temperature for 1 hour. After 1 hour, the reaction was diluted in 50mL H_2O and TFA was quenched

with a solution of saturated sodium bicarbonate. The organic layer was collected and evaporated. The crude product was purified on a silica column in 7:3 DCM:IPA 0.2% triethylamine. The organic layer was washed with 10% HCl to remove triethylamine. The organic layer was then washed with saturated sodium bicarbonate, followed by a wash with deionized H_2O to obtain 42 mg of an orange/yellow solid (yield = 25%). ^{1}H-NMR (400MHz $CDCl_3$): δ 8.61 (d, 1H), 8.53 (d, 1H), 8.45 (d, 1H), 7.73 (t, 1H), 7.23 (d, 1H), 5.79 (t, 1H), 5.39 (s, 1H) 4.78 (m, 1H), 4.42 (q, 1H), 4.00 (q, 1H), 3.66 (s, 1H), 2.40- 0.69 (m, 43H).^{13}C-NMR (400Mhz $CDCl_3$): δ 169.1, 164.2, 163.7, 156.8, 139.4, 133.2, 131.6, 130.8, 130.1, 126.2, 125.7, 122.8, 116.0, 115.0, 75.2, 70.5, 61.1, 56.6, 56.1, 54.5, 54.3, 49.9, 46.1, 42.3, 39.7, 39.5, 37.9, 37.8, 36.9, 36.8, 36.5, 36.1, 35.7, 31.8, 31.8, 28.2, 28.0, 27.6, 27.5, 24.2, 23.8, 22.8, 22.5, 21.0, 19.2, 18.7, 11.8. $C_{46}H_{63}N_3O_5$ HRMS-ESI: $[M+H]^+$ calcd. m/z = 738.484 Da, obs. m/z = 738.4837 Da

(**CND6**) *4-Morpholinyl-1,8-napthalimide Cholesteryl Serinate*: In a round bottom flask, 34 mg of **S-II** was suspended in 1 mL of DMSO. To the reaction mixture, 75 μL (20 equivalents, 0.0864 mmol) of morpholine was added and the reaction mixture was heated to 100°C for 3 hours. After the 3 hours, the reaction was cooled to room temperature and diluted in 100 mL of H_2O and acidified with 10% HCl in water (v:v). The aqueous layer was then extracted with EtOAc (3x25mL) and collected. The crude yellow solid was resolubilized in 5 mL DCM and stirred. To the stirred solution, 1 mL TFA and 35 μL of TIS was added at room temperature. After 1 hour, the reaction was diluted in 25mL H_2O and TFA was quenched with a solution of saturated sodium bicarbonate. The organic layer was collected, evaporated, and purified on a silica column in a gradient of 10:1 DCM:EtOAC, and 4:1 DCM:EtOAc. The organic layer was evaporated to a bright yellow solid with a yield of 18 mg (yield = 55%). ^{1}H-NMR (400MHz $CDCl_3$): δ 8.62 (d, 1H), 8.57 (d, 1H), 8.48 (d, 1H), 7.74 (t, 1H), 7.27 (d, 1H), 5.79 (t, 1H), 5.39 (s, 1H), 4.78 (m, 1H) 4.42 (q, 1H), 4.04 (t, 4H), 4.00 (m, 1H), 3.30 (t, 4H), 2.93 (s, 1H), 2.40- 0.69 (m, 43H). ^{13}C-NMR (400MHz $CDCl_3$): δ 169.0, 164.1, 163.6, 156.1, 139.4, 133.1, 130.6, 131.7, 130.1, 126.1, 125.9, 122.8, 122.8, 116.5, 115.0, 75.3, 66.9, 61.1, 56.6, 56.1, 54.5, 53.4, 49.9, 42.2, 39.7, 39.5, 37.9, 36.8, 36.5, 36.1, 35.7, 31.8, 31.8, 28.2, 28.0, 27.5, 24.2, 23.8, 22.8, 22.5, 21.0, 19.2, 18.7, 11.8. $C_{46}H_{62}N_2O_6$, HRMS-ESI: $[M+Na]^+$ calcd. m/z = 761.45 Da, obs. m/z = 761.4478 Da

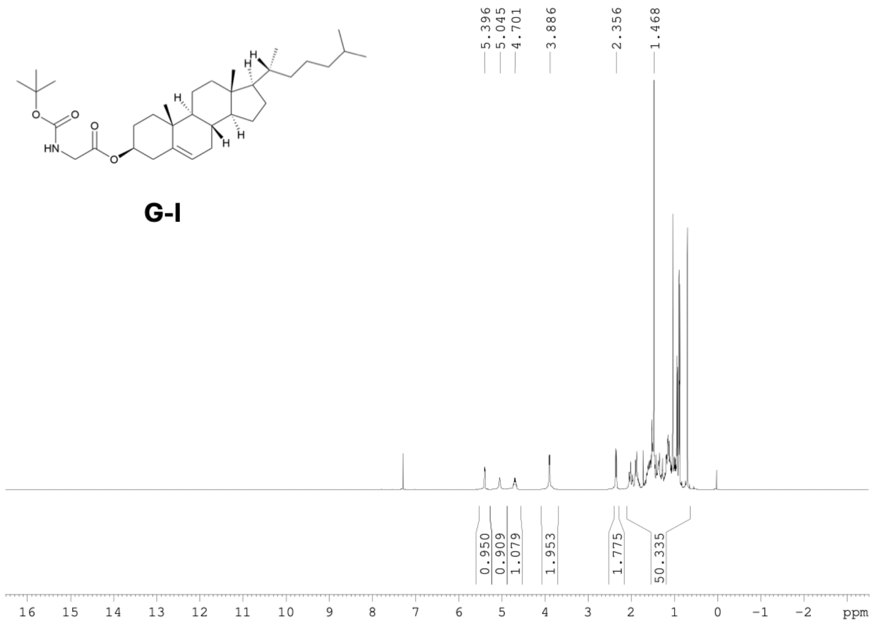
G-I
5.396
5.045
4.701
3.886
2.356
1.468
0.950
0.909
1.079
1.953
1.775
50.335
16 15 14 13 12 11 10 9 8 7 6 5 4 3 2 1 0 -1 -2 ppm

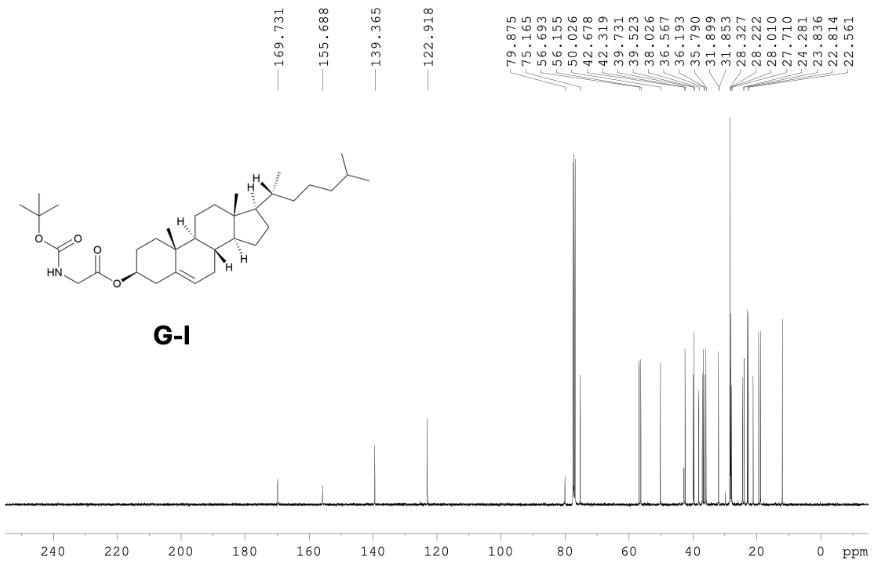
G-I
169.731
155.688
139.365
122.918
79.875
75.165
56.693
56.155
50.026
42.678
42.319
39.731
39.523
38.026
36.567
36.193
35.790
31.899
31.853
28.327
28.222
28.010
27.710
24.281
23.836
22.814
22.561
240 220 200 180 160 140 120 100 80 60 40 20 0 ppm

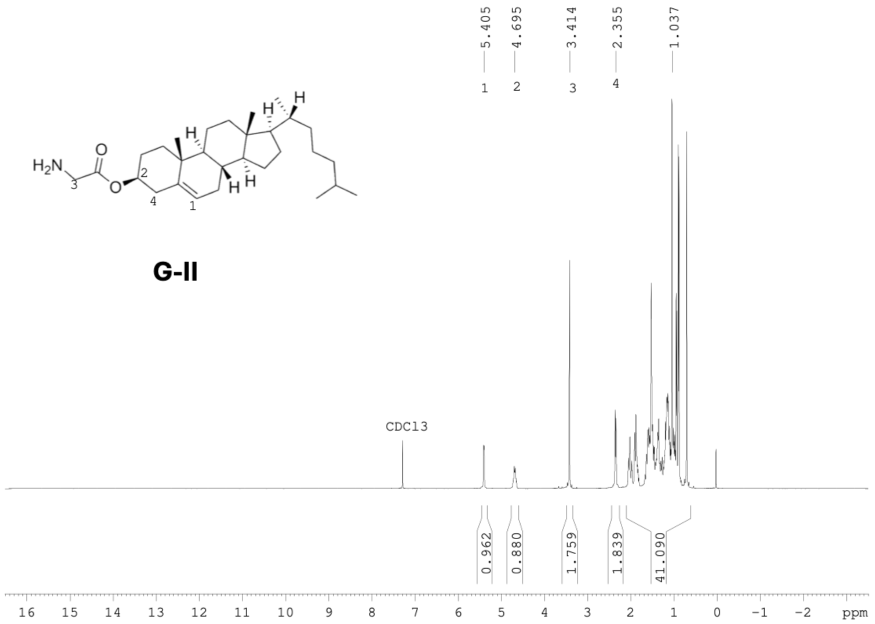
5.405
4.695
3.414
2.355
1.037
1 2 3 4
G-II
CDCl3
0.962
0.880
1.759
1.839
41.090
16 15 14 13 12 11 10 9 8 7 6 5 4 3 2 1 0 -1 -2 ppm

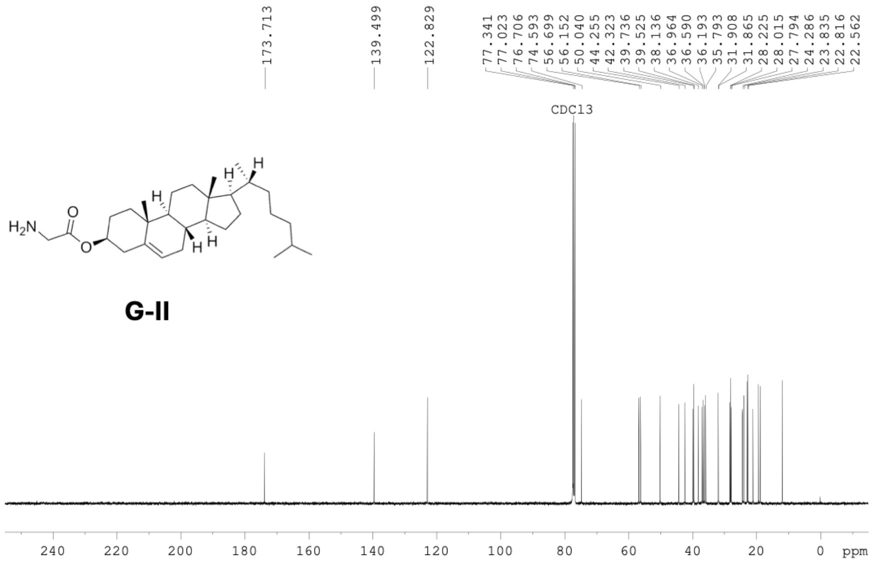
173.713
139.499
122.829
77.341
77.023
76.706
74.593
56.699
56.152
50.040
44.255
42.323
39.736
39.525
38.136
36.964
36.590
36.193
35.793
31.908
31.865
28.225
28.015
27.794
24.286
23.835
22.816
22.562
CDCl3
G-II
240 220 200 180 160 140 120 100 80 60 40 20 0 ppm

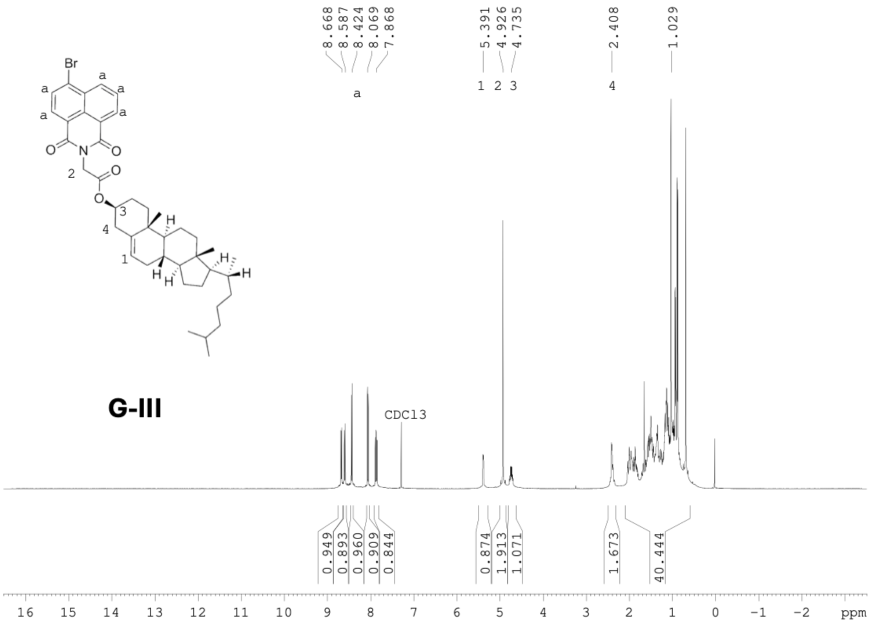

8.668
8.587
8.424
8.069
7.868
5.391
4.926
4.735
2.408
1.029
a
1 2 3
4
Br
G-III
CDCl3
0.949
0.893
0.960
0.909
0.844
0.874
1.913
1.071
1.673
40.444
ppm

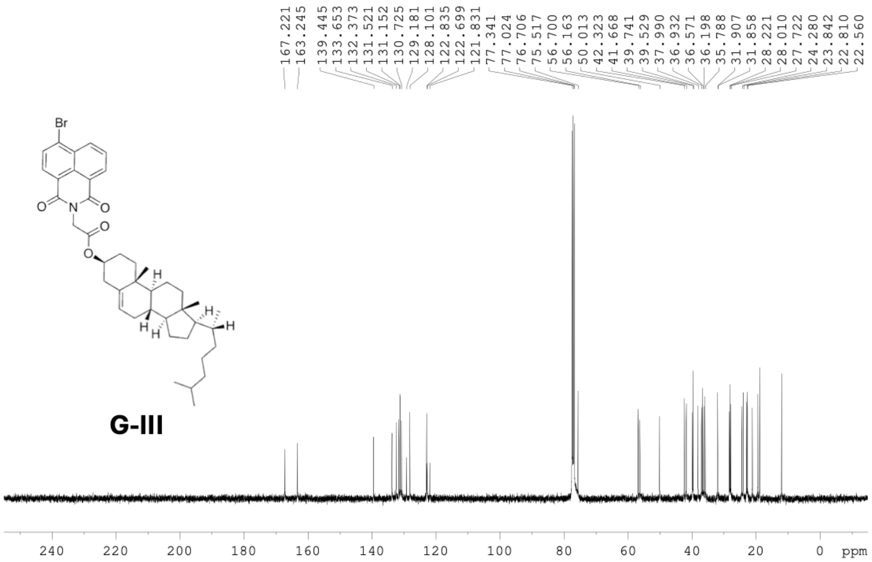

167.221
163.245
139.445
133.653
132.373
131.521
131.152
130.725
129.181
128.101
122.835
122.699
121.831
77.341
77.024
76.706
75.517
56.700
56.163
50.013
42.323
41.668
39.741
39.529
37.990
36.932
36.571
36.198
35.788
31.907
31.858
28.221
28.010
27.722
24.280
23.842
22.810
22.560
Br
G-III
ppm

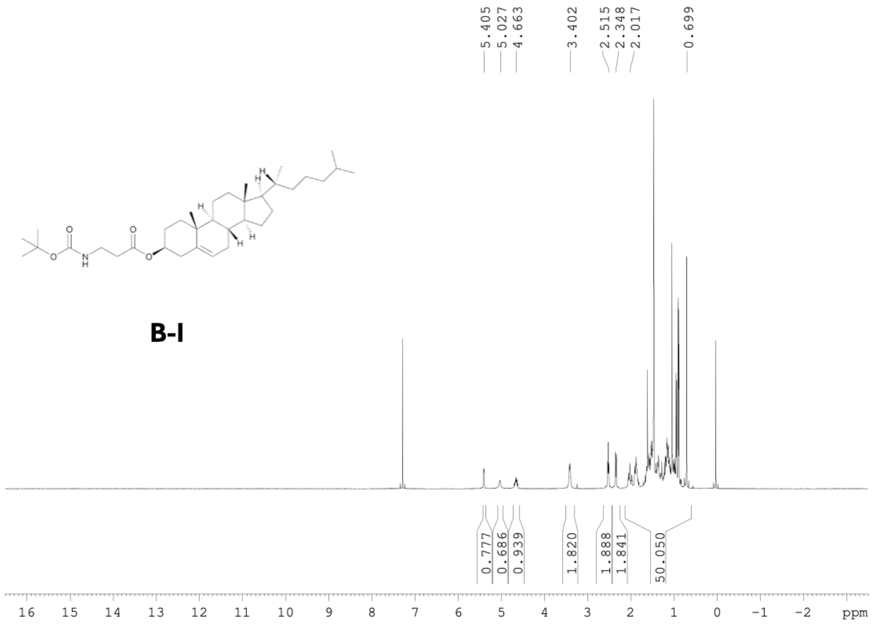
5.405
5.027
4.663
3.402
2.515
2.348
2.017
0.699
B-I
0.777
0.686
0.939
1.820
1.888
1.841
50.050
16 15 14 13 12 11 10 9 8 7 6 5 4 3 2 1 0 -1 -2 ppm

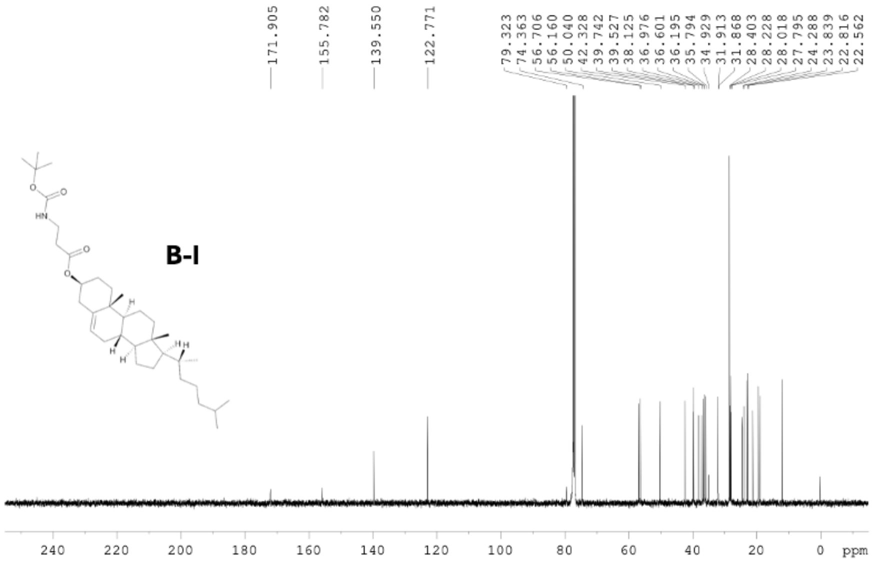
171.905
155.782
139.550
122.771
79.323
74.363
56.706
56.160
50.040
42.328
39.742
39.527
38.125
36.976
36.601
36.195
35.794
34.929
31.913
31.868
28.403
28.228
28.018
27.795
24.288
23.839
22.816
22.562
B-I
240 220 200 180 160 140 120 100 80 60 40 20 0 ppm

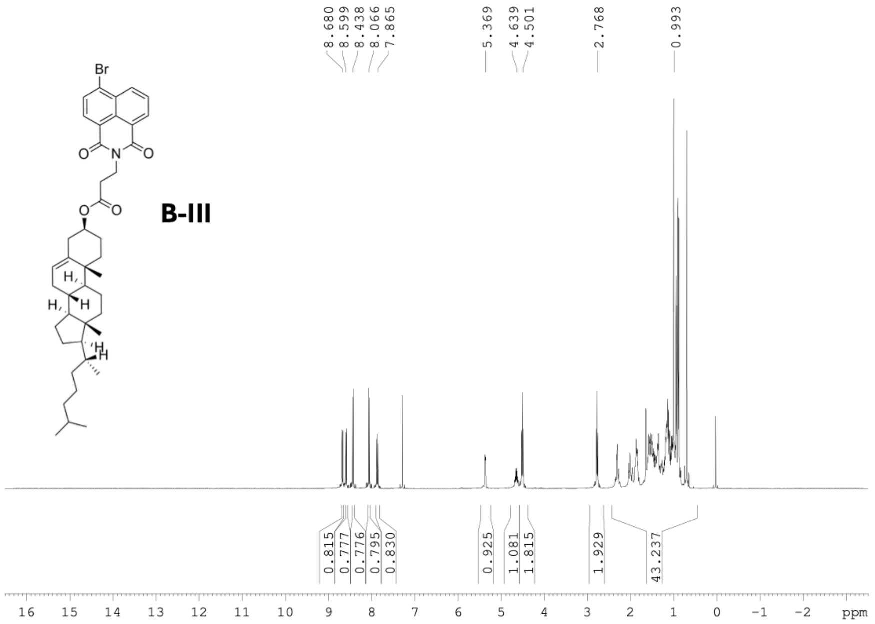
Br
O
N
O
O
O
B-III
H
H
H
H
8.680
8.599
8.438
8.066
7.865
5.369
4.639
4.501
2.768
0.993
0.815
0.777
0.776
0.795
0.830
0.925
1.081
1.815
1.929
43.237
16 15 14 13 12 11 10 9 8 7 6 5 4 3 2 1 0 −1 −2 ppm

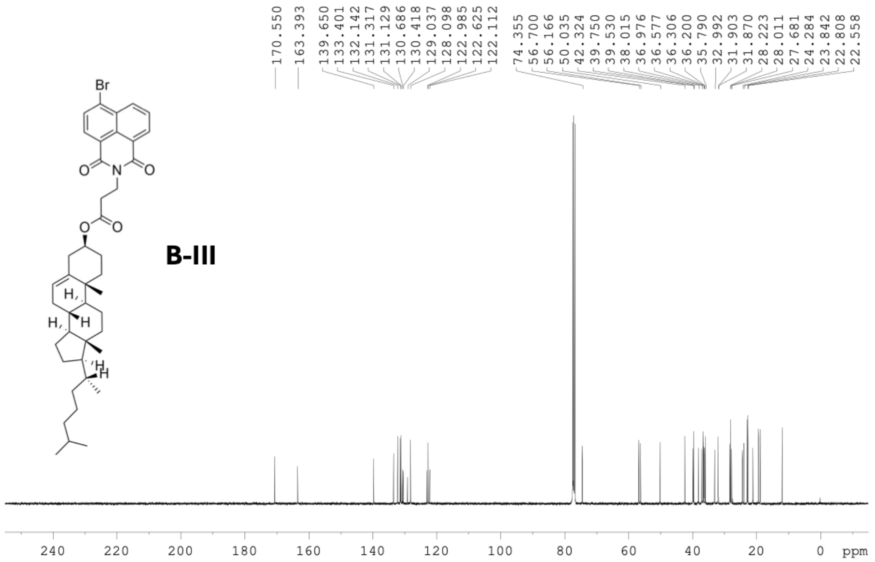
Br
O
N
O
O
O
B-III
H
H
H
H
170.550
163.393
139.650
133.401
132.142
131.317
131.129
130.686
130.418
129.037
128.098
122.985
122.625
122.112
74.355
56.700
56.166
50.035
42.324
39.750
39.530
38.015
36.976
36.577
36.306
36.200
35.790
32.992
31.903
31.870
28.223
28.011
27.681
24.284
23.842
22.808
22.558
240 220 200 180 160 140 120 100 80 60 40 20 0 ppm

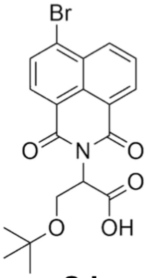

S-I

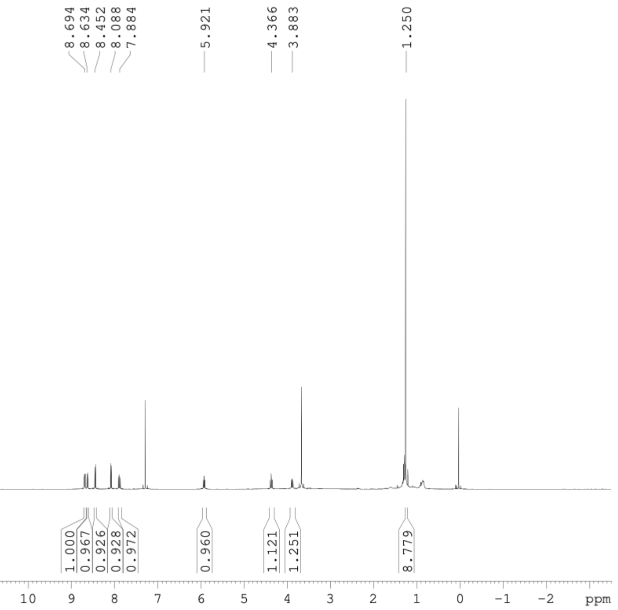
8.694
8.634
8.452
8.088
7.884
5.921
4.366
3.883
1.250
1.000
0.967
0.926
0.928
0.972
0.960
1.121
1.251
8.779
16 15 14 13 12 11 10 9 8 7 6 5 4 3 2 1 0 -1 -2 ppm

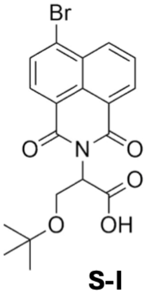

S-I

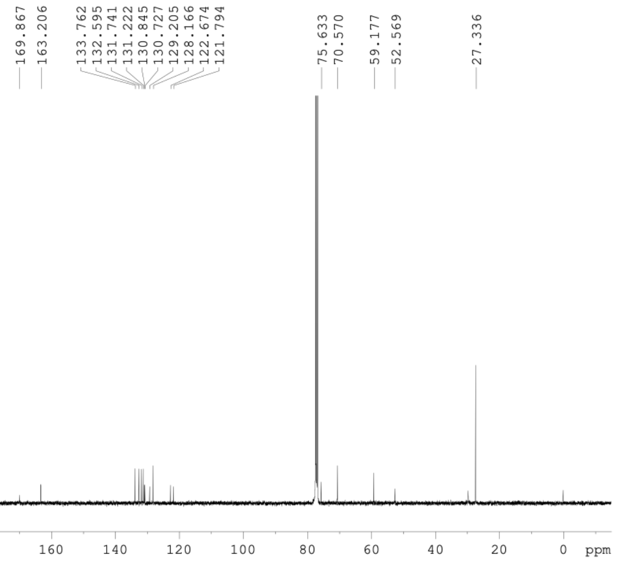
169.867
163.206
133.762
132.595
131.741
131.222
130.845
130.727
129.205
128.166
122.674
121.794
75.633
70.570
59.177
52.569
27.336
240 220 200 180 160 140 120 100 80 60 40 20 0 ppm

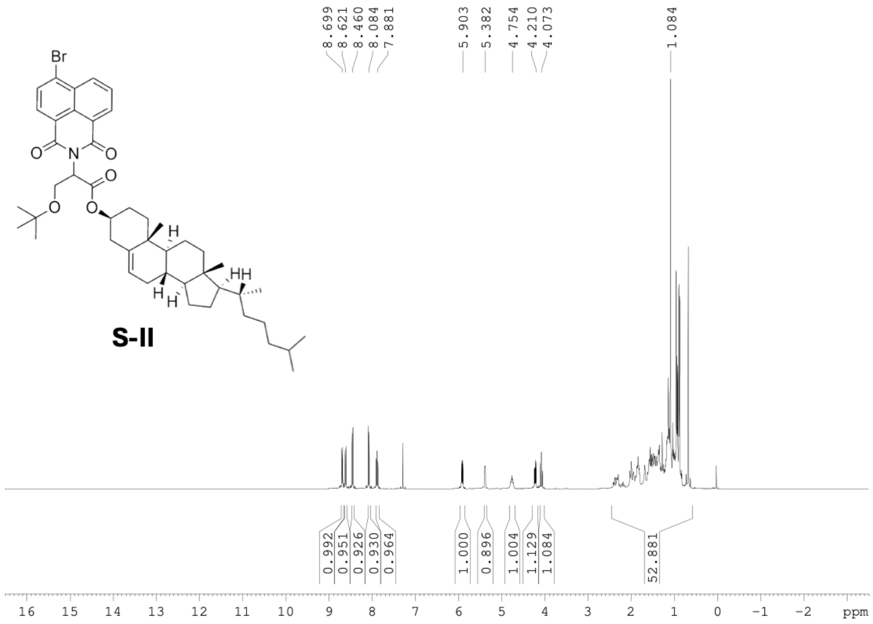

8.699
8.621
8.460
8.084
7.881
5.903
5.382
4.754
4.210
4.073
1.084
Br
O
N
O
O
O
O
H
H
H
H
H
S-II
0.992
0.951
0.926
0.930
0.964
1.000
0.896
1.004
1.129
1.084
52.881
ppm

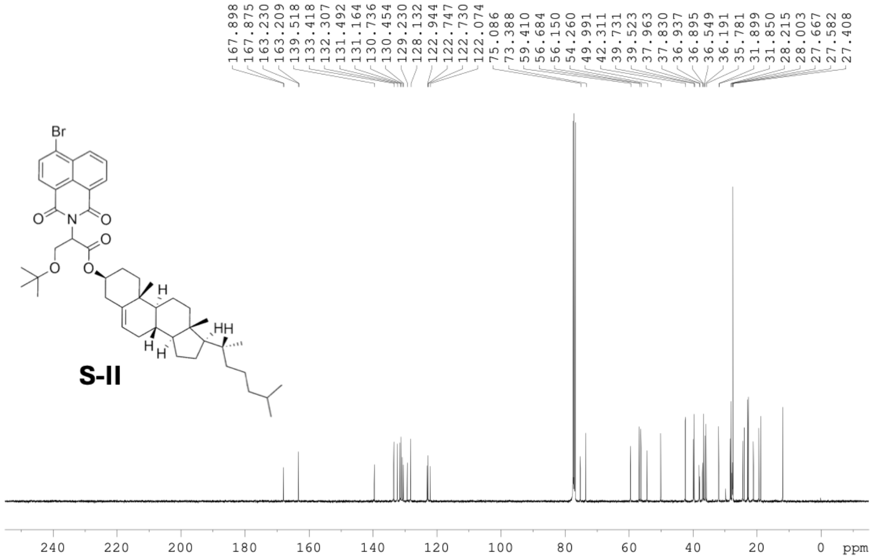

167.898
167.875
163.230
163.209
139.518
133.418
132.307
131.492
131.164
130.736
130.454
129.230
128.132
122.944
122.747
122.730
122.074
75.086
73.388
59.410
56.684
56.150
54.260
49.991
42.311
39.731
39.523
37.963
37.830
36.937
36.895
36.549
36.191
35.781
31.899
31.850
28.215
28.003
27.667
27.582
27.408
Br
O
N
O
O
O
O
H
H
H
H
S-II
ppm

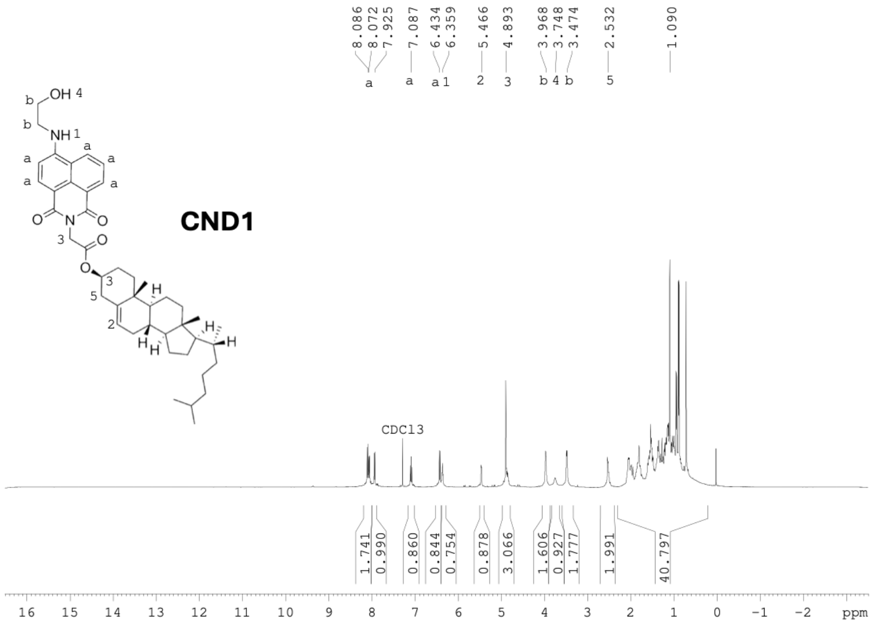
CND1
CDCl3
ppm

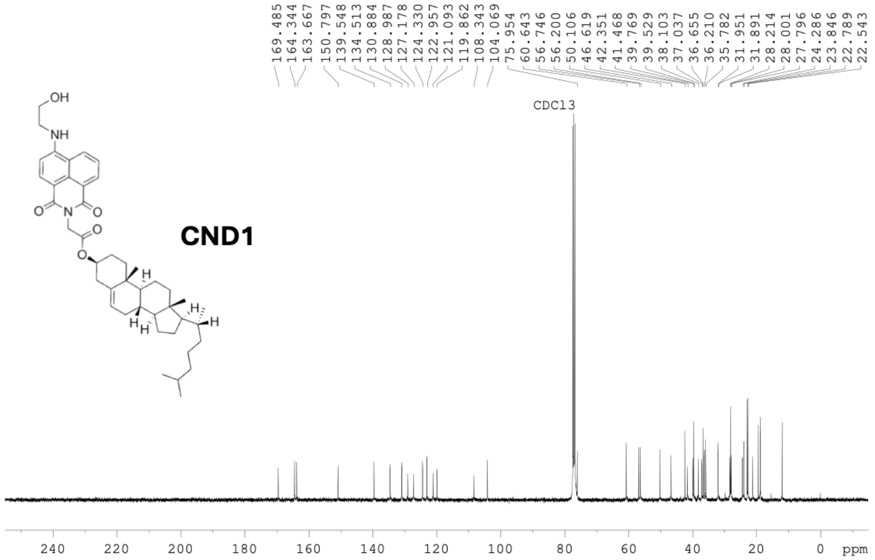
CND1
CDCl3
ppm

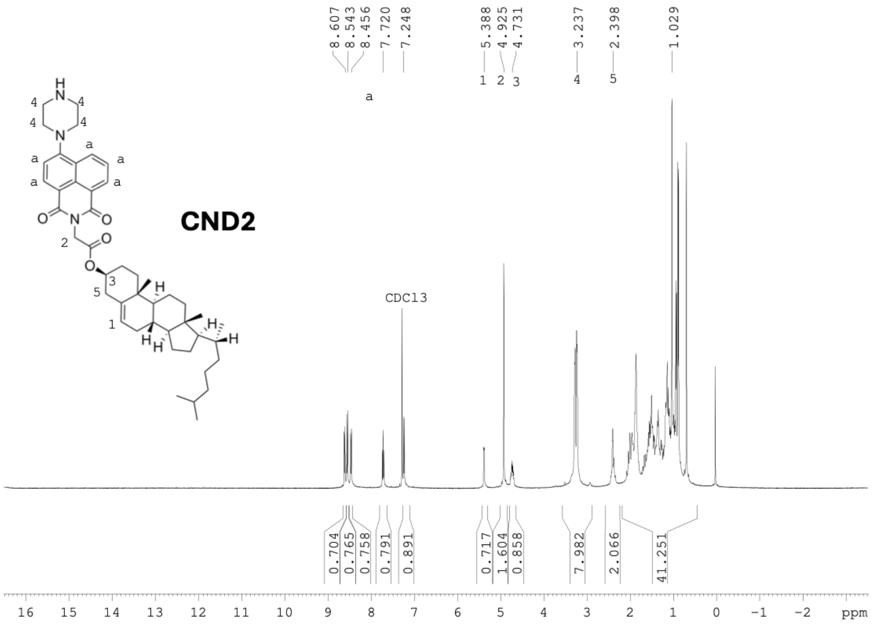
CND2
8.607
8.543
8.456
7.720
7.248
5.388
4.925
4.731
3.237
2.398
1.029
CDCl3
0.704
0.765
0.758
0.791
0.891
0.717
1.604
0.858
7.982
2.066
41.251
ppm

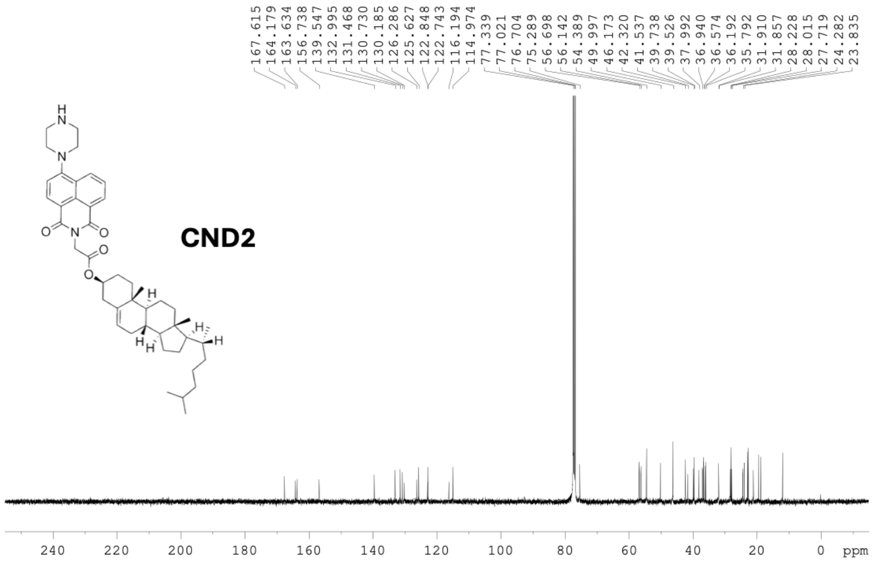
CND2
167.615
164.179
163.634
156.738
139.547
132.995
131.468
130.730
130.185
126.286
125.627
122.848
122.743
116.194
114.974
77.339
77.021
76.704
75.289
56.698
56.142
54.389
49.997
46.173
42.320
41.537
39.738
39.526
37.992
36.940
36.574
36.192
35.792
31.910
31.857
28.228
28.015
27.719
24.282
23.835
ppm

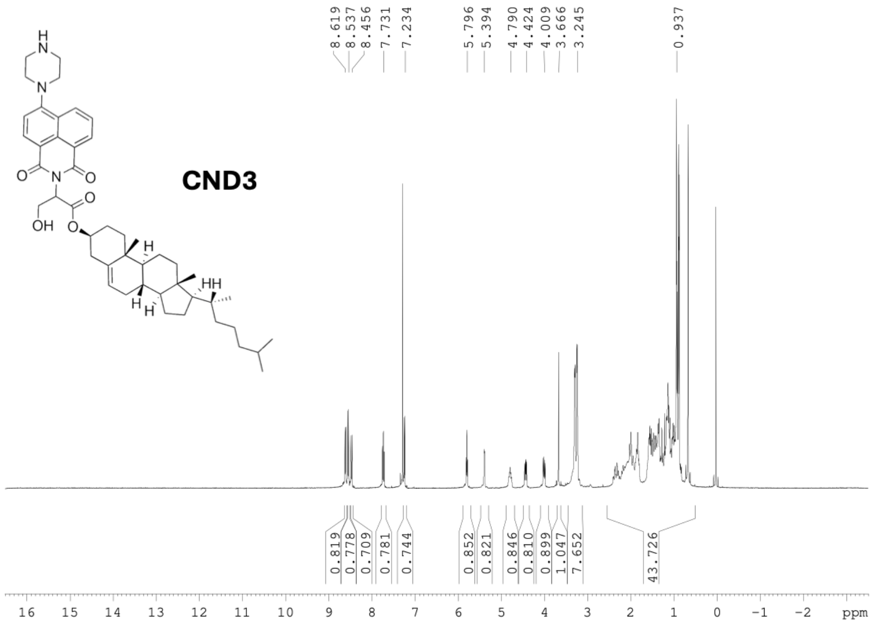
CND3
8.619
8.537
8.456
7.731
7.234
5.796
5.394
4.790
4.424
4.009
3.666
3.245
0.937
0.819
0.778
0.709
0.781
0.744
0.852
0.821
0.846
0.810
0.899
1.047
7.652
43.726
16 15 14 13 12 11 10 9 8 7 6 5 4 3 2 1 0 -1 -2 ppm

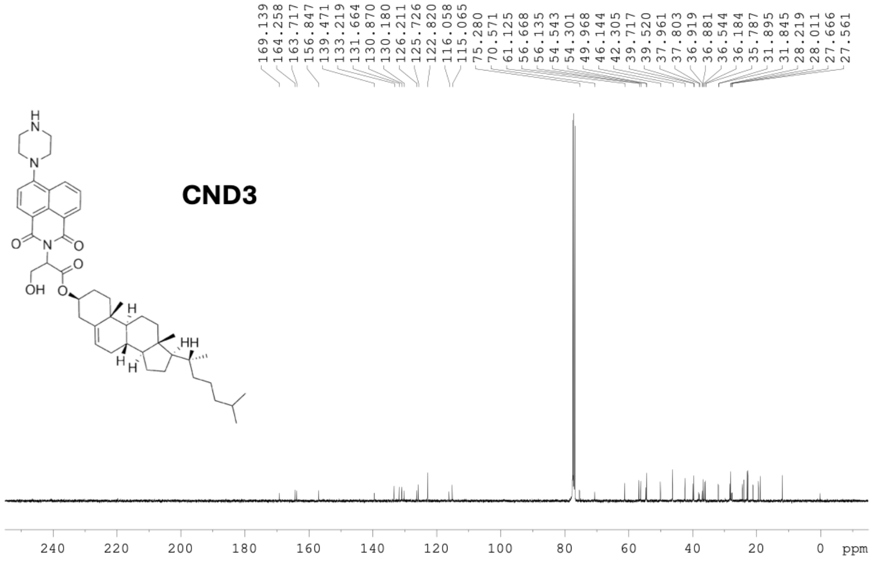
CND3
169.139
164.258
163.717
156.847
139.471
133.219
131.664
130.870
130.180
126.211
125.726
122.820
116.058
115.065
75.280
70.571
61.125
56.668
56.135
54.543
54.301
49.968
46.144
42.305
39.717
39.520
37.961
37.803
36.919
36.881
36.544
36.184
35.787
31.895
31.845
28.219
28.011
27.666
27.561
240 220 200 180 160 140 120 100 80 60 40 20 0 ppm

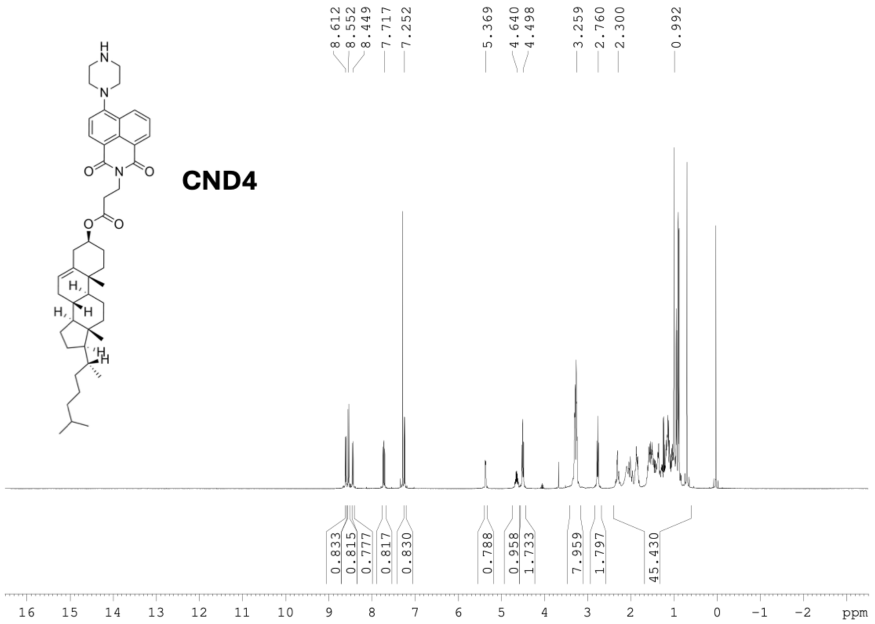
CND4
8.612
8.552
8.449
7.717
7.252
5.369
4.640
4.498
3.259
2.760
2.300
0.992
0.833
0.815
0.777
0.817
0.830
0.788
0.958
1.733
7.959
1.797
45.430
16 15 14 13 12 11 10 9 8 7 6 5 4 3 2 1 0 −1 −2 ppm

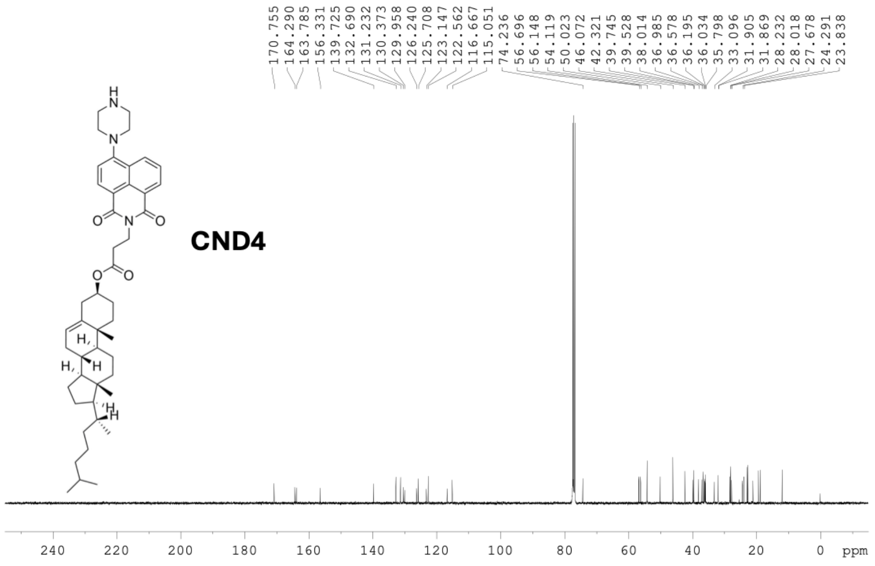
CND4
170.755
164.290
163.785
156.331
139.725
132.690
131.232
130.373
129.958
126.240
125.708
123.147
122.562
116.667
115.051
74.236
56.696
56.148
54.119
50.023
46.072
42.321
39.745
39.528
38.014
36.985
36.578
36.195
36.034
35.798
33.096
31.905
31.869
28.232
28.018
27.678
24.291
23.838
240 220 200 180 160 140 120 100 80 60 40 20 0 ppm

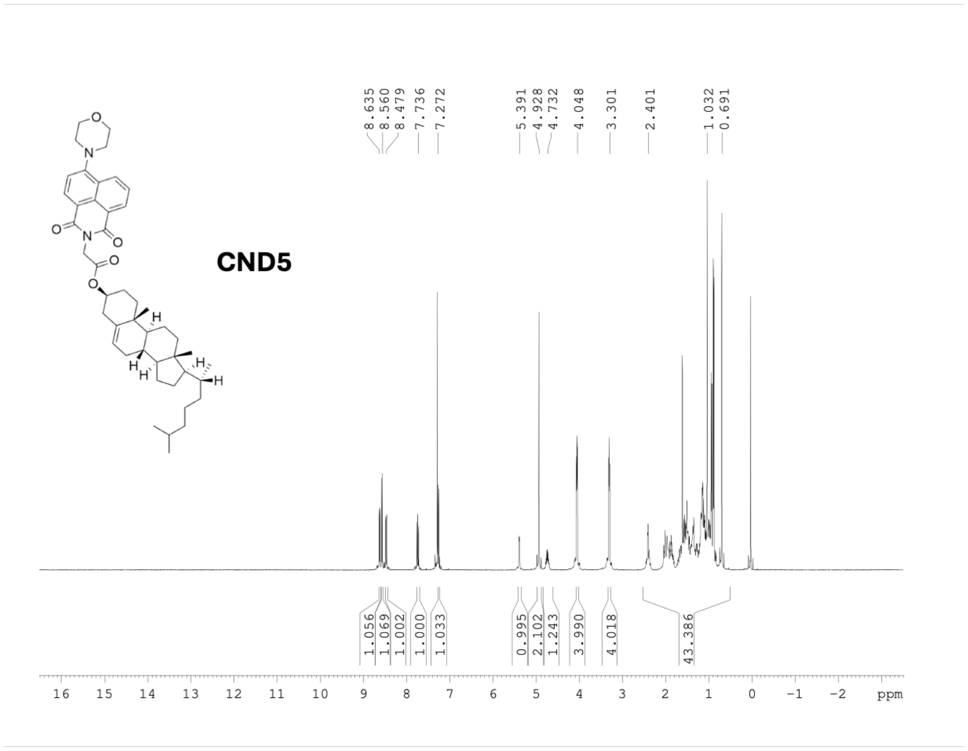
CND5
8.635
8.560
8.479
7.736
7.272
5.391
4.928
4.732
4.048
3.301
2.401
1.032
0.691
1.056
1.069
1.002
1.000
1.033
0.995
2.102
1.243
3.990
4.018
43.386
ppm

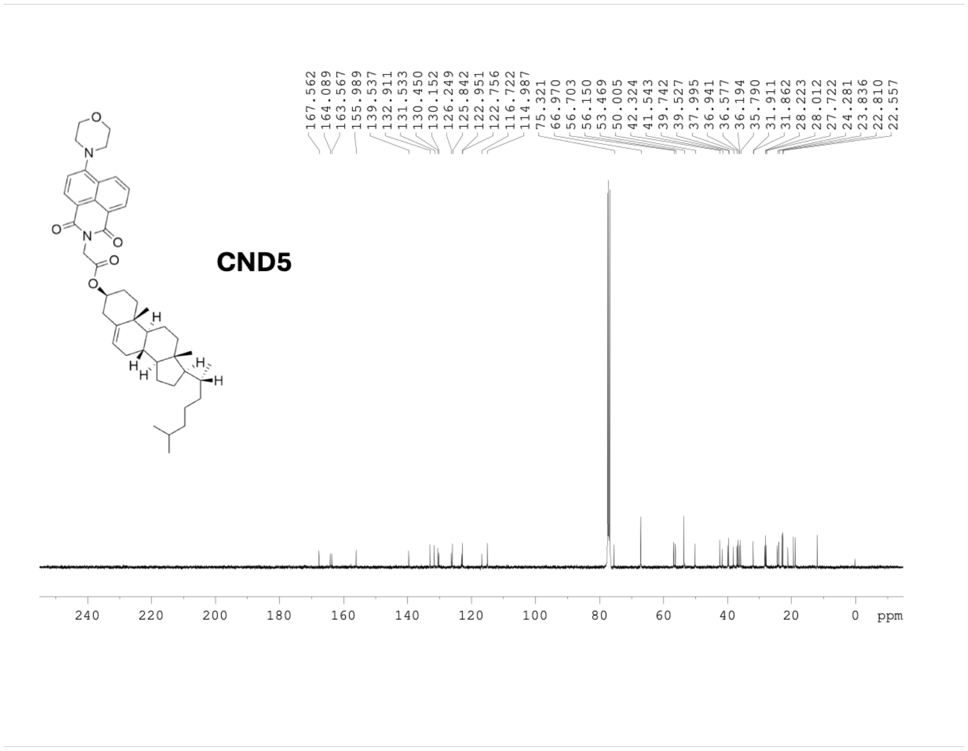
CND5
167.562
164.089
163.567
155.989
139.537
132.911
131.533
130.450
130.152
126.249
125.842
122.951
122.756
116.722
114.987
75.321
66.970
56.703
56.150
53.469
50.005
42.324
41.543
39.742
39.527
37.995
36.941
36.577
36.194
35.790
31.911
31.862
28.223
28.012
27.722
24.281
23.836
22.810
22.557
ppm

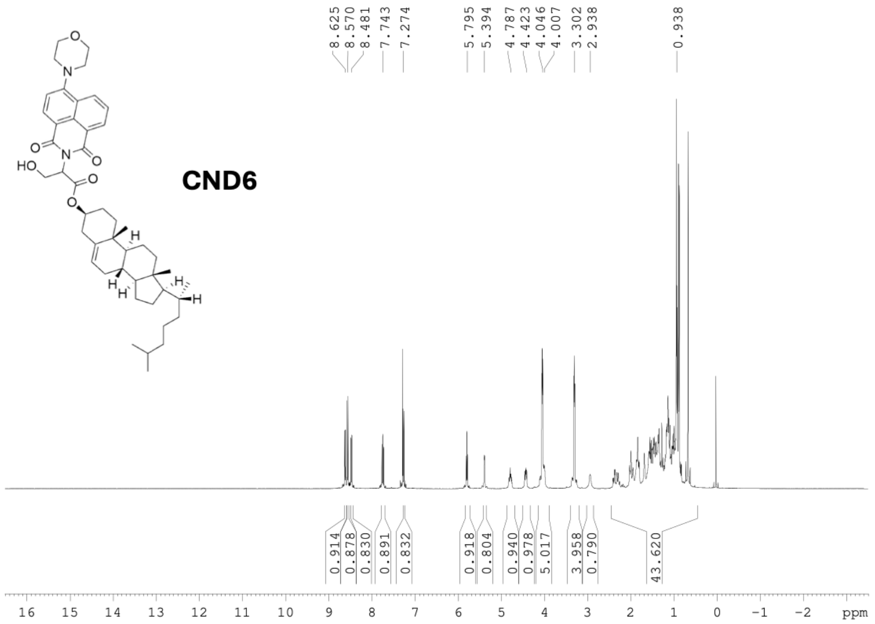
CND6
8.625
8.570
8.481
7.743
7.274
5.795
5.394
4.787
4.423
4.046
4.007
3.302
2.938
0.938
0.914
0.878
0.830
0.891
0.832
0.918
0.804
0.940
0.978
5.017
3.958
0.790
43.620
16 15 14 13 12 11 10 9 8 7 6 5 4 3 2 1 0 -1 -2 ppm

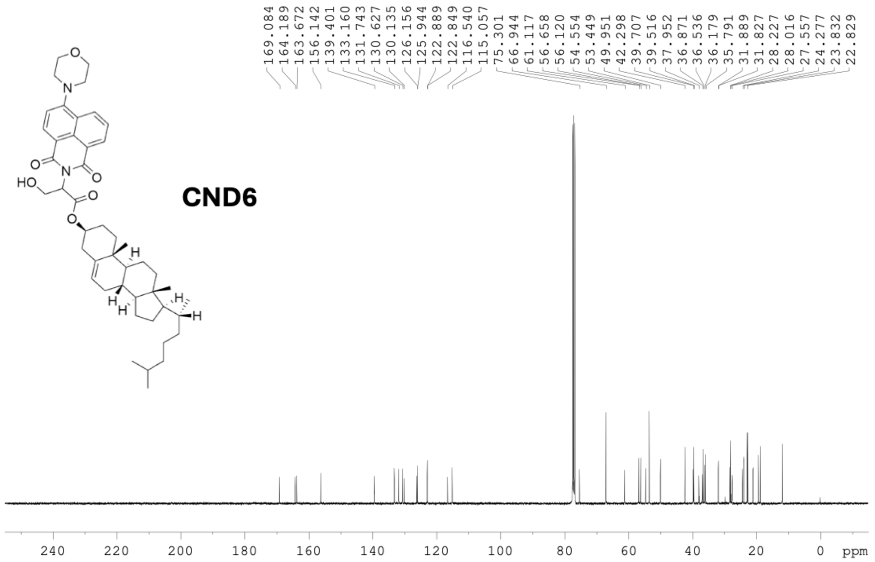
CND6
169.084
164.189
163.672
156.142
139.401
133.160
131.743
130.627
130.135
126.156
125.944
122.889
122.849
116.540
115.057
75.301
66.944
61.117
56.658
56.120
54.554
53.449
49.951
42.298
39.707
39.516
37.952
36.871
36.536
36.179
35.791
31.889
31.827
28.227
28.016
27.557
24.277
23.832
22.829
240 220 200 180 160 140 120 100 80 60 40 20 0 ppm

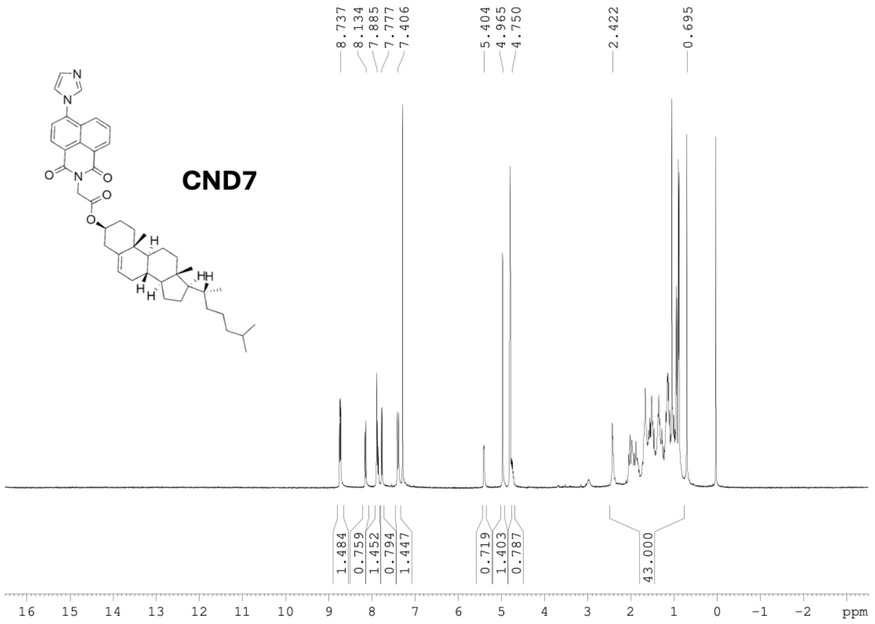
CND7
8.737
8.134
7.885
7.777
7.406
5.404
4.965
4.750
2.422
0.695
1.484
0.759
1.452
0.794
1.447
0.719
1.403
0.787
43.000
16 15 14 13 12 11 10 9 8 7 6 5 4 3 2 1 0 -1 -2 ppm

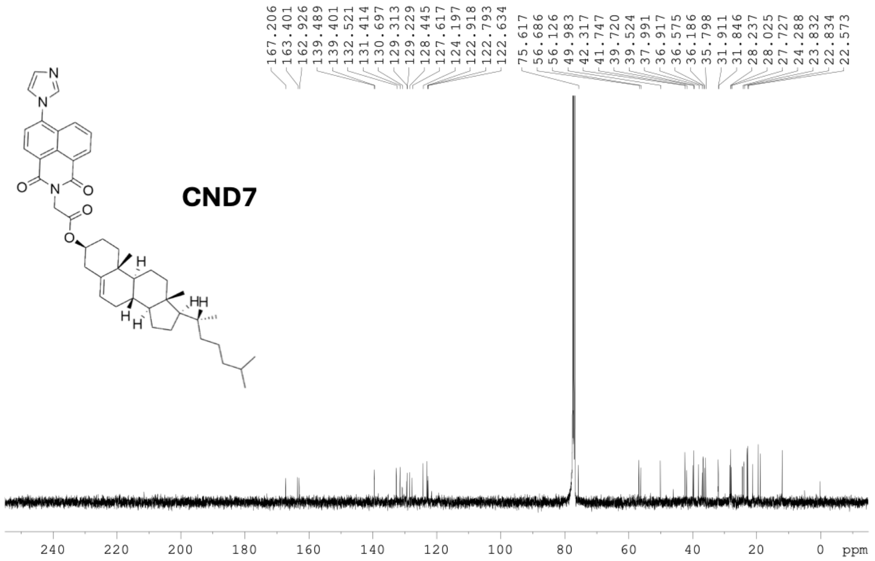
CND7
167.206
163.401
162.926
139.489
139.401
132.521
131.414
130.697
129.313
129.229
128.445
127.617
124.197
122.918
122.793
122.634
75.617
56.686
56.126
49.983
42.317
41.747
39.720
39.524
37.991
36.917
36.575
36.186
35.798
31.911
31.846
28.237
28.025
27.727
24.288
23.832
22.834
22.573
240 220 200 180 160 140 120 100 80 60 40 20 0 ppm

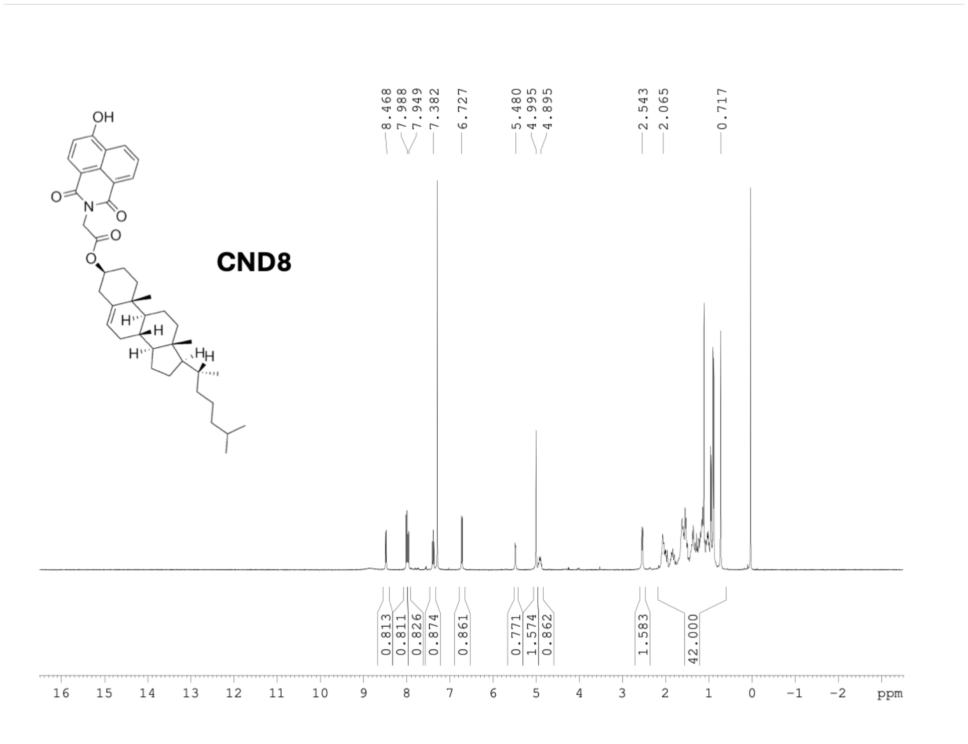
CND8
8.468
7.988
7.949
7.382
6.727
5.480
4.995
4.895
2.543
2.065
0.717
0.813
0.811
0.826
0.874
0.861
0.771
1.574
0.862
1.583
42.000
ppm

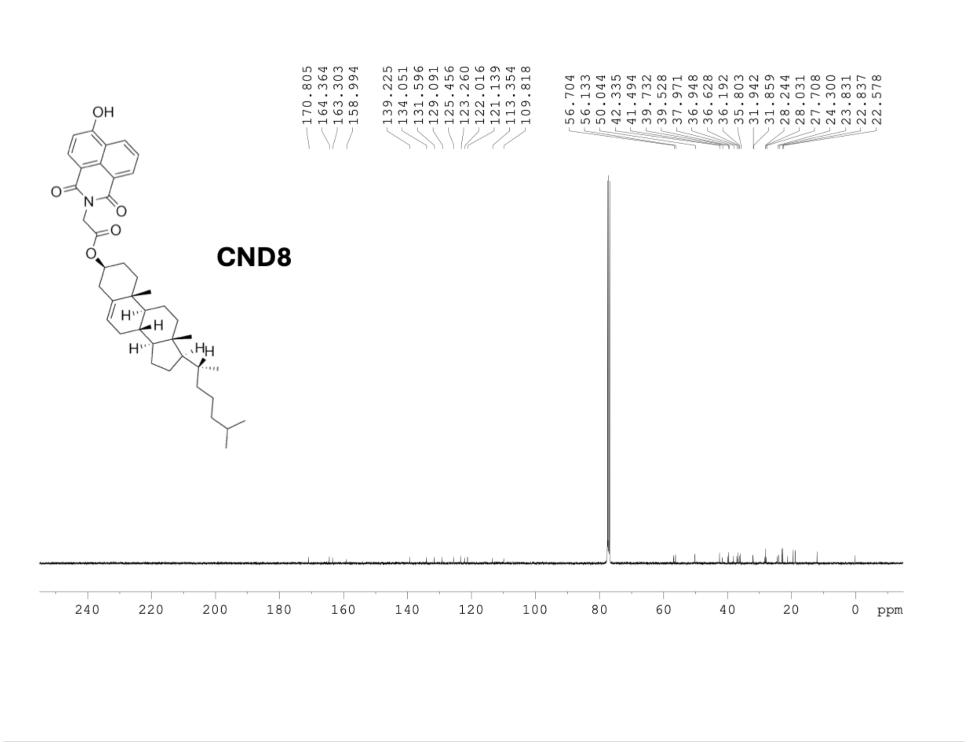
CND8
170.805
164.364
163.303
158.994
139.225
134.051
131.596
129.091
125.456
123.260
122.016
121.139
113.354
109.818
56.704
56.133
50.044
42.335
41.494
39.732
39.528
37.971
36.948
36.628
36.192
35.803
31.942
31.859
28.244
28.031
27.708
24.300
23.831
22.837
22.578
ppm

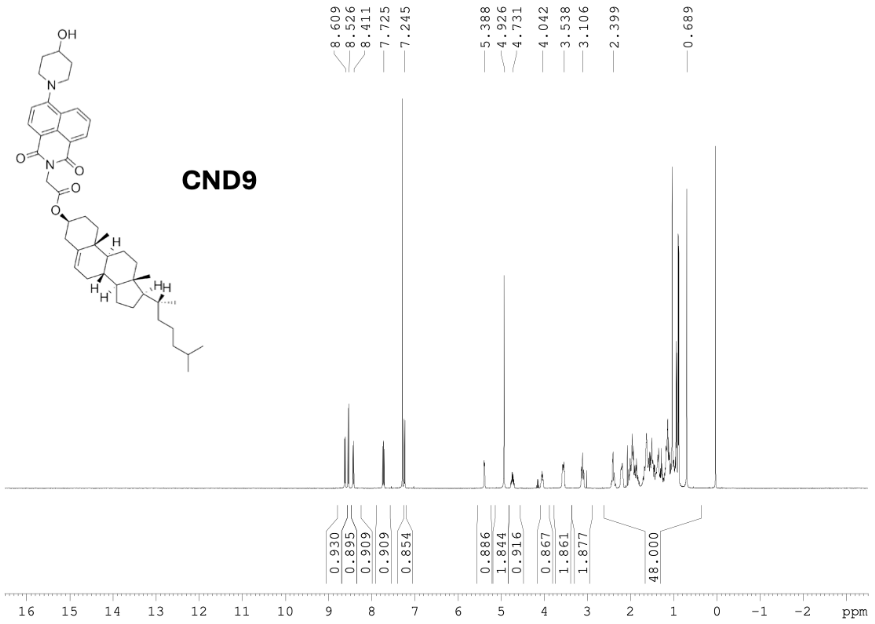
CND9
8.609
8.526
8.411
7.725
7.245
5.388
4.926
4.731
4.042
3.538
3.106
2.399
0.689
0.930
0.895
0.909
0.909
0.854
0.886
1.844
0.916
0.867
1.861
1.877
48.000
ppm

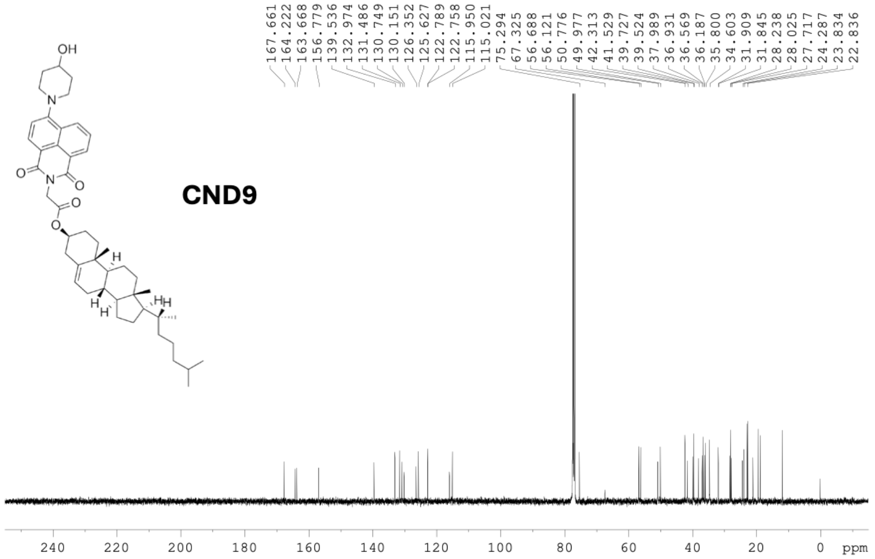
CND9
167.661
164.222
163.668
156.779
139.536
132.974
131.486
130.749
130.151
126.352
125.627
122.789
122.758
115.950
115.021
75.294
67.325
56.688
56.121
50.776
49.977
42.313
41.529
39.727
39.524
37.989
36.931
36.569
36.187
35.800
34.603
31.909
31.845
28.238
28.025
27.717
24.287
23.834
22.836
ppm

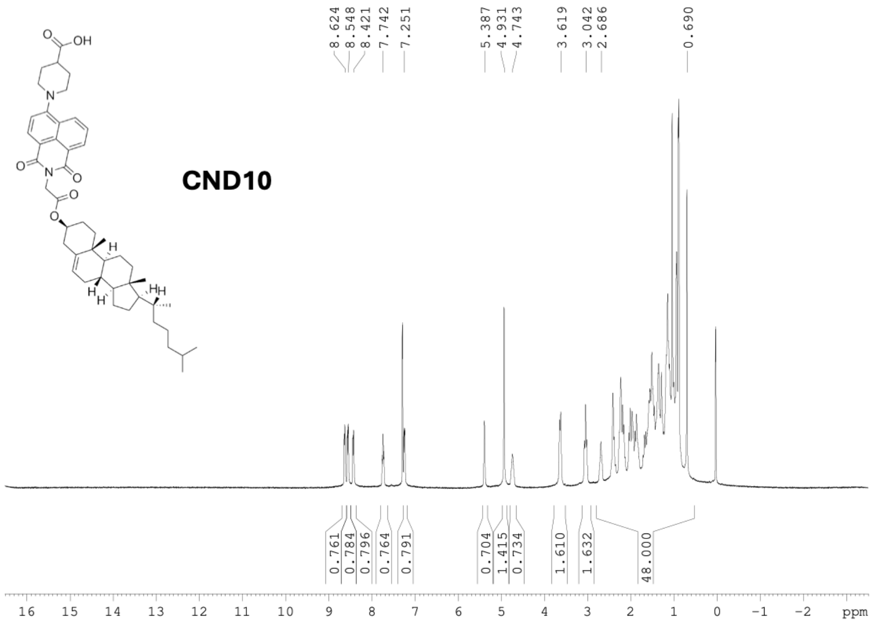

CND10
8.624
8.548
8.421
7.742
7.251
5.387
4.931
4.743
3.619
3.042
2.686
0.690
0.761
0.784
0.796
0.764
0.791
0.704
1.415
0.734
1.610
1.632
48.000
16 15 14 13 12 11 10 9 8 7 6 5 4 3 2 1 0 -1 -2 ppm

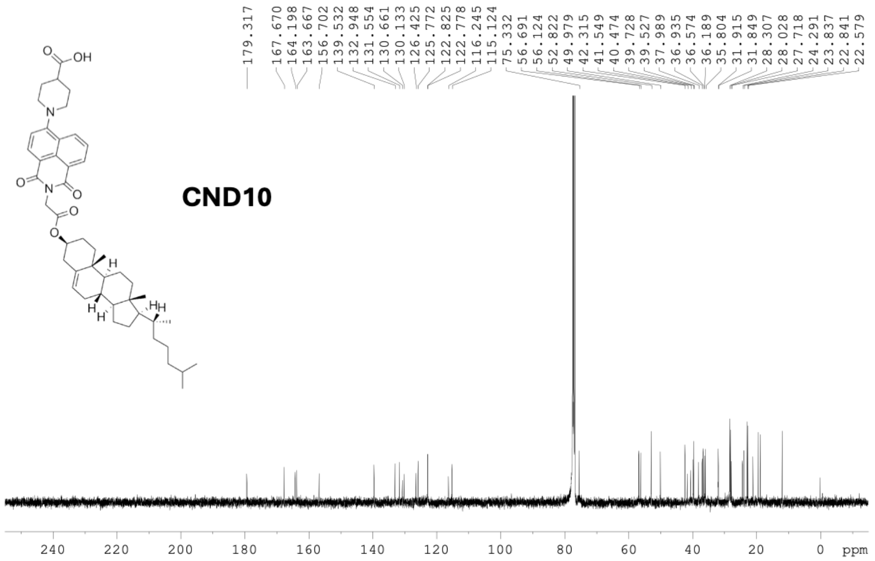

CND10
179.317
167.670
164.198
163.667
156.702
139.532
132.948
131.554
130.661
130.133
126.425
125.772
122.825
122.778
116.245
115.124
75.332
56.691
56.124
52.822
49.979
42.315
41.549
40.474
39.728
39.527
37.989
36.935
36.574
36.189
35.804
31.915
31.849
28.307
28.028
27.718
24.291
23.837
22.841
22.579
240 220 200 180 160 140 120 100 80 60 40 20 0 ppm

HR-MS spectra of all compounds

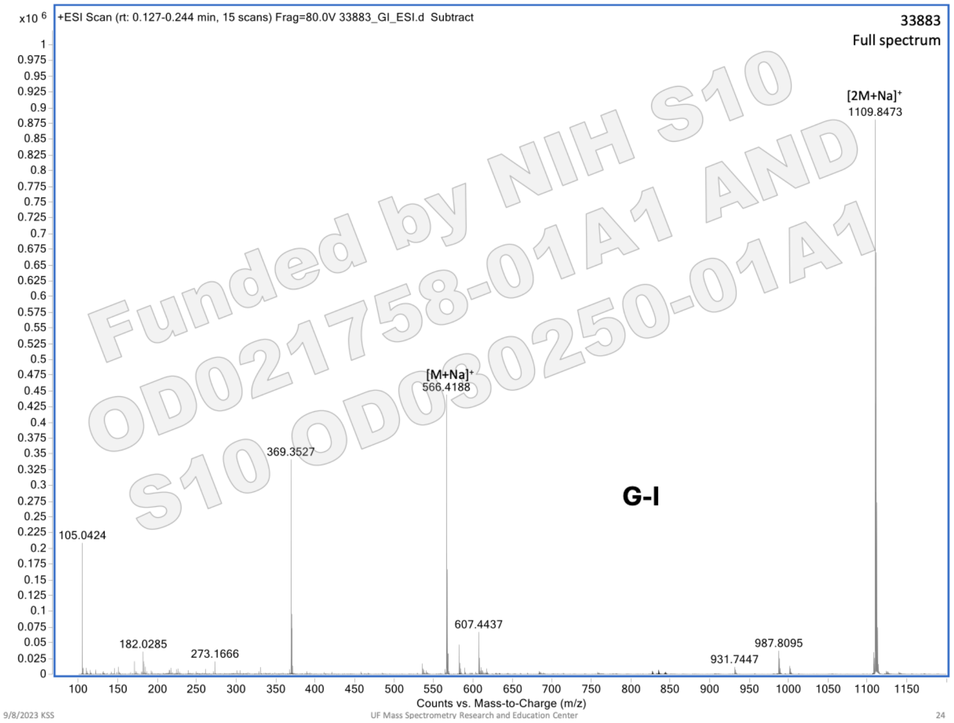

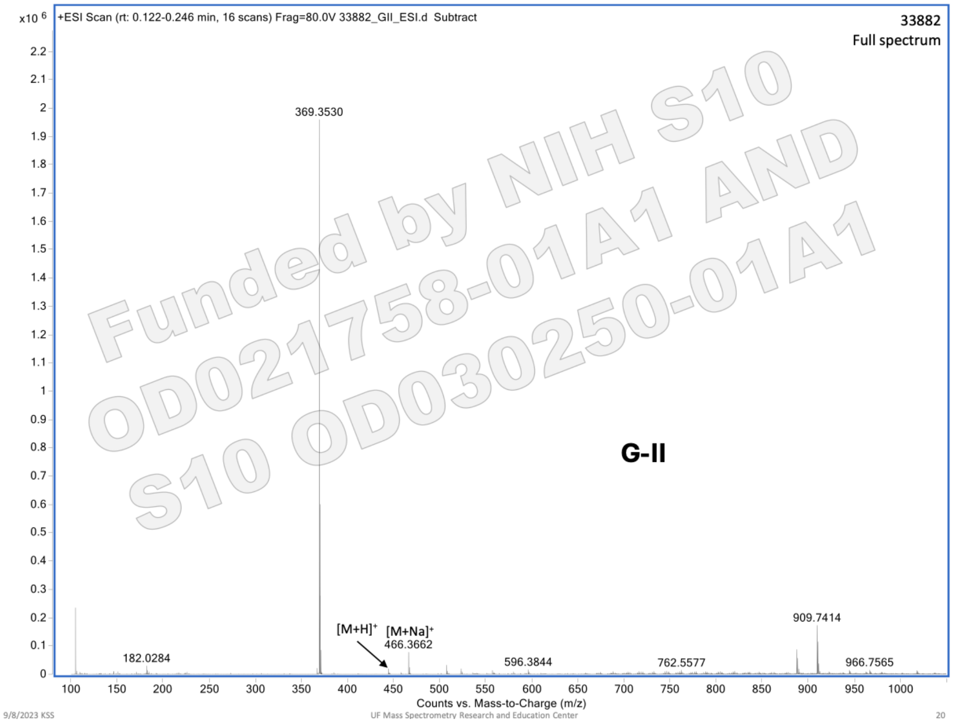

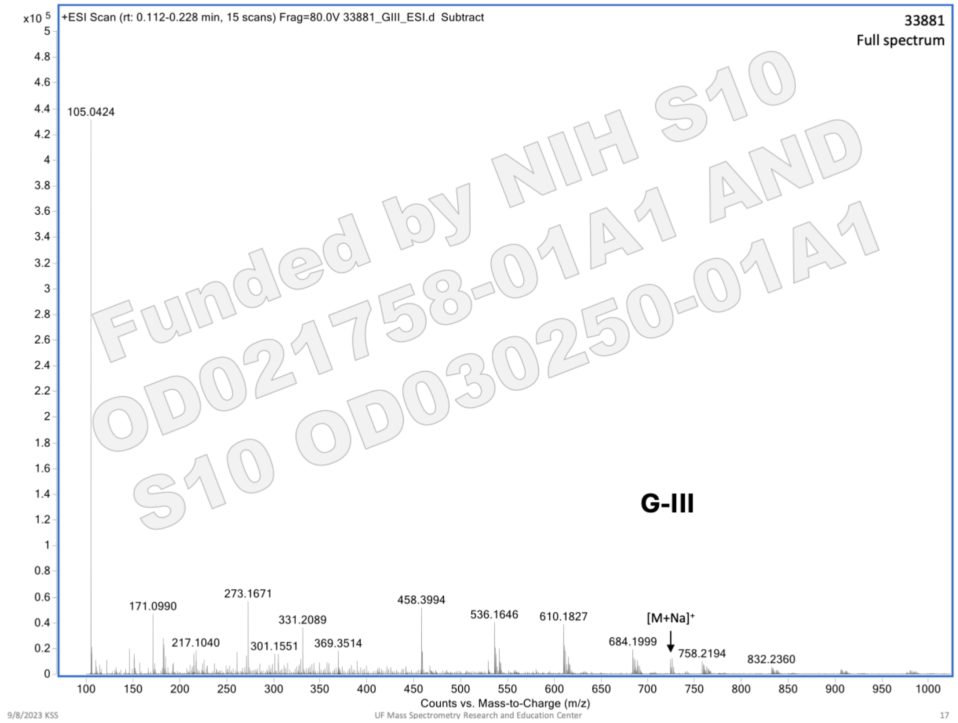
x10 5
+ESI Scan (rt: 0.112-0.228 min, 15 scans) Frag=80.0V 33881_GIII_ESI.d Subtract
33881
Full spectrum
105.0424
171.0990
273.1671
217.1040
301.1551
331.2089
369.3514
458.3994
536.1646
610.1827
684.1999
[M+Na]+
758.2194
832.2360
G-III
Counts vs. Mass-to-Charge (m/z)
Funded by NIH S10 OD021758-01A1 AND S10 OD030250-01A1
9/8/2023 KSS
UF Mass Spectrometry Research and Education Center
17

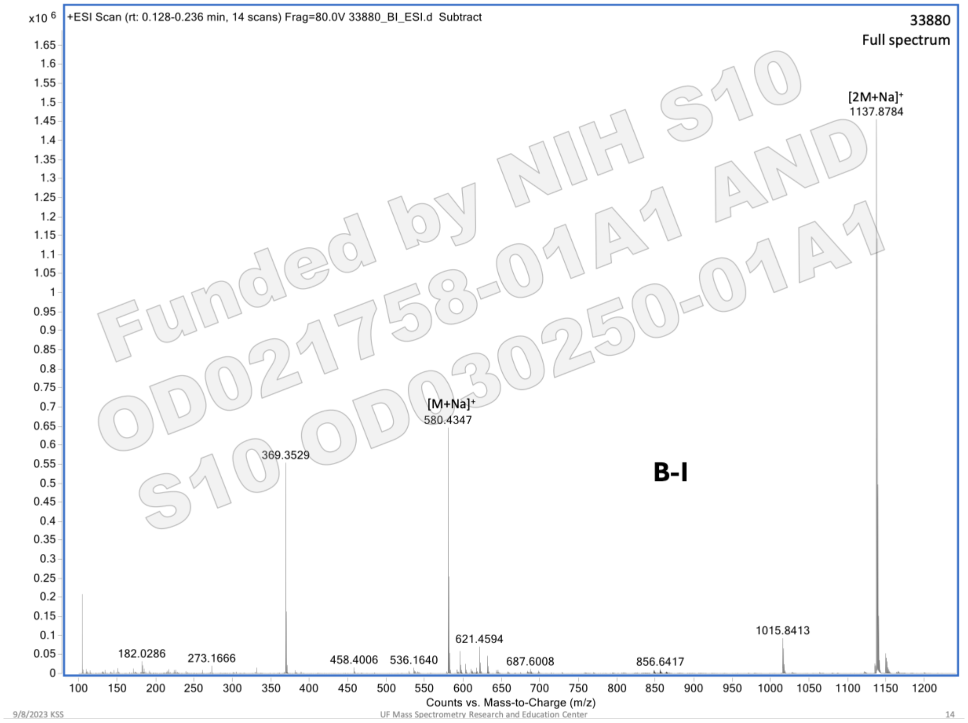
x10 6
+ESI Scan (rt: 0.128-0.236 min, 14 scans) Frag=80.0V 33880_BI_ESI.d Subtract
33880
Full spectrum
[2M+Na]+
1137.8784
[M+Na]+
580.4347
369.3529
B-I
182.0286
273.1666
458.4006
536.1640
621.4594
687.6008
856.6417
1015.8413
Counts vs. Mass-to-Charge (m/z)
Funded by NIH S10 OD021758-01A1 AND S10 OD030250-01A1
9/8/2023 KSS
UF Mass Spectrometry Research and Education Center
14

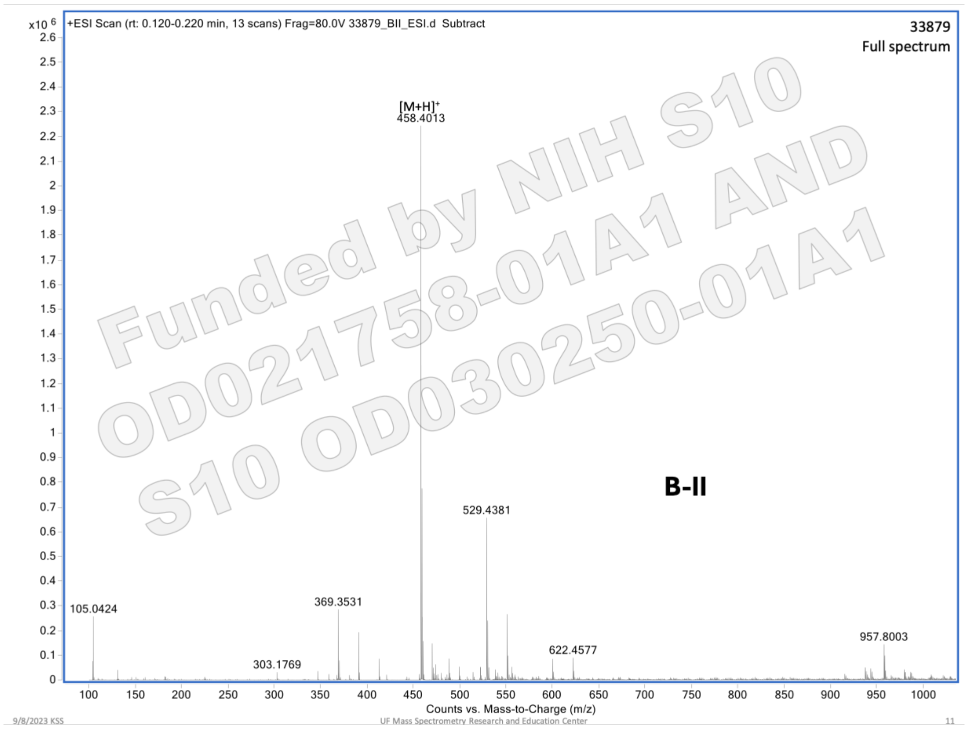
+ESI Scan (rt: 0.120-0.220 min, 13 scans) Frag=80.0V 33879_BII_ESI.d Subtract
33879
Full spectrum
x10 6
[M+H]+
458.4013
529.4381
369.3531
105.0424
303.1769
622.4577
957.8003
B-II
Counts vs. Mass-to-Charge (m/z)
Funded by NIH S10 OD021758-01A1 AND S10 OD030250-01A1
9/8/2023 KSS
UF Mass Spectrometry Research and Education Center
11

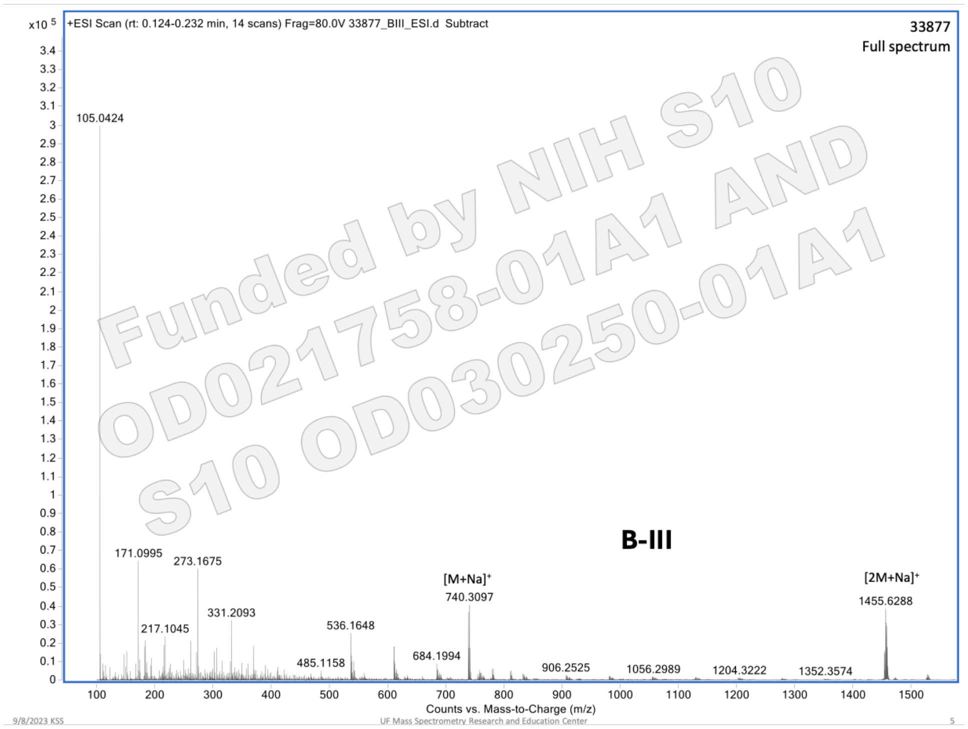

+ESI Scan (rt: 0.124-0.232 min, 14 scans) Frag=80.0V 33877_BIII_ESI.d Subtract
33877
Full spectrum
x10 5
105.0424
171.0995
273.1675
217.1045
331.2093
536.1648
485.1158
[M+Na]+
740.3097
684.1994
906.2525
1056.2989
1204.3222
1352.3574
[2M+Na]+
1455.6288
B-III
Counts vs. Mass-to-Charge (m/z)
Funded by NIH S10 OD021758-01A1 AND S10 OD030250-01A1
9/8/2023 KSS
UF Mass Spectrometry Research and Education Center
5

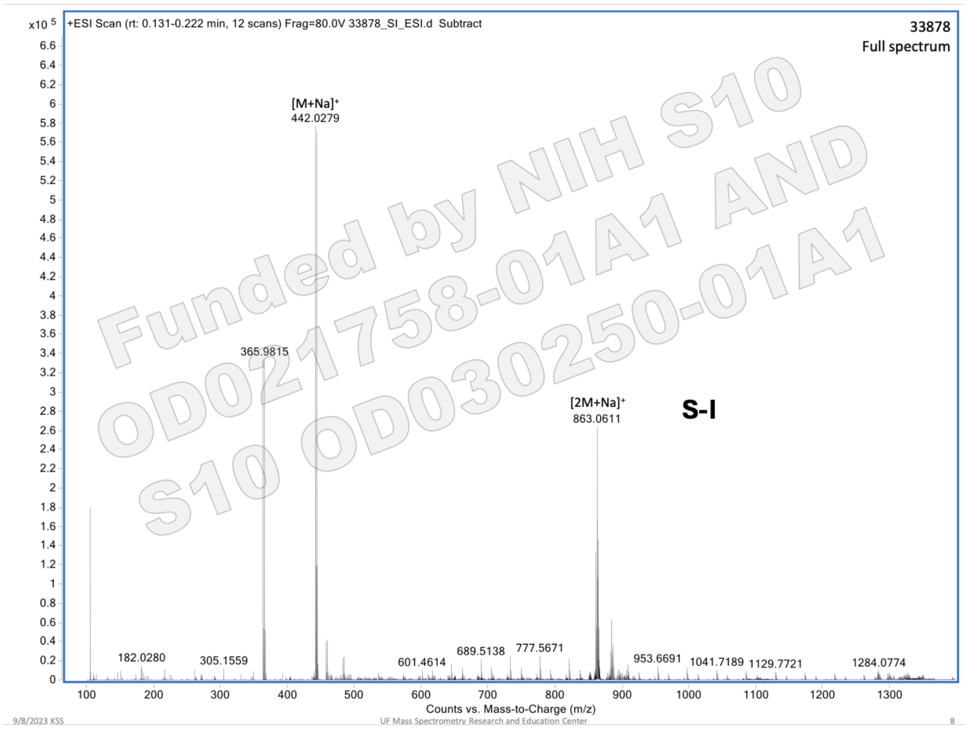

+ESI Scan (rt: 0.131-0.222 min, 12 scans) Frag=80.0V 33878_SI_ESI.d Subtract
33878
Full spectrum
x10 5
[M+Na]+
442.0279
365.9815
[2M+Na]+
863.0611
S-I
182.0280
305.1559
601.4614
689.5138
777.5671
953.6691
1041.7189
1129.7721
1284.0774
Counts vs. Mass-to-Charge (m/z)
Funded by NIH S10 OD021758-01A1 AND S10 OD030250-01A1
9/8/2023 KSS
UF Mass Spectrometry Research and Education Center
8

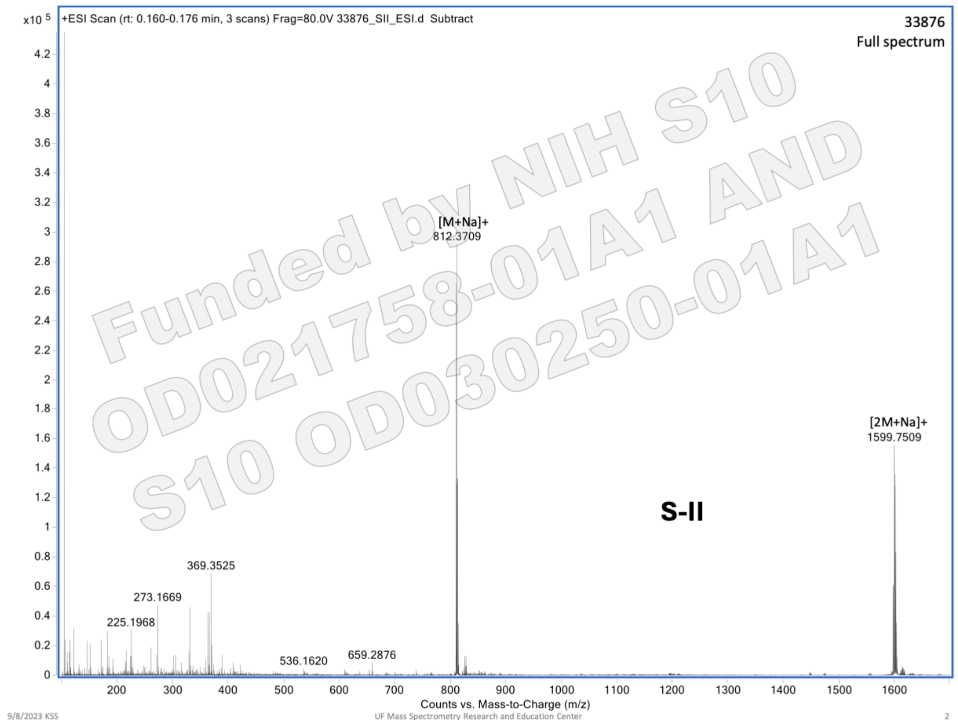

x10 5
+ESI Scan (rt: 0.160-0.176 min, 3 scans) Frag=80.0V 33876_SII_ESI.d Subtract
33876
Full spectrum
[M+Na]+
812.3709
[2M+Na]+
1599.7509
S-II
369.3525
273.1669
225.1968
536.1620
659.2876
Counts vs. Mass-to-Charge (m/z)
9/8/2023 KSS
UF Mass Spectrometry Research and Education Center
2

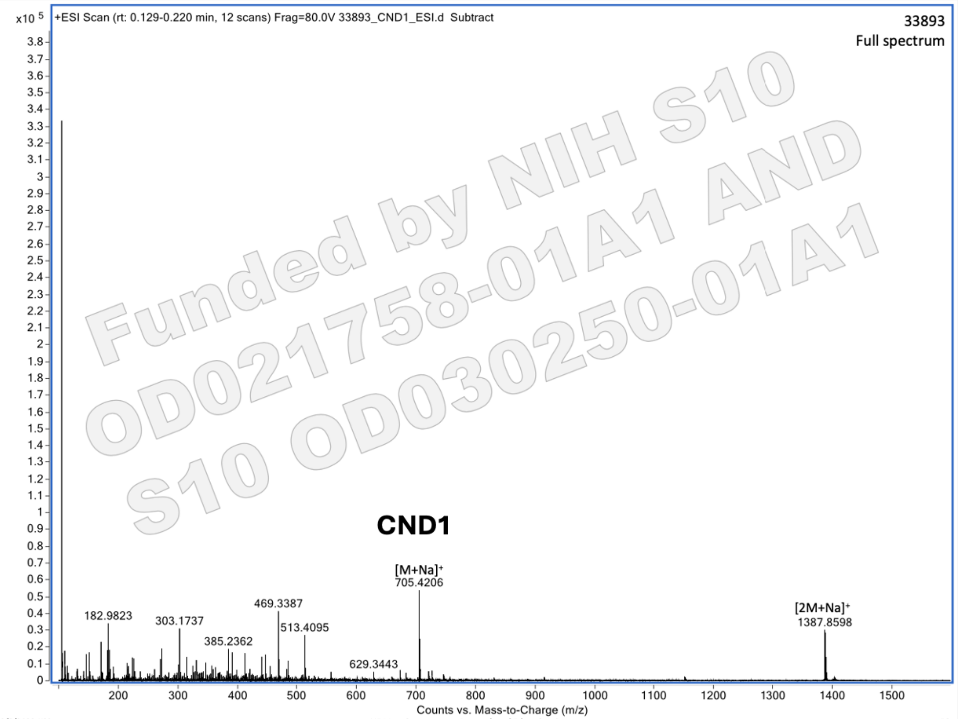

x10 5
+ESI Scan (rt: 0.129-0.220 min, 12 scans) Frag=80.0V 33893_CND1_ESI.d Subtract
33893
Full spectrum
CND1
[M+Na]+
705.4206
[2M+Na]+
1387.8598
182.9823
303.1737
385.2362
469.3387
513.4095
629.3443
Counts vs. Mass-to-Charge (m/z)

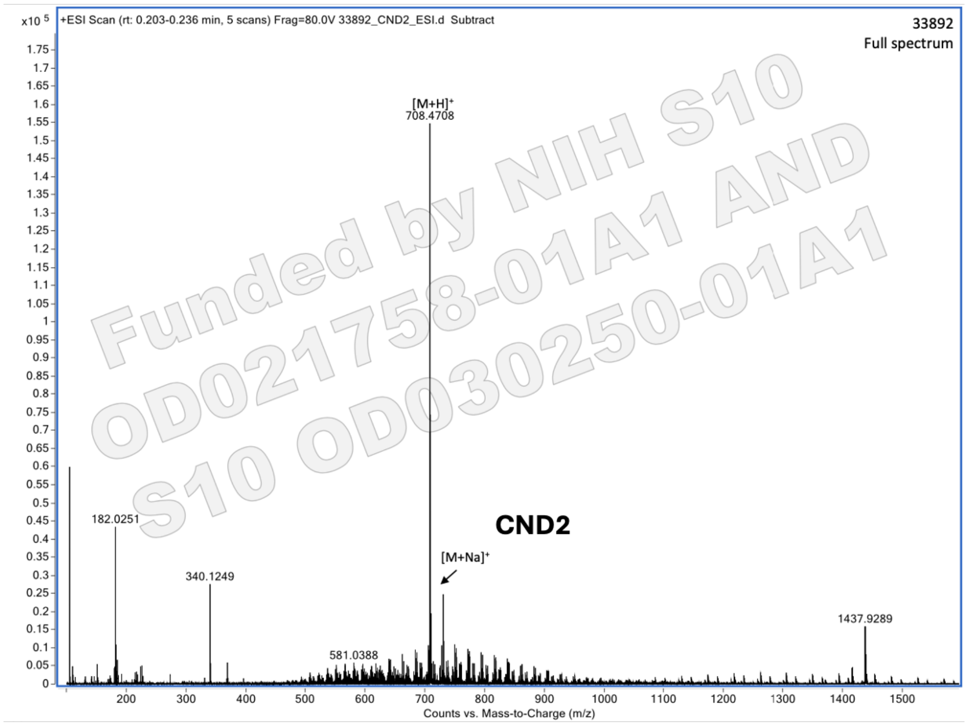

+ESI Scan (rt: 0.203-0.236 min, 5 scans) Frag=80.0V 33892_CND2_ESI.d Subtract
33892
Full spectrum
[M+H]+
708.4708
182.0251
340.1249
581.0388
CND2
[M+Na]+
1437.9289
Counts vs. Mass-to-Charge (m/z)
Funded by NIH S10 OD021758-01A1 AND S10 OD030250-01A1

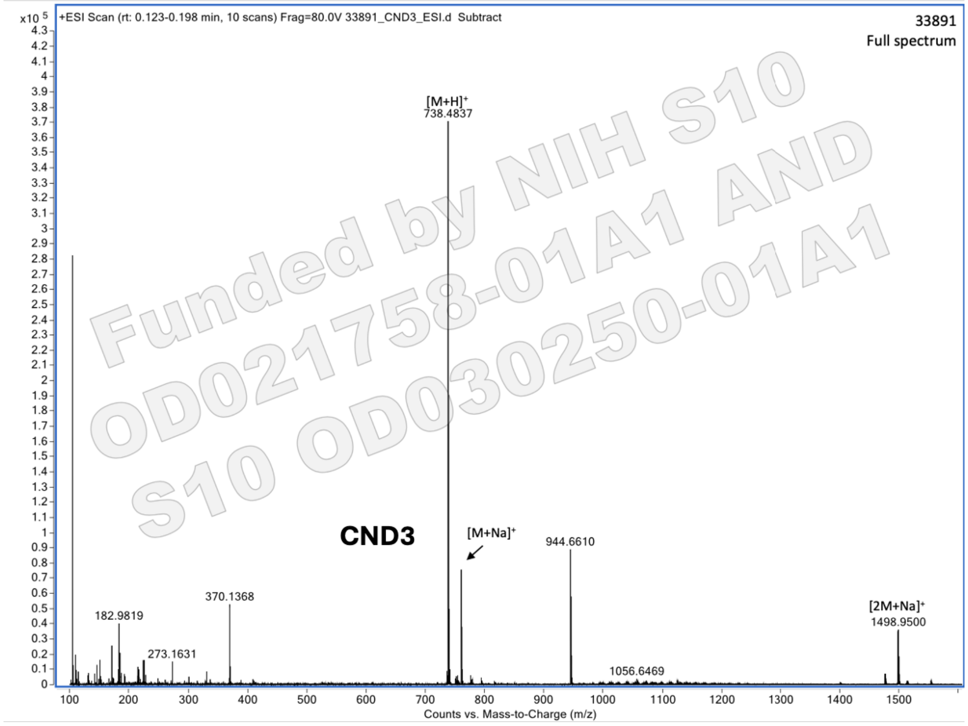

+ESI Scan (rt: 0.123-0.198 min, 10 scans) Frag=80.0V 33891_CND3_ESI.d Subtract
33891
Full spectrum
[M+H]+
738.4837
CND3
[M+Na]+
944.6610
370.1368
182.9819
273.1631
1056.6469
[2M+Na]+
1498.9500
Counts vs. Mass-to-Charge (m/z)
Funded by NIH S10 OD021758-01A1 AND S10 OD030250-01A1

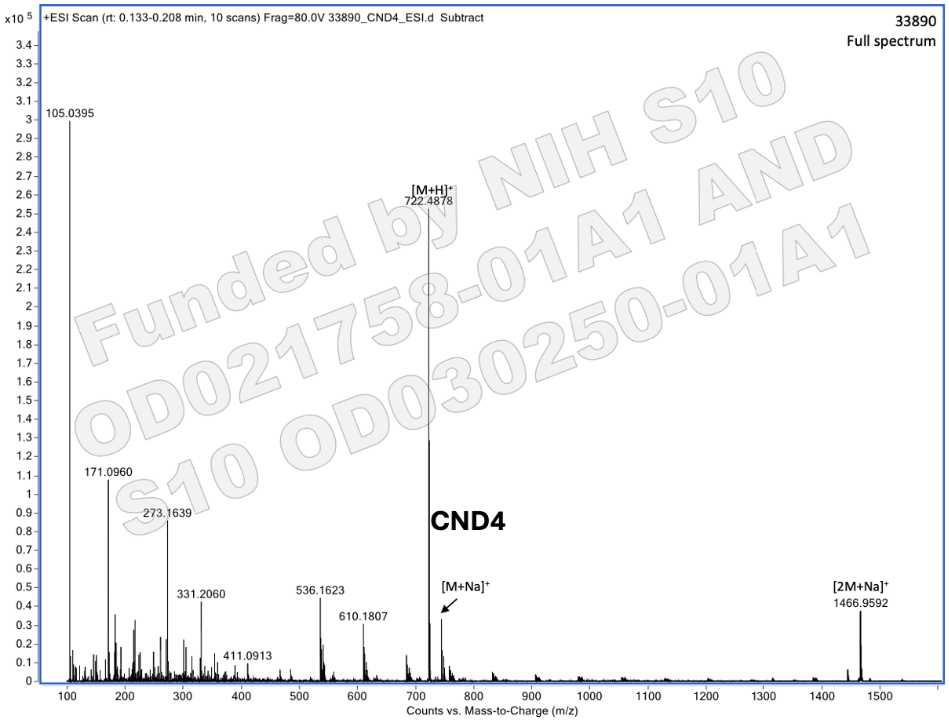

Funded by NIH S10 OD021758-01A1 AND S10 OD030250-01A1

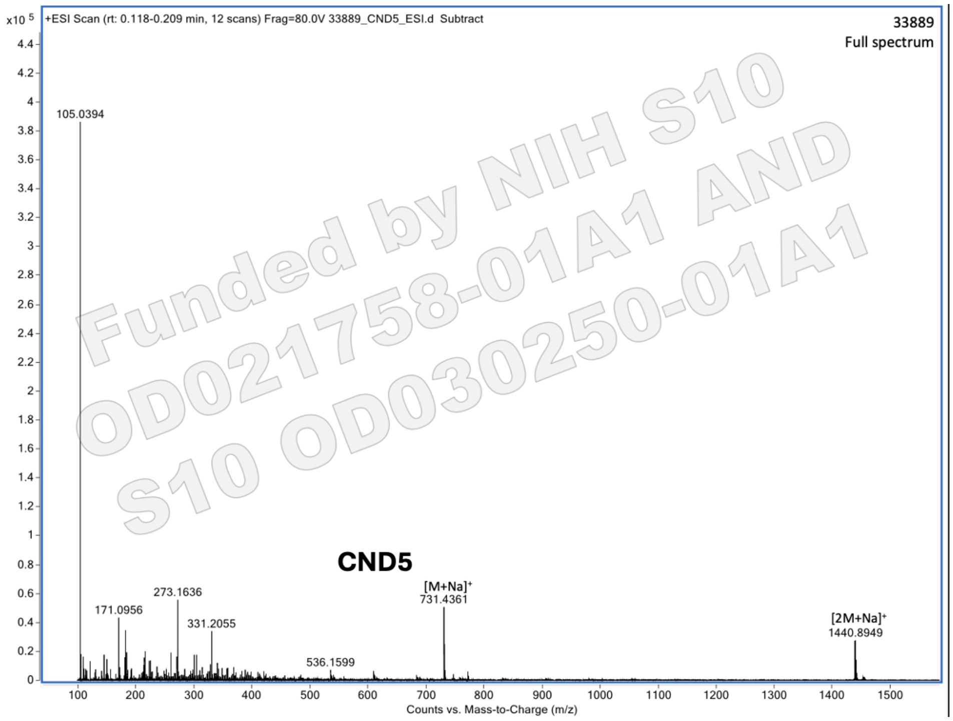

Funded by NIH S10 OD021758-01A1 AND S10 OD030250-01A1

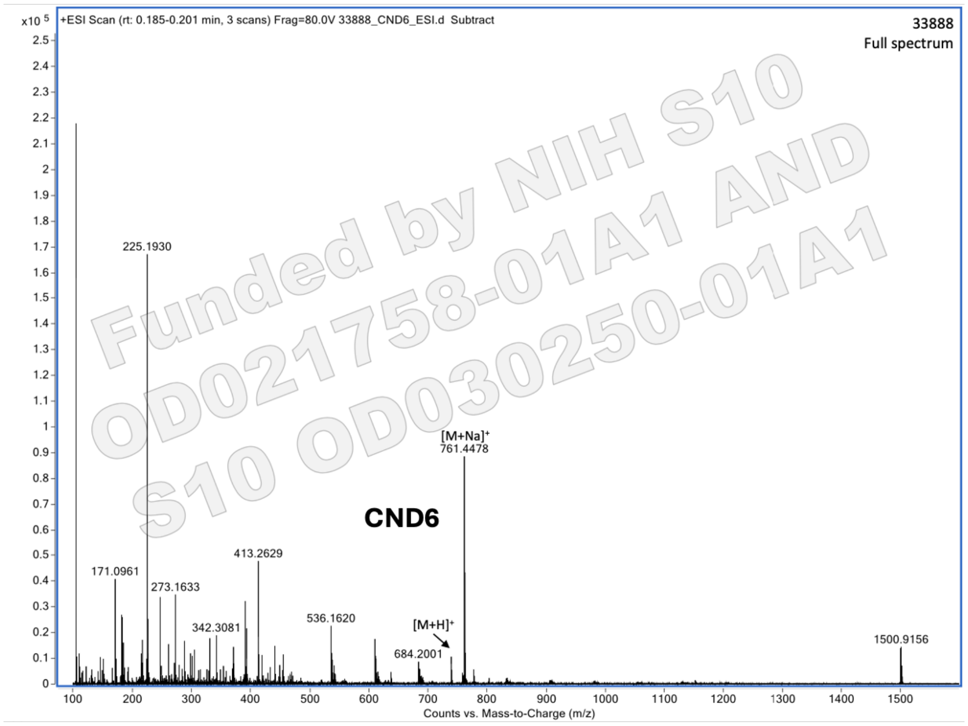
+ESI Scan (rt: 0.185-0.201 min, 3 scans) Frag=80.0V 33888_CND6_ESI.d Subtract
33888
Full spectrum
x10 5
CND6
[M+Na]+
761.4478
[M+H]+
684.2001
225.1930
171.0961
273.1633
342.3081
413.2629
536.1620
1500.9156
Counts vs. Mass-to-Charge (m/z)
Funded by NIH S10 OD021758-01A1 AND S10 OD030250-01A1

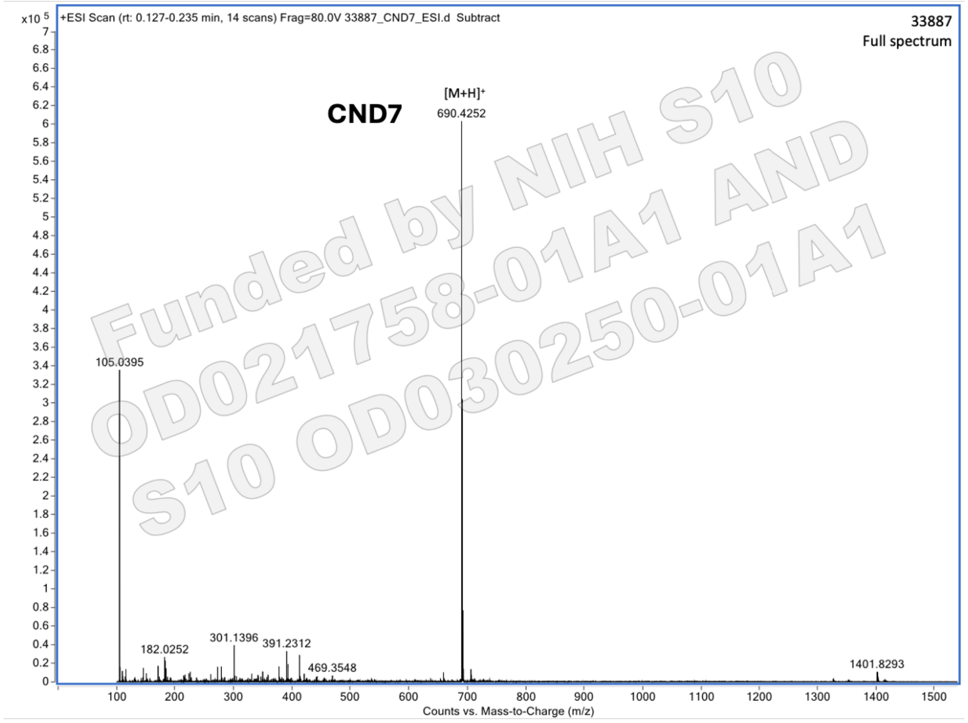
+ESI Scan (rt: 0.127-0.235 min, 14 scans) Frag=80.0V 33887_CND7_ESI.d Subtract
33887
Full spectrum
x10 5
CND7
[M+H]+
690.4252
105.0395
182.0252
301.1396
391.2312
469.3548
1401.8293
Counts vs. Mass-to-Charge (m/z)
Funded by NIH S10 OD021758-01A1 AND S10 OD030250-01A1

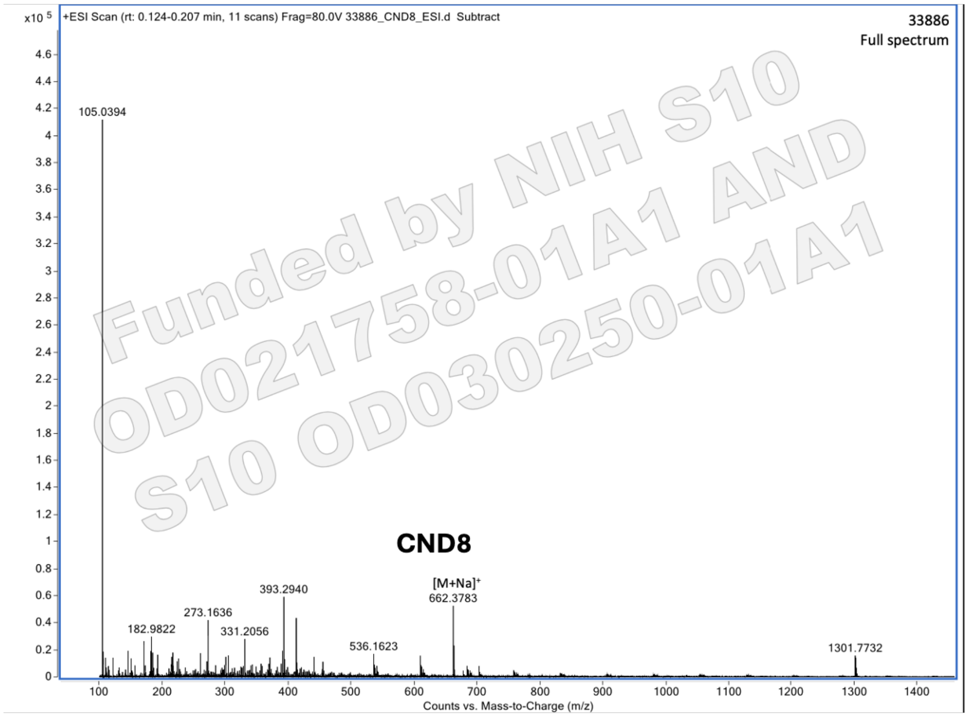

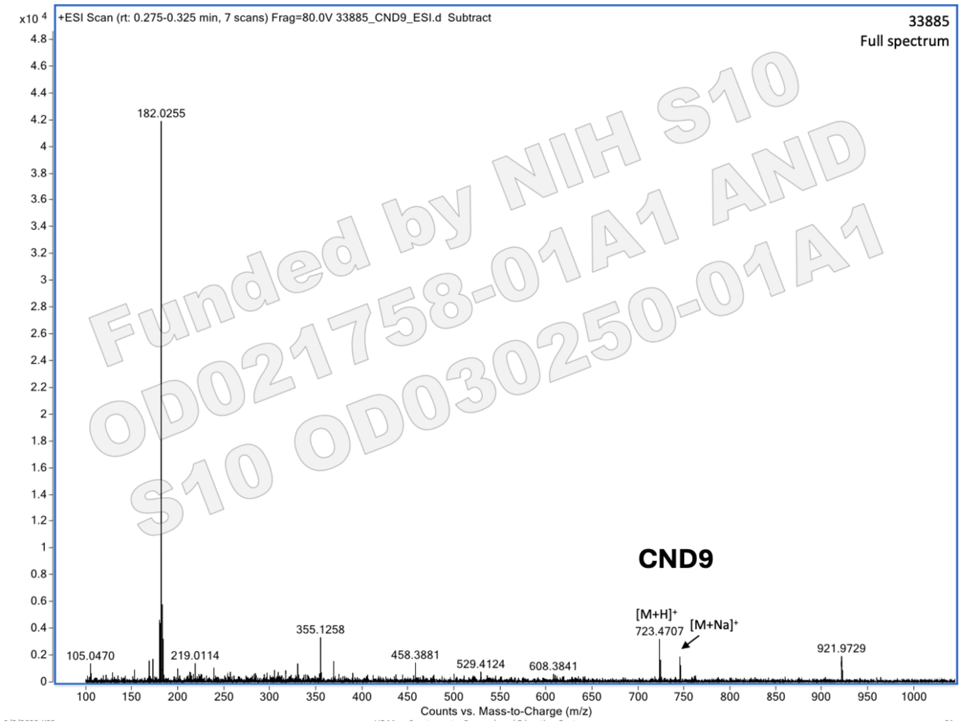

Funded by NIH S10 OD021758-01A1 AND S10 OD030250-01A1

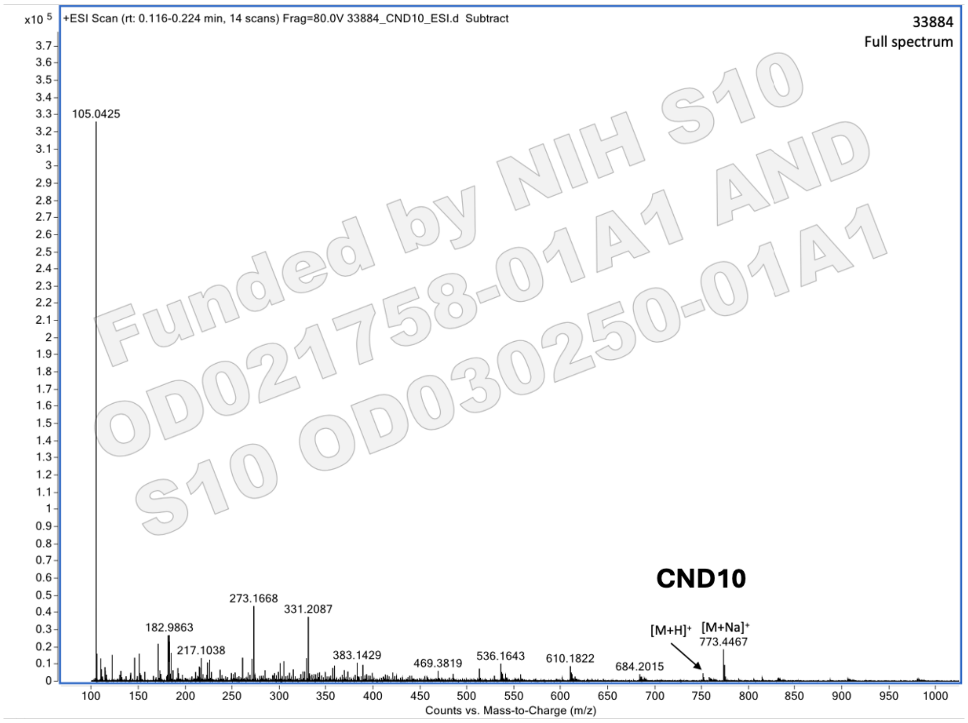
+ESI Scan (rt: 0.116-0.224 min, 14 scans) Frag=80.0V 33884_CND10_ESI.d Subtract
33884
Full spectrum
x10 5
105.0425
182.9863
217.1038
273.1668
331.2087
383.1429
469.3819
536.1643
610.1822
684.2015
[M+H]+
[M+Na]+
773.4467
CND10
Funded by NIH S10 OD021758-01A1 AND S10 OD030250-01A1
Counts vs. Mass-to-Charge (m/z)

References

(1) Pyne, A.; Kuchlyan, J.; Maiti, C.; Dhara, Di.; Sarkar, N. Cholesterol Based Surface Active Ionic Liquid That Can Form Microemulsions and Spontaneous Vesicles. *Langmuir* **2017**, *33* (23), 5891–5899. https://doi.org/10.1021/acs.langmuir.7b01158.

(2) Mataga, N.; Kaifu, Y.; Kolzumi, M. Solvent Effects upon Fluorescence Spectra and the Dipole Moments of Excited Molecules. *Bull Chem Soc Jpn* **1956**, *29*, 465–470.

(3) Lakowicz, J. *Principles of Fluorescence Spectroscopy*, 3rd ed.; Lakowicz, J., Ed.; Springer : New York, NY, 2006.

(4) Hoelzel, C. A.; Hu, H.; Wolstenholme, C. H.; Karim, B. A.; Munson, K. T.; Jung, K. H.; Zhang, H.; Liu, Y.; Yennawar, H. P.; Asbury, J. B.; Li, X.; Zhang, X. A General Strategy to Enhance Donor-Acceptor Molecules Using Solvent-Excluding Substituents. *Angewandte Chemie - International Edition* **2020**, *59* (12), 4785–4792. https://doi.org/10.1002/anie.201915744.

(5) Jackman, J. A.; Cho, N.-J. Supported Lipid Bilayer Formation: Beyond Vesicle Fusion. *Langmuir* **2020**, *36* (6), 1387–1400. https://doi.org/10.1021/acs.langmuir.9b03706.

(6) Nair, K. S.; Bajaj, H. Advances in Giant Unilamellar Vesicle Preparation Techniques and Applications. *Adv Colloid Interface Sci* **2023**, *318*, 102935. https://doi.org/10.1016/j.cis.2023.102935.

(7) Gerstle, Z.; Desai, R.; Veatch, S. L. Giant Plasma Membrane Vesicles: An Experimental Tool for Probing the Effects of Drugs and Other Conditions on Membrane Domain Stability. In *Methods in Enzymology*; 2018; pp 129–150. https://doi.org/10.1016/bs.mie.2018.02.007.

(8) Baumgart, T.; Hunt, G.; Farkas, E. R.; Webb, W. W.; Feigenson, G. W. Fluorescence Probe Partitioning between Lo/Ld Phases in Lipid Membranes. *Biochim Biophys Acta Biomembr* **2007**, *1768* (9), 2182–2194. https://doi.org/10.1016/j.bbamem.2007.05.012.

(9) Ariola, F. S.; Li, Z.; Cornejo, C.; Bittman, R.; Heikal, A. A. Membrane Fluidity and Lipid Order in Ternary Giant Unilamellar Vesicles Using a New Bodipy-Cholesterol Derivative. *Biophys J* **2009**, *96* (7), 2696–2708. https://doi.org/10.1016/j.bpj.2008.12.3922.

(10) Sezgin, E.; Levental, I.; Grzybek, M.; Schwarzmann, G.; Mueller, V.; Honigmann, A.; Belov, V. N.; Eggeling, C.; Coskun, Ü.; Simons, K.; Schwille, P. Partitioning, Diffusion, and Ligand Binding of Raft Lipid Analogs in Model and Cellular Plasma Membranes. *Biochim Biophys Acta Biomembr* **2012**, *1818* (7), 1777–1784. https://doi.org/10.1016/j.bbamem.2012.03.007.

(11) Sezgin, E.; Can, F. B.; Schneider, F.; Clausen, M. P.; Galiani, S.; Stanly, T. A.; Waithe, D.; Colaco, A.; Honigmann, A.; Wüstner, D.; Platt, F.; Eggeling, C. A Comparative Study on Fluorescent Cholesterol Analogs as Versatile Cellular Reporters. *J Lipid Res* **2016**, *57* (2), 299–309. https://doi.org/10.1194/jlr.M065326.

(12) Ramirez, D. M. C.; Ogilvie, W. W.; Johnston, L. J. NBD-Cholesterol Probes to Track Cholesterol Distribution in Model Membranes. *Biochim Biophys Acta Biomembr* **2010**, *1798* (3), 558–568. https://doi.org/10.1016/j.bbamem.2009.12.005.

(13) Sych, T.; Schubert, T.; Vauchelles, R.; Madl, J.; Omidvar, R.; Thuenauer, R.; Richert, L.; Mély, Y.; Römer, W. GUV-AP: Multifunctional Fiji-Based Tool for Quantitative Image

Analysis of Giant Unilamellar Vesicles. *Bioinformatics* **2019**, *35* (13), 2340–2342. https://doi.org/10.1093/bioinformatics/bty962.
(14) Kim, S.; Lee, J.; Jo, S.; Brooks, C. L.; Lee, H. S.; Im, W. CHARMM-GUI Ligand Reader and Modeler for CHARMM Force Field Generation of Small Molecules. *J Comput Chem* **2017**, *38* (21), 1879–1886. https://doi.org/10.1002/jcc.24829.
(15) Brooks, B. R.; Brooks, C. L.; Mackerell, A. D.; Nilsson, L.; Petrella, R. J.; Roux, B.; Won, Y.; Archontis, G.; Bartels, C.; Boresch, S.; Caflisch, A.; Caves, L.; Cui, Q.; Dinner, A. R.; Feig, M.; Fischer, S.; Gao, J.; Hodoscek, M.; Im, W.; Kuczera, K.; Lazaridis, T.; Ma, J.; Ovchinnikov, V.; Paci, E.; Pastor, R. W.; Post, C. B.; Pu, J. Z.; Schaefer, M.; Tidor, B.; Venable, R. M.; Woodcock, H. L.; Wu, X.; Yang, W.; York, D. M.; Karplus, M. CHARMM: The Biomolecular Simulation Program. *J Comput Chem* **2009**, *30* (10), 1545–1614. https://doi.org/10.1002/jcc.21287.
(16) Jo, S.; Kim, T.; Iyer, V. G.; Im, W. CHARMM-GUI: A Web-Based Graphical User Interface for CHARMM. *J Comput Chem* **2008**, *29* (11), 1859–1865. https://doi.org/10.1002/jcc.20945.
(17) Vanommeslaeghe, K.; Hatcher, E.; Acharya, C.; Kundu, S.; Zhong, S.; Shim, J.; Darian, E.; Guvench, O.; Lopes, P.; Vorobyov, I.; Mackerell, A. D. CHARMM General Force Field: A Force Field for Drug-like Molecules Compatible with the CHARMM All-atom Additive Biological Force Fields. *J Comput Chem* **2010**, *31* (4), 671–690. https://doi.org/10.1002/jcc.21367.
(18) Wu, E. L.; Cheng, X.; Jo, S.; Rui, H.; Song, K. C.; Dávila-Contreras, E. M.; Qi, Y.; Lee, J.; Monje-Galvan, V.; Venable, R. M.; Klauda, J. B.; Im, W. CHARMM-GUI Membrane Builder toward Realistic Biological Membrane Simulations. *Journal of Computational Chemistry*. John Wiley and Sons Inc. October 15, 2014, pp 1997–2004. https://doi.org/10.1002/jcc.23702.
(19) Abraham, M. J.; Murtola, T.; Schulz, R.; Páll, S.; Smith, J. C.; Hess, B.; Lindah, E. GROMACS: High Performance Molecular Simulations through Multi-Level Parallelism from Laptops to Supercomputers. *SoftwareX* **2015**, *1–2*, 19–25. https://doi.org/10.1016/J.SOFTX.2015.06.001.
(20) Hess, B.; Bekker, H.; Berendsen, H. J. C.; Fraaije, J. G. E. M. *LINCS: A Linear Constraint Solver for Molecular Simulations*; John Wiley & Sons, Inc, 1997; Vol. 18.
(21) Berendsen, H. J. C.; Postma, J. P. M.; van Gunsteren, W. F.; Dinola, A.; Haak, J. R. Molecular Dynamics with Coupling to an External Bath. *J Chem Phys* **1984**, *81* (8), 3684–3690. https://doi.org/10.1063/1.448118.
(22) Humphrey, W.; Dalke, A.; Schulten, K. *VMD: Visual Molecular Dynamics*; 1996.
(23) Guixà-González, R.; Rodriguez-Espigares, I.; Ramírez-Anguita, J. M.; Carrió-Gaspar, P.; Martinez-Seara, H.; Giorgino, T.; Selent, J. MEMBPLUGIN: Studying Membrane Complexity in VMD. *Bioinformatics* **2014**, *30* (10), 1478–1480. https://doi.org/10.1093/bioinformatics/btu037.
(24) Schindelin, J.; Arganda-Carreras, I.; Frise, E.; Kaynig, V.; Longair, M.; Pietzsch, T.; Preibisch, S.; Rueden, C.; Saalfeld, S.; Schmid, B.; Tinevez, J. Y.; White, D. J.; Hartenstein, V.; Eliceiri, K.; Tomancak, P.; Cardona, A. Fiji: An Open-Source Platform for Biological-Image Analysis. *Nat Methods* **2012**, *9* (7), 676–682. https://doi.org/10.1038/nmeth.2019.
(25) Arganda-Carreras, I.; Kaynig, V.; Rueden, C.; Eliceiri, K. W.; Schindelin, J.; Cardona, A.; Seung, H. S. Trainable Weka Segmentation: A Machine Learning Tool for Microscopy Pixel

Classification. *Bioinformatics* **2017**, *33* (15), 2424–2426. https://doi.org/10.1093/bioinformatics/btx180.
(26) Dunn, K. W.; Kamocka, M. M.; McDonald, J. H. A Practical Guide to Evaluating Colocalization in Biological Microscopy. *Am J Physiol Cell Physiol* **2011**, *300* (4), C723–C742. https://doi.org/10.1152/ajpcell.00462.2010.
(27) Costes, S. V.; Daelemans, D.; Cho, E. H.; Dobbin, Z.; Pavlakis, G.; Lockett, S. Automatic and Quantitative Measurement of Protein-Protein Colocalization in Live Cells. *Biophys J* **2004**, *86* (6), 3993–4003. https://doi.org/10.1529/biophysj.103.038422.
(28) Stauffer, W.; Sheng, H.; Lim, H. N. EzColocalization: An ImageJ Plugin for Visualizing and Measuring Colocalization in Cells and Organisms. *Sci Rep* **2018**, *8* (1), 15764. https://doi.org/10.1038/s41598-018-33592-8.
